# Renal adverse events in EGFR-TKI treatment: Comprehensive characterization of clinical patterns and molecular underpinnings

**DOI:** 10.1016/j.gendis.2025.101953

**Published:** 2025-11-28

**Authors:** Huili Xiao, Anqi Lin, Wentao Xu, Junyi Shen, Cuiying Chen, Jian Zhang, Ting Wei, Peng Luo

**Affiliations:** Department of Oncology, Zhujiang Hospital, Southern Medical University, Guangzhou, Guangdong 510282, China

Epidermal growth factor receptor-tyrosine kinase inhibitors (EGFR-TKIs), as vital targeted therapies in cancer treatment, show significant clinical effectiveness but raise increasing safety concerns. Current safety studies mainly focus on dermatologic, hepatic, and pulmonary toxicities.^1^ The common toxicities of EGFR-TKIs (*e.g.*, rash, diarrhea, interstitial lung disease) are more noticeable and often reported, so they have traditionally received more attention in clinical trials and guidelines; this has somewhat overshadowed renal adverse reactions, which are less common or more subtle. Moreover, early signs of nephrotoxicity are mostly non-specific; additionally, cancer patients often have pre-existing kidney disease or are on other nephrotoxic chemotherapies, making nephrotoxicity challenging to diagnose accurately. Furthermore, initial EGFR-TKI trials primarily focused on efficacy outcomes, with limited follow-up and no specific renal function monitoring included in the protocols, resulting in inadequate data on the incidence and characteristics of nephrotoxicity.

In this study, we characterized renal adverse drug reactions associated with EGFR-TKIs in cancer patients using the US Food and Drug Administration (FDA) Adverse Event Reporting System (FAERS) and the World Health Organization (WHO) Global Individual Case Safety Reports Database (VigiBase). Public database findings were further validated through reverse transcription-quantitative polymerase chain reaction (RT-qPCR) experiments. Additionally, we investigated the underlying molecular mechanisms by integrating pan-cancer transcriptomic data from The Cancer Genome Atlas (TCGA) with renal tissue transcriptome sequencing data from EGFR-TKI-treated mouse models.

We identified 30,923 and 50,896 case reports of cancer patients with EGFR-TKIs listed as the primary suspected (PS) drugs from the FAERS and VigiBase databases, respectively. Among these, 716 and 1006 cases developed renal adverse events. Clinical characteristics of all analyzed patients are presented in Table S1–S4 and Figure S1A–S1D. Within renal/urinary system disorders, kidney-related preferred terms made up the majority (Fig. S1E and S1F). Renal adverse events accounted for only a small part of overall adverse events (2.3% and 2.0%, respectively) (Fig. S2A and S2B). The yearly percentage of renal adverse event cases among total cases remained steady, ranging from 1.0% to 4.0%. Erlotinib was the most often reported drug in renal adverse event cases (Fig. S2C and S2D). Acute kidney injury, renal failure, and renal impairment were the top three reported renal preferred terms (Fig. S2E and S2F). Erlotinib and afatinib accounted for most renal adverse event reports across several categories (Fig. S3A). Notably, over 85% of renal adverse event cases occurred alongside non-renal preferred terms, mainly diarrhea (36.73%, 41.25%), dehydration (14.67%, 18.49%), nausea (13.55%, 13.82%), and vomiting (12.71%, 14.02%) (Fig. S3B and S3C; Table S5). Pharmacovigilance analysis showed a significantly shorter median time-to-onset for afatinib (10.5 days; IQR: 4.00–27.00) compared with other agents. No significant links were found between onset time and event outcomes, age groups, or sex (Fig. S4).

We further employed the Reporting Odds Ratio (ROR) and Bayesian Confidence Propagation Neural Network (BCPNN) to investigate the association between EGFR-TKIs and renal adverse events.^2^, ^3^, ^4^ Table S6 presents the 2 × 2 contingency table, detailed calculation formulas for each disproportionality analysis method, and the predefined positive signal thresholds applied in this study. A signal is considered significant if the lower bounds of the 95% confidence intervals for both ROR and IC exceed 1 and 0, respectively, with a *p*-value <0.05. The analysis revealed that among the five investigated drugs, only prerenal failure demonstrated a significant nephrotoxicity signal across both databases (FAERS database: ROR = 2.96; 95% confidence interval: 1.75–5.01; IC (IC025): 1.49 (0.88); VigiBase database: ROR = 3.02; 95% confidence interval: 1.64–5.57; IC (IC025): 1.53 (0.83)) (Fig. S5; Table S7). Subsequently, we evaluated the signal strength of renal adverse events at the preferred term level for each drug (Fig. S6; Tables S8 and S9). Analysis of the FAERS database revealed that afatinib was associated with an increased risk of prerenal failure, azotemia, and acute kidney injury, whereas erlotinib was associated with an increased risk of proteinuria. In VigiBase, afatinib demonstrated stronger signals with prerenal failure, acute kidney injury, and chromaturia; gefitinib was associated with elevated risks of hematuria and chromaturia. Notably, osimertinib and dacomitinib did not show significant risk signals for any renal adverse events.

To complement the pharmacovigilance findings, we conducted RT-qPCR experiments using human renal proximal tubular (HK-2) cells to assess the expression profiles of renal injury biomarkers and inflammatory mediators following drug exposure. Primer sequences for target genes are provided in Table S10. The results showed that the mRNA expression of neutrophil gelatinase-associated lipocalin (NGAL), angiotensinogen (AGT), secreted phosphoprotein 1 (SPP1), interleukin-1β (IL-1β), and tumor necrosis factor-α (TNF-α) was up-regulated to varying degrees in the drug-treated groups (Fig. 1A–E). However, no significant elevation was observed in kidney injury molecule-1 (KIM1), interleukin-18 (IL-18), interleukin-6 (IL-6), interleukin-10 (IL-10), or interferon-γ (IFN-γ) mRNA levels (Fig. S7A–S7E).

**Figure 1:**
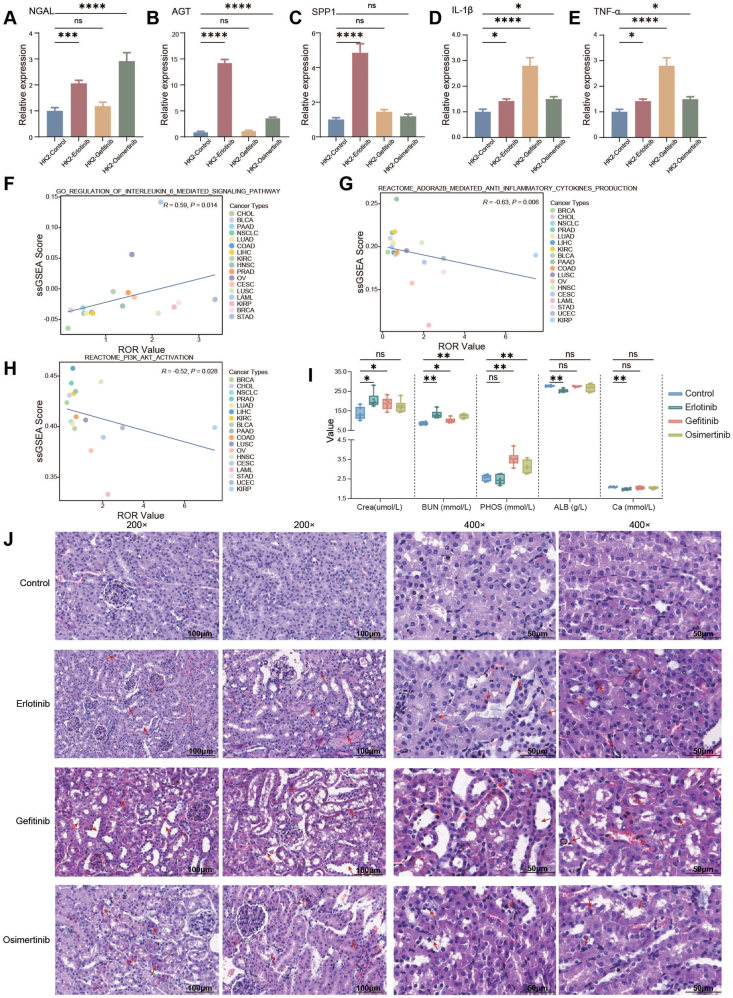
Results of *in vitro* and *in vivo* experiments and assessment of the association between EGFR-TKIs' renal adverse events and biological pathways based on the TCGA database. **(A**–**E)** Bar charts describing the relative expression differences of renal injury markers, including neutrophil gelatinase-associated lipocalin (NGAL), angiotensinogen (AGT), secreted phosphoprotein 1 (SPP1), interleukin-1β (IL-1β), and tumor necrosis factor-α (TNF-α) mRNA, as detected by RT-qPCR after treatment of human renal proximal tubular epithelial cell line (HK-2) with erlotinib, gefitinib, osimertinib, or physiological saline control. The relative expression levels of mRNA were calculated using the 2^–ΔΔCt^ method. ΔCt = Ct (target gene) – Ct (reference gene); ΔΔCt = ΔCt (treatment group) – ΔCt (control group). For relative gene expression, the mean value of the control group was defined as 1 or 100 %. The relative levels of target genes were expressed as fold changes between the control and experimental groups. NS, not significant; ∗*p* < 0.05, ∗∗*p* < 0.01, ∗∗∗*p* < 0.001, and ∗∗∗∗*p* < 0.0001. **(F–H)** Results of Spearman correlation analysis between EGFR-TKIs renal adverse reaction ROR values and single-sample Gene Set Enrichment Analysis (ssGSEA) enrichment scores for processes regulating IL-6 mediated signaling pathways, adenosine A2B (Adora2b) receptor-mediated anti-inflammatory cytokine production, phosphoinositide 3-kinase/protein kinase B (PI3K/AKT) activation. The enrichment scores for these biological pathways in each cancer sample were calculated using ssGSEA, based on Gene Ontology (GO), Kyoto Encyclopedia of Genes and Genomes (KEGG) pathway, and Reactome pathway data from the Molecular Signatures Database (MSigDB). Correlation analysis used Spearman's rank correlation coefficient. **(I)** Box plots comparing the differences in serum creatinine (SCr), blood urea nitrogen (BUN), inorganic phosphorus (P), albumin (ALB), and serum calcium (Ca^2+^), between mice administered gefitinib, erlotinib, or osimertinib, and control mice. Kruskal–Wallis test was used; NS, not significant; ∗*p* < 0.05, ∗∗*p* < 0.01, ∗∗∗*p* < 0.001, and ∗∗∗∗*p* < 0.0001. **(J)** Representative images of hematoxylin-eosin staining of kidney tissue from each group of mice. The arrows indicate sites of injury, such as tubular dilation, flattening of tubular epithelial cells, necrosis and detachment of tubular epithelial cells, vacuolar degeneration of epithelial cells, interstitial edema, and inflammatory cell infiltration. Scale bars: 200 × : 100 μm; 400 × : 50 μm. The present study utilized C57BL/6 mice (6 weeks old; body weight: 25 ± 5 g). Mice were randomly divided into four experimental groups (*n* = 6 per group): control, erlotinib, gefitinib, and osimertinib, with treatments administered via intraperitoneal injection. The control group received intraperitoneal injections of an equal volume of drug vehicle solution; the erlotinib group received intraperitoneal injections of erlotinib (product number: GC10627, 19.5 mg/kg body weight); the gefitinib group received intraperitoneal injections of gefitinib (product number: GC16737, 32.5 mg/kg body weight); and the osimertinib group received intraperitoneal injections of osimertinib (product number: GC16308, 10.4 mg/kg body weight).

To investigate the potential mechanisms of EGFR-TKI-induced nephrotoxicity, this study analyzed transcriptomic data from 18 cancer types in TCGA that matched the drug-adverse event pairs identified in public databases.^5^ The results demonstrated that at the pan-cancer level, the RORs for renal adverse events showed a significant positive correlation with the expression levels of “GO_REGULATION_OF_INTERLEUKIN_6_MEDIATED_SIGNALING_PATHWAY” and significantly negatively correlated with multiple pathways, such as “REACTOME_ADORA2B_MEDIATED_ANTI_INFLAMMATORY_CYTOKINES_PRODUCTION” and “REACTOME_PI3K_AKT_ACTIVATION” (Fig. 1F–H; Fig. S7F–S7M).

To further investigate these nephrotoxic effects, we established mouse models treated with EGFR-TKIs. We performed comparative analysis of blood biochemical parameters and collected renal tissues for bulk transcriptome sequencing (bulk RNA sequencing) and hematoxylin-eosin staining (approved by the Animal Ethics Committee of Zhujiang Hospital, Southern Medical University, LAEC-2024-022). Compared with the control group, the serum creatinine (SCr) and blood urea nitrogen (BUN) of the experimental group mice showed an upward trend after administration, while the inorganic phosphate (P), serum calcium (Ca^2+^), and albumin (ALB) showed varying expression in different groups (Fig. 1I; Table S11). Histopathological examination revealed significant renal tissue damage in the treated groups, including varying degrees of tubular dilation, flattening of tubular epithelial cells, epithelial necrosis, vacuolar degeneration, interstitial edema, and inflammatory cell infiltration (Fig. 1J). Gene Set Variation Analysis (GSVA) of the murine gene expression data revealed significant down-regulation of phosphoinositide 3-kinase (PI3K)–related pathways and pathways involved in the transmembrane transport of substances in the treated groups (Fig. S8). Notably, no significantly altered pathways were observed between the erlotinib-treated and control groups.

However, this study has several important limitations. Due to the inherent underreporting bias in spontaneous reporting systems like FAERS and VigiBase, the true incidence of nephrotoxicity may be significantly underestimated. Secondly, these databases are more likely to capture severe adverse reactions, new drug-related events, or reactions associated with high-profile drugs; selective reporting could distort signal intensity. The lack of detailed clinical context limits the interpretation of results, making it difficult to exclude the interference of confounding factors with nephrotoxicity signals and to analyze risk differences across clinical subgroups accurately. Signal detection methods (ROR/BCPNN) can only suggest statistical associations without adequately controlling for confounding effects from polypharmacy and therefore cannot be used for causal inference; species-specific differences and limited sample sizes constrain preclinical models (cell lines/mice); lastly, this study's analysis of potential molecular pathways has not been validated across multiple dimensions, thereby hindering comprehensive interpretation of the biological basis of drug nephrotoxicity.

In summary, this study systematically characterizes the risk profile and underlying molecular mechanisms of nephrotoxicity in cancer patients receiving EGFR-TKI therapy. The results indicate that, for patients showing signs of nephrotoxicity, it is essential to promptly reduce the dose of EGFR-TKIs or temporarily halt the medication. It is recommended that regular renal function should be incorporated into routine follow-up for patients on EGFR-TKI treatment, with the first 2 months being especially critical. Additionally, exploring validated early biomarkers like SPP1 and NGAL enables early detection of subclinical nephrotoxicity, providing more time for clinical intervention and preventing progression to overt renal dysfunction. It is important to note that establishing a causal link between EGFR-TKIs and nephrotoxicity requires integrated multi-omics data analysis, and developing precise prevention and control strategies must be validated through multicenter prospective cohort studies.

## CRediT authorship contribution statement

**Huili Xiao:** Writing – review & editing, Writing – original draft, Visualization, Validation, Software, Methodology, Investigation, Formal analysis, Data curation, Conceptualization. **Anqi Lin:** Writing – review & editing, Visualization, Software, Formal analysis. **Wentao Xu:** Visualization, Investigation, Formal analysis. **Junyi Shen:** Software, Investigation. **Cuiying Chen:** Visualization, Investigation. **Jian Zhang:** Supervision, Resources, Conceptualization. **Ting Wei:** Supervision, Resources, Conceptualization. **Peng Luo:** Writing – review & editing, Supervision, Resources, Conceptualization.

## Ethics declaration

This study strictly complied with relevant international and domestic ethical guidelines and legal regulations and was approved by the Ethics Committee of Zhujiang Hospital, Southern Medical University (ID: LAEC-2024-022).

## Funding

This work was supported by the National Natural Science Foundation of China (Grant No. 82172750).

## Conflict of interests

The authors declared that the research was conducted in the absence of any commercial or financial relationships that could be construed as a potential conflict of interest.

## Figures and Tables

**Figure S1:**
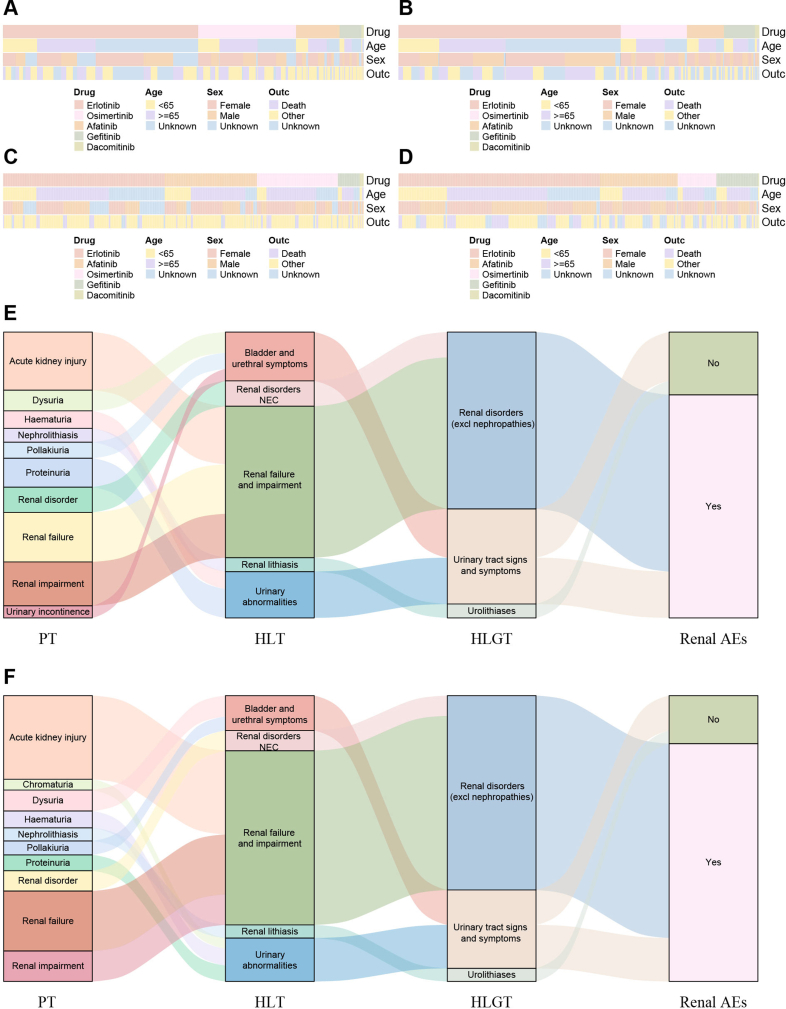
Characteristics of the population included in the analysis and hierarchical relationships of preferred terms (PTs) for EGFR-TKI treatment-related renal adverse events. **(A, B)** Heatmaps showing the characteristic classification of populations with EGFR-TKIs as the primary suspected drug based on treatment, age, gender, and outcome in the FAERS (A) and VigiBase (B) databases. **(C, D)** Heatmaps showing the characteristic classification of populations experiencing renal adverse events based on treatment drug, age, gender, and outcome in the FAERS (C) and VigiBase (D) databases. **(E, F)** Sankey diagrams showing the hierarchical relationships of PTs for renal adverse events in the FAERS (E) and VigiBase (F) databases.

**Figure S2:**
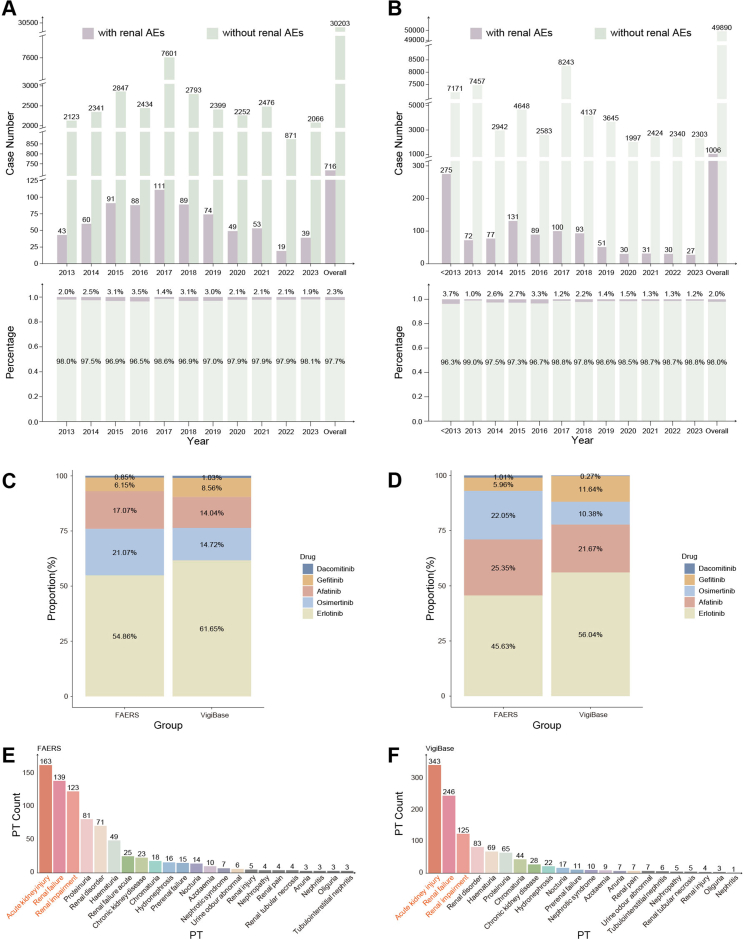
Statistical analysis of EGFR-TKI treatment-related adverse event reports from the FAERS database (2013–2023) and VigiBase database (all years through the end of 2023). **(A)** The upper bar graph shows the number of EGFR-TKIs reports with renal adverse reactions, without renal adverse reactions, and the overall situation in the FAERS database from 2013 to 2023. The lower proportion bar graph displays the percentage of EGFR-TKI reports with renal adverse events versus those without renal adverse events in the FAERS database during the 2013–2023 period. **(B)** The upper bar graph shows the number of EGFR-TKI reports with renal adverse reactions, without renal adverse reactions, and the overall situation in the VigiBase database for all years. The lower proportion bar graph displays the percentage of EGFR-TKI reports with renal adverse events versus those without renal adverse events in the VigiBase database for all years. **(C, D)** Stacked bar charts describe the proportion of all adverse events (C) and renal-related adverse events (D) for each EGFR-TKI drug in the FAERS and VigiBase databases. **(E, F)** Bar charts describe the distribution of EGFR-TKI treatment-related renal adverse events in the FAERS (E) and VigiBase (F) databases.

**Figure S3:**
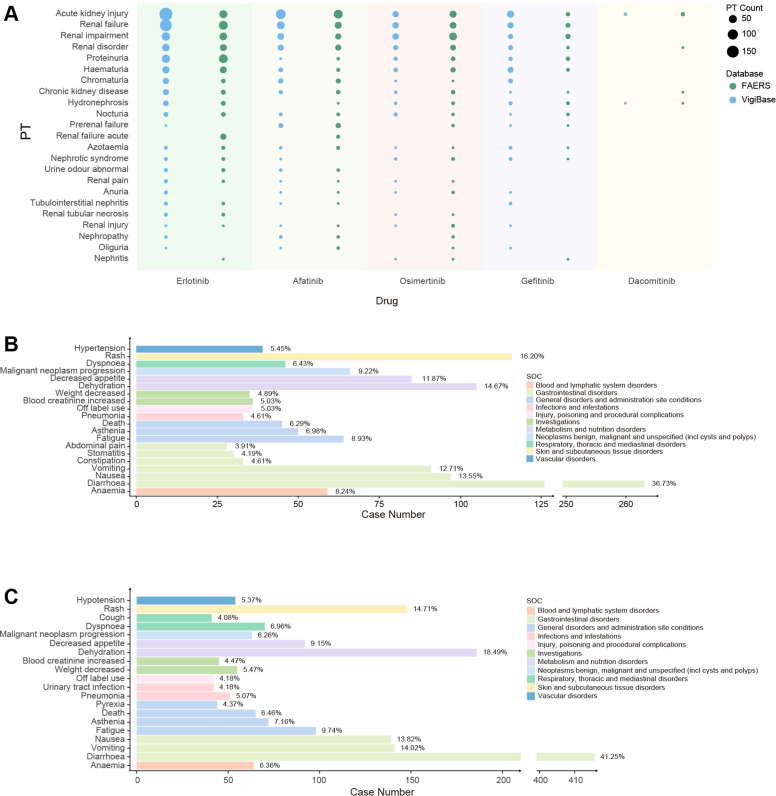
Distribution of EGFR-TKI-associated renal adverse events and characteristics of co-occurring adverse events in FAERS and VigiBase databases. **(A)** Bubble chart showing the specific situations of different EGFR-TKI-related renal adverse events in the FAERS and VigiBase databases. **(B)** Bar chart showing the statistical values of the top 20 preferred terms (PTs) co-reported with adverse events in the FAERS database. **(C)** Bar chart showing the statistical values of the top 20 PTs co-reported with adverse events in the VigiBase database.

**Figure S4:**
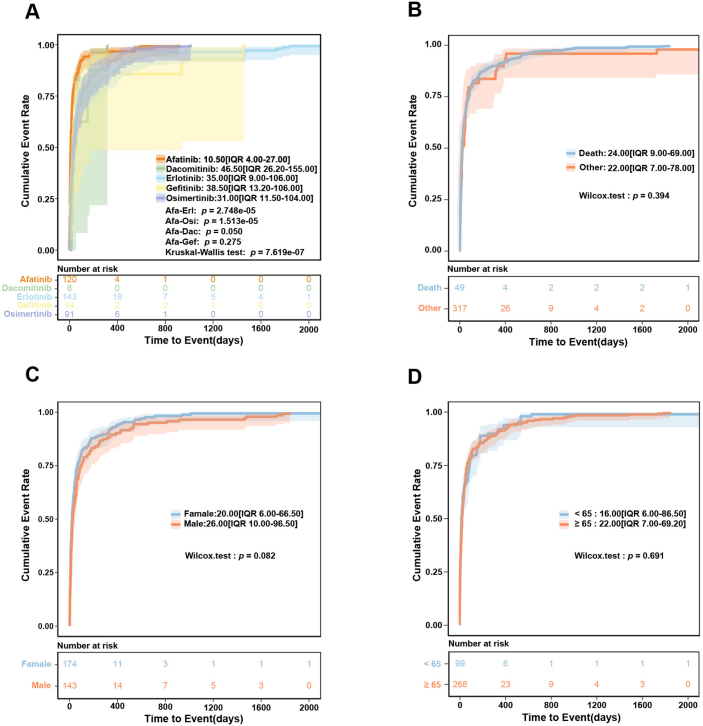
Cumulative probability curves of EGFR-TKI treatment-related renal adverse reaction events. **(A)** Cumulative curves and between-group difference analysis of the time to occurrence of EGFR-TKI renal adverse events based on drugs. Statistical testing used the Kruskal–Wallis test. **(B**–**D)** Cumulative curves and between-group difference analysis of the time to occurrence of EGFR-TKI renal adverse events by outcome (B), gender (C), and age group (D). Statistical analysis used the Wilcoxon rank-sum test.

**Figure S5:**
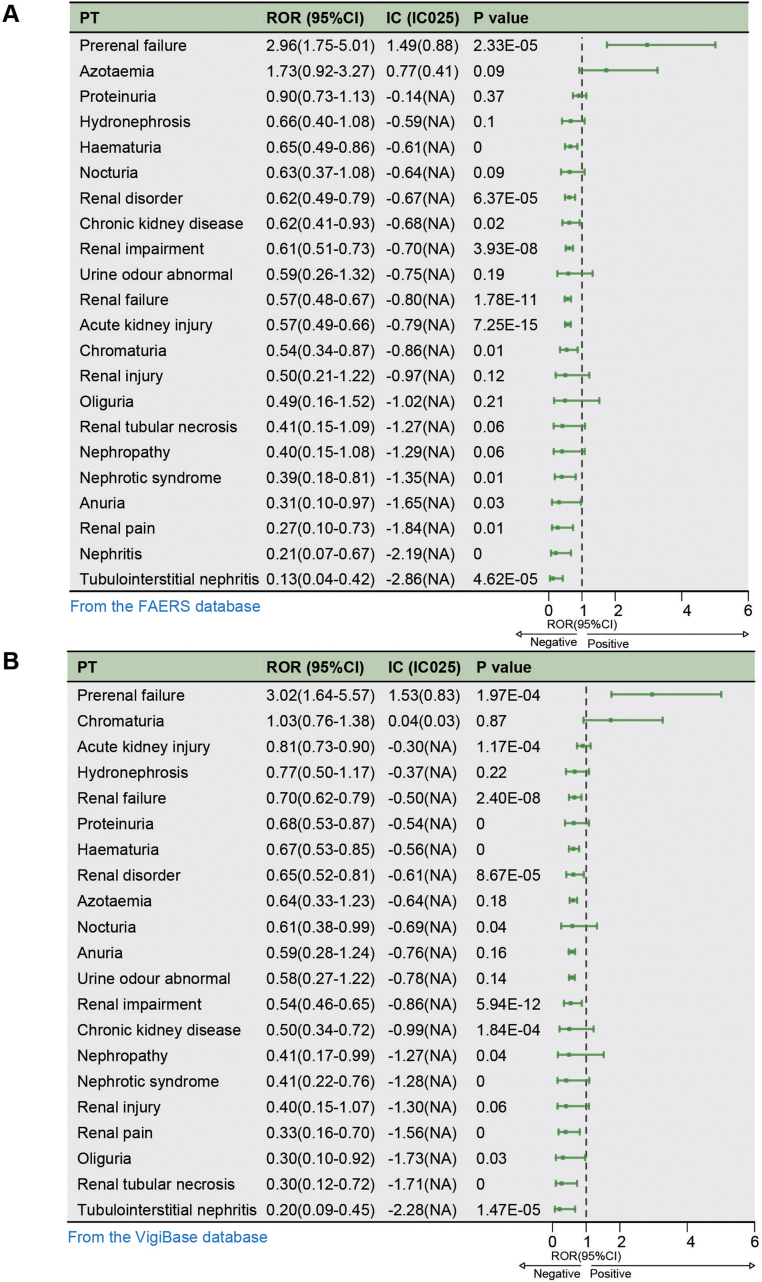
Overall signal intensity of EGFR-TKI drug-related renal adverse reactions. **(A)** Forest plot of overall adverse reaction risk signals for EGFR-TKIs in the FAERS database. **(B)** Forest plot of overall adverse reaction risk signals for EGFR-TKIs in the VigiBase database.

**Figure S6:**
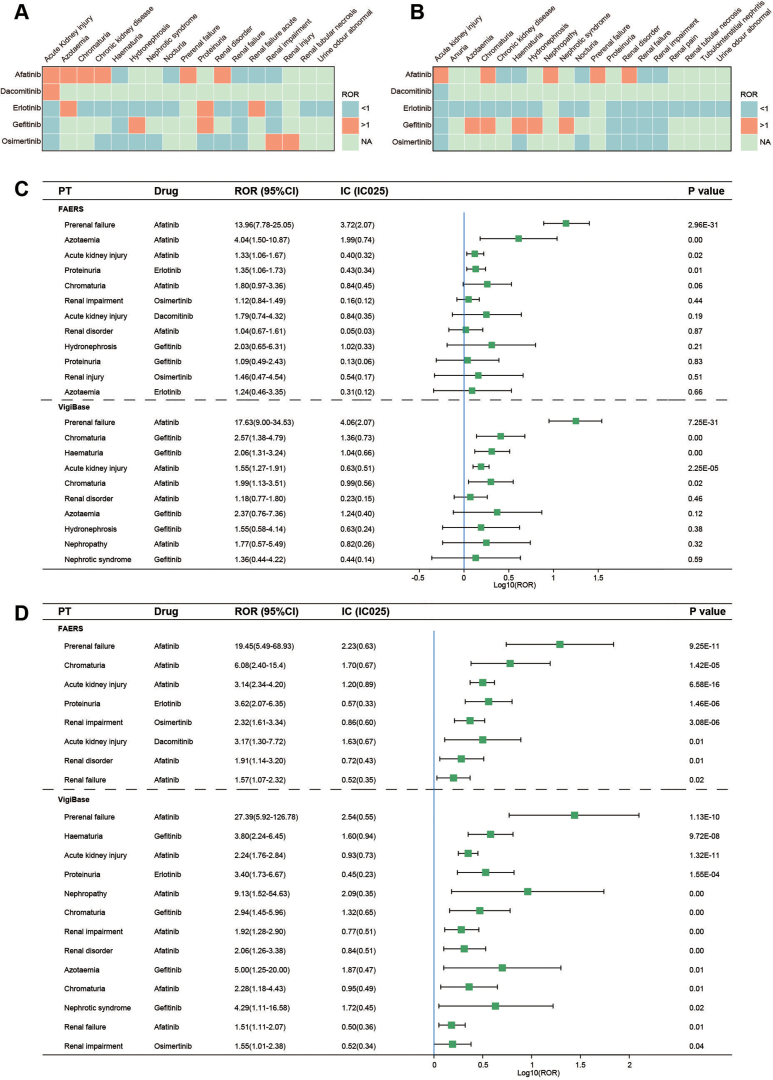
Results of EGFR-TKI renal adverse reaction risk signal mining from FAERS and VigiBase databases. **(A)** Heatmap showing the Reporting Odds Ratio (ROR) values for 17 renal adverse events (with at least 3 cases) under different drug treatment strategies in the FAERS database. **(B)** Heatmap showing the ROR values for 19 renal adverse events (with at least 3 cases) under different drug treatment strategies in the VigiBase database. **(C)** Forest plot of EGFR-TKI renal adverse reaction risk signals from FAERS and VigiBase databases. The ROR is expressed in the form of Log_10_(ROR). **(D)** Forest plot comparing adverse reaction risk signals among five EGFR-TKI drugs. The ROR is expressed in the form of Log_10_(ROR).

**Figure S7:**
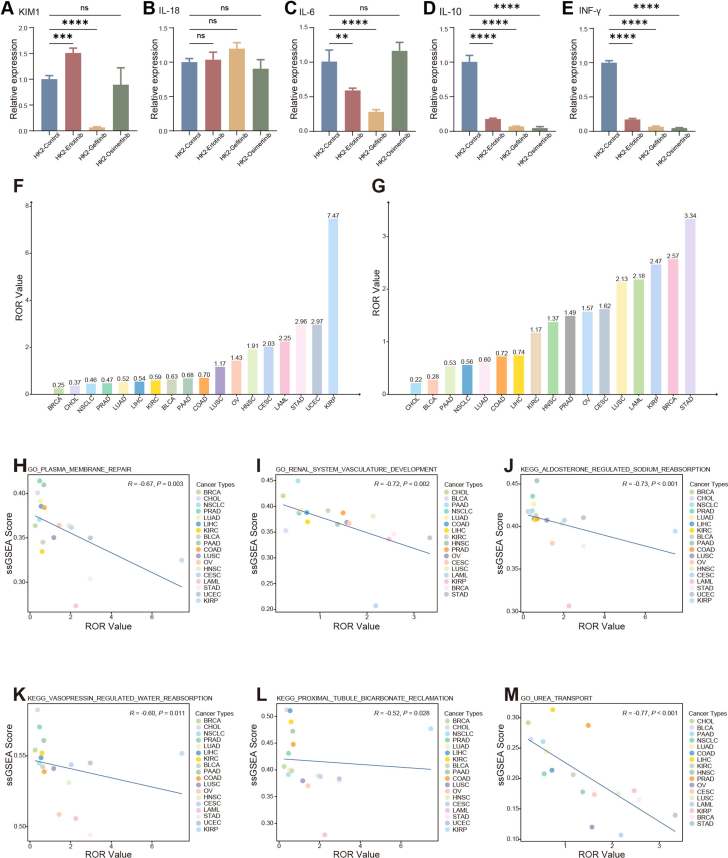
Results of RT-qPCR experiments and assessment of the association between EGFR-TKI renal adverse events and biological pathways based on the TCGA database. **(A**–**E)** Bar charts describing the relative expression differences of kidney injury molecule-1 (KIM1), interleukin-18 (IL-18), interleukin-6 (IL-6), interleukin-10 (IL-10), and interferon-γ (INF-γ) mRNA as detected by RT-qPCR after treatment of human renal proximal tubular epithelial cell line (HK-2) with erlotinib, gefitinib, osimertinib, or physiological saline. NS, not significant; ∗*P* < 0.05, ∗∗*P* < 0.01, ∗∗∗*P* < 0.001, and ∗∗∗∗*P* < 0.0001. **(F)** Bar chart showing the ROR values of EGFR-TKI renal adverse reactions across different cancer types in the FAERS database. **(G)** Bar chart showing the ROR values of EGFR-TKI renal adverse reactions across different cancer types in the VigiBase database. **(H**–**M)** Results of Spearman correlation analysis between EGFR-TKI renal adverse reaction ROR values and single-sample Gene Set Enrichment Analysis (ssGSEA) enrichment scores for cell membrane repair, renal vascular development, aldosterone-regulated sodium reabsorption, antidiuretic hormone-regulated water reabsorption, proximal tubular reabsorption of bicarbonate, and urea transport pathways. The enrichment scores for these biological pathways in each cancer sample were calculated using ssGSEA, based on Gene Ontology (GO), Kyoto Encyclopedia of Genes and Genomes (KEGG) pathway, and Reactome pathway data from the Molecular Signatures Database (MSigDB). Correlation analysis used Spearman's rank correlation coefficient.

**Figure S8:**
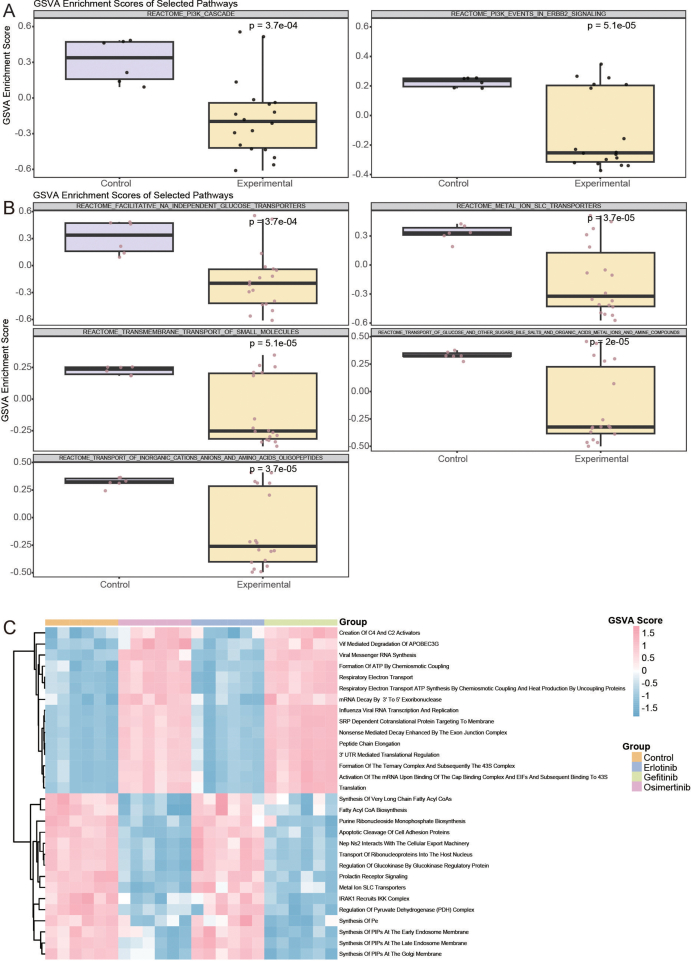
Significant changes in specific pathway activities between the mouse experimental and control groups. **(A, B)** Box plots showing significant down-regulation of PI3K-related pathways and substance transmembrane transport-related pathways in experimental group mice; between-group difference analysis was performed using a *t*-test. **(C)** Heatmap showing significant changes in REACTOME signaling pathway activities in the osimertinib and gefitinib groups compared with the control group.

## Appendix A Supplementary data

Multimedia component 1 Multimedia component 1

Table S1 Clinical characteristic analysis of cancer patients with EGFR-TKIs as the primary suspected drug in the FAERS database (January 2013 to December 2023) Table S1

Table S2 Clinical characteristic analysis of cancer patients with EGFR-TKIs as the primary suspected drug in the VigiBase database (through December 2023) Table S2

Table S3 Clinical characteristics of patients experiencing EGFR-TKI-related renal adverse events in the FAERS database (January 2013 to December 2023). Table S3

Table S4 Clinical characteristics of patients experiencing EGFR-TKI-related renal adverse events in the VigiBase database (through December 2023). Table S4

Table S5 Statistical analysis results of renal adverse events and concurrent adverse events associated with EGFR-TKIs. Table S5

Table S6 The 2 × 2 contingency table, detailed calculation formulas for each disproportionality analysis method, and the predefined positive signal thresholds applied in this study. Table S6

Table S7 Overall adverse reaction risk signals for EGFR-TKIs in the FAERS and VigiBase database. Table S7

Table S8 EGFR-TKIs renal adverse reaction risk signals from FAERS and VigiBase databases. Table S8

Table S9 Comparison of adverse reaction risk signals among five EGFR-TKIs. Table S9

Table S10 Primers used in RT-qPCR experiments in this study. Table S10

Table S11 Serum biochemical test results in mouse models. Table S11
