# Supplementary material for: Renal adverse events in EGFR-TKI treatment: Comprehensive characterization of clinical patterns and molecular underpinnings
Source: Genes Dis. 2025 Nov 28;13(4):101953. doi: 10.1016/j.gendis.2025.101953 (PMC12993402; doi:10.1016/j.gendis.2025.101953)
Supplement: Multimedia component 1 [file mmc1.docx]

**Supplementary Materials**

**Materials and Methods**

**Cell Lines and Cell Viability Assay**

Human renal proximal tubular epithelial cells (HK-2) were cultured in minimum essential medium (MEM) supplemented with 10% fetal bovine serum (FBS). The incubator was strictly maintained at 37°C with 5% CO2. We assessed cellular responses to drugs using the Cell Counting Kit-8 reagent (CCK-8, GK10001, GLPBIO). A total of 10,000 cells/well were seeded into 96-well culture plates and incubated. The following day, complete medium without drugs was added to establish the control group, while complete medium supplemented with serial dilutions of drugs n was added to establish the experimental groups. This study included one control group and three experimental groups (osimertinib, erlotinib, and gefitinib groups, respectively). The plates were then placed in an incubator for 48 hours. On the third day, the original medium was replaced with MEM containing 10% CCK-8 solution, and the plates were placed in the incubator for 1 to 4 hours. The absorbance of each well was measured at a wavelength of 450 nm using a microplate reader. After normalizing the values to those of the untreated group, the half maximal inhibitory concentration (IC_50_) was calculated.

**Quantitative Real-Time Polymerase Chain Reaction (RT-qPCR)**

Cells were collected, and RNA was extracted using TRIzol reagent followed by phase separation with chloroform and isopropanol precipitation. The samples were then washed with 75% ethanol and resuspended in diethyl pyrocarbonate (DEPC)-treated water. Prior to downstream processing, the concentration and quality of RNA were determined using a NanoDrop 2000 spectrophotometer. Subsequently, reverse transcription was performed using the EZB® Color All-in-one Reverse Transcription Kit (with DNase) (EZBioscience, RT3C), and the product was diluted with DEPC-treated water. RNA levels were quantified using the EZB® 2X EZ Color SYBR Green qPCR Master Mix kit (EZBioscience, CQ22). Glyceraldehyde-3-phosphate dehydrogenase (GAPDH) was used as an internal reference. The relative expression levels of mRNA were calculated using the 2^-ΔΔCt method. ΔCt = Ct(target gene) - Ct(reference gene), ΔΔCt = ΔCt(treatment group) - ΔCt(control group). For relative gene expression, the mean value of the control group was defined as 1 or 100%. The relative levels of target genes were expressed as fold changes between the control and experimental groups. The primer sequences for target genes are shown in Supplementary Table 10.

**Establishment of EGFR-TKIs-Treated Mouse Models**

The present study utilized C57BL/6 mice (6-week-old, body weight 25 ± 5 g) purchased from Zhuhai Beststudy Biotechnology Co., Ltd. (Zhuhai, China). All animals were housed in the Experimental Animal Center of Zhujiang Hospital, Southern Medical University under standardized conditions. Mice were randomly assigned and housed in specific pathogen-free, temperature-controlled (22 ± 1°C) animal facilities with a 12-hour light/dark cycle and ad libitum access to water and food. Mice were randomly divided into four experimental groups (n=6 per group): control, erlotinib, gefitinib, and osimertinib, with treatments administered via intraperitoneal injection. The control group received intraperitoneal injections of an equal volume of drug vehicle solution; the erlotinib group received intraperitoneal injections of erlotinib (product number: GC10627, 19.5 mg/kg body weight); the gefitinib group received intraperitoneal injections of gefitinib (product number: GC16737, 32.5 mg/kg body weight); and the osimertinib group received intraperitoneal injections of osimertinib (product number: GC16308, 10.4 mg/kg body weight). Following a 7-day acclimation period, mice were treated with the respective drugs for 10 consecutive days and subsequently euthanized 3 days after the final dose. At the conclusion of the experiment, blood samples were collected via rapid cardiac puncture, followed by kidney excision for further analyses. Half of each mouse kidney was transversely sectioned and fixed in paraffin embedding for histological analysis. The remaining half of each kidney tissue was preserved for high-throughput bulk transcriptome sequencing (bulk RNA-seq) analysis. All animal procedures were approved by the Animal Experimental Ethics Committee of Zhujiang Hospital, Southern Medical University (approval number: LAEC-2024-022).

**Mouse Blood Biochemical Analysis**

Blood samples were centrifuged at 4°C and 3000 rpm for 15 minutes to isolate serum. The isolated serum was subsequently sent to Topo Bioengineering Co., Ltd. for quantitative measurements of renal function biomarkers, including the serum creatinine (SCr), blood urea nitrogen (BUN), albumin (ALB), serum calcium (Ca^2+^), and inorganic phosphate (P).

**Hematoxylin and Eosin (H&E) Staining**

Kidneys were promptly dissected and fixed overnight in formalin solution at 4°C. Kidneys were embedded in paraffin and sectioned to a thickness of 4 μm. The sections were first deparaffinized with water, then placed in three different fresh xylene containers for 10 minutes, followed by gradient ethanol chambers (100%, 95%, 85%, and 75%) for 5 minutes each, and finally rinsed with running water for 3 minutes. Sections were stained with hematoxylin solution for 10 minutes and subsequently washed with running water for 3 minutes. The sections were then stained with eosin solution at room temperature for 1 minute, followed by an additional 3-minute wash with running water. Finally, the sections were dehydrated in 75%, 85%, 95%, and 100% ethanol for 1 minute each, and then washed in xylene 3 times for 5 minutes each. After sealing with neutral resin and drying, the sections were observed under a microscope, then photographed and analyzed using a 3D HISTECH scanner.

**TCGA Pathway Enrichment and Correlation Studies**

This study utilized the UCSC Xena platform to obtain transcriptome data (quantified as fragments per kilobase of exon per million mapped reads [FPKM]) of 18 cancer types from TCGA project, which matched the screening results from public adverse drug reaction databases. These data were subsequently converted to transcripts per million (TPM) values. Subsequently, based on Gene Ontology (GO), Kyoto Encyclopedia of Genes and Genomes (KEGG) pathways, and Reactome pathway data from the Molecular Signatures Database (MSigDB), single-sample Gene Set Enrichment Analysis (ssGSEA) was employed to calculate the enrichment scores of these biological pathways in each cancer sample, with median values being used for subsequent analysis. Previous research has shown that the enrichment score of a specific gene set reflects the activity level of the biological process represented by that gene set in the sample, thus reflecting the co-expression pattern of gene set members. We conducted correlation analyses between the ROR of EGFR-TKIs-related renal adverse events across cancer types and the median ssGSEA scores of various biological pathways to explore potential signaling pathways associated with EGFR-TKIs-induced renal adverse events.

**Transcriptome Sequencing and Pathway Analysis of Mouse Model Kidney Tissue**

The additional portion of kidney tissue obtained from dissection was rinsed with pre-cooled phosphate-buffered saline (PBS) to remove blood, immediately flash-frozen in liquid nitrogen, and subsequently transferred to a -80°C freezer for storage. Total RNA was extracted from the frozen kidney tissue samples using TRIzol™ Reagent. Following the kit instructions, tissues were ground with liquid nitrogen, cell lysis buffer was added for thorough cell lysis, and following centrifugation, washing, and other procedural steps, total RNA was isolated. The purity of RNA was subsequently determined using a Nano Drop spectrophotometer, RNA concentration was measured using the Qubit RNA BR (Broad-Range) Assay Kit, and RNA integrity was assessed using RNA Screen Tape. Based on the total amount of RNA available, 0.1-1μg of total RNA was utilized, mRNA was isolated using the Aibotek Poly(A)mRNA Capture Module (catalog number: RK20340) kit, and subsequently the mRNA library was constructed using the Aibotek Fast RNA-seq Lib Prep Kit V2 (catalog number: RK20306). Library concentration was determined using the Qubit™ dsDNA HS Assay Kit, library fragment distribution was analyzed using D1000 Screen Tape, and ultimately, library molar concentration was precisely measured using the KAPA Library Quant kit (Illumina) universal qPCR Mix. The library was subsequently sequenced using the NovaSeq xplus instrument in conjunction with the accompanying NovaSeq X Series 25B Reagent Kit (300 Cycle). Sequencing data were aligned to the mouse reference genome (mm39) using HISAT2 software, and quantitative analysis of gene expression was performed using htseq-count to generate raw count matrices for downstream analysis. Gene expression levels were quantified as FPKM. The GSVA package in R software (version 4.4.0) was used to calculate gene set enrichment scores for each sample, and pathways exhibiting significant enrichment differences between experimental and control groups were identified through differential analysis using the limma package.

**Statistical Analysis**

All statistical analyses and data visualizations in this study were performed using R software (https://www.r-project.org/; version 4.4.0), GraphPad Prism (version 9.5.0), and Microsoft Excel (2021). Differences in adverse event occurrence time between two groups and among multiple groups were analyzed using the Wilcoxon test and Kruskal-Wallis test, respectively, with adverse reaction occurrence time statistically described using median and interquartile range (IQR). Differences between control and experimental groups in RT-qPCR cell experiments were analyzed using one-way analysis of variance (ANOVA). Spearman rank correlation coefficients were used to analyze the correlation between RORs of EGFR-TKIs treatment-related renal adverse events at the pan-cancer level and biological pathway enrichment scores. The difference analysis of biochemical indicators in mouse serum samples between the control group and the experimental groups was performed using the Kruskal-Wallis test. For mouse model gene expression results, the Gene Set Variation Analysis (GSVA) algorithm was used to calculate pathway activity scores for each sample, and the Limma package was used for intergroup differential analysis. P values < 0.05 were considered statistically significant, and all statistical tests were two-sided.

**Result**

Drug concentration in RT-qPCR:


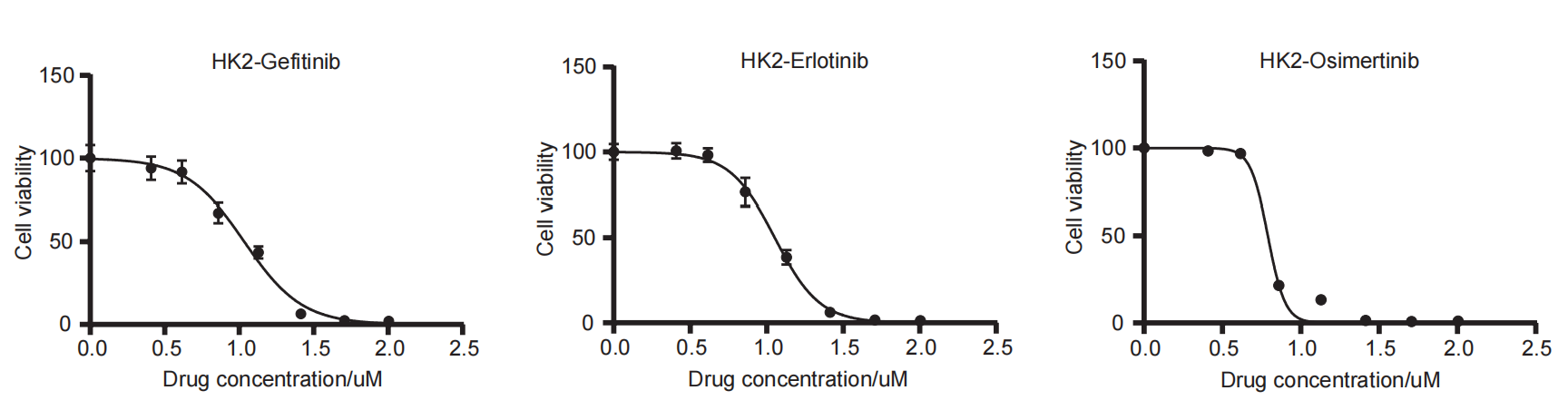


Preliminary studies have verified that the IC_50_ values for gefitinib, erlotinib, and osimertinib in HK-2 cells are 9.83 μM, 10.3 μM, and 5.1 μM, respectively.

**Discussion**

Based on TCGA database and mouse renal transcriptome analysis, this study reveals that EGFR-TKI-related renal AEs may involve multiple coordinated regulatory mechanisms, including IL-6 signaling pathway activation, impaired production of anti-inflammatory cytokines mediated by Adora2b receptors, downregulation of PI3K-related signaling pathways, cell membrane repair defects, abnormal renal vascular development, aldosterone-regulated sodium reabsorption, antidiuretic hormone-regulated water reabsorption, proximal tubular reabsorption of bicarbonate, urea transport, and dysfunction of transmembrane transport of substances such as glucose, amino acids, and ions.

As a key pro-inflammatory factor, IL-6 is synthesized and secreted by proximal tubular epithelial cells (TECs) under hypoxic or inflammatory stimuli, subsequently activating downstream Janus Kinase/Signal Transducer and Activator of Transcription 3 (JAK/STAT3) pathways through receptor binding, driving inflammatory cascades, and participating in various disease processes (1,2). In a study utilizing rat models, researchers demonstrated that Mahuang Fuzi and Shenzhuo Decoction (MFSD) improved glomerular ultrastructural lesions by inhibiting the interleukin-6/Signal Transducer and Activator of Transcription 3 (IL-6/STAT3) pathway, restoring podocyte marker protein expression, and inhibiting cell apoptosis (3). Hyperactivation of this pathway may potentially exacerbate EGFR-TKI-related kidney injury. Multiple studies have demonstrated that the Adora2b adenosine receptor can exert significant anti-inflammatory effects by activating its signaling pathway, and Adora2b signal transduction is specifically associated with promoting regulatory T cells and facilitating the resolution of inflammation (4). A preclinical investigation employing mouse models revealed that endothelial Adora2b signaling can alleviate kidney injury by inhibiting vascular endothelial growth factor (VEGF) production during diabetic nephropathy, suggesting that targeted regulation of Adora2b signaling may provide novel therapeutic strategies for treating various kidney diseases (including DIKD) (5).

The PI3K/AKT signaling pathway plays a crucial regulatory role in kidney diseases, with extensive studies demonstrating that this pathway ameliorates kidney injury by inhibiting signaling pathways related to inflammation, oxidative stress, apoptosis, epithelial-mesenchymal transition (EMT), and autophagy (6). For instance, Troxerutin enhances microtubule-associated protein 4 (MAP4) expression via the PI3K/AKT pathway, thereby reducing oxidative stress (OS) and inflammatory responses, inhibiting apoptosis, and attenuating kidney injury; similarly, the heparin-binding site-deficient fibroblast growth factor 1 mutant (FGF1ΔHBS)ameliorates CKD through OS and inflammation inhibition mediated by this pathway (7,8). PI3K/AKT activation alleviates AKI after liver transplantation by inducing FoxO3a nuclear export and deacetylation; additionally, Arbutin attenuates lipopolysaccharide (LPS) -induced AKI by inhibiting inflammation and apoptosis through the PI3K/AKT/Nrf2 pathway; furthermore, Nicorandil mitigates cisplatin-induced rat AKI by activating the phosphatidylinositol 3-kinase/Protein kinase B/mechanistic target of rapamycin(PI3K/AKT/mTOR) signaling cascade and inhibiting autophagy (9–12). Emerging research has revealed potential associations between Adora2b signaling and the PI3K/AKT pathway, wherein Adora2b signals can phosphorylate AKT (specifically at the Ser473 site) via cyclic adenosine monophosphate/protein kinase A (cAMP/PKA) cascades. In experimental penile erection models, Adora2b activates endothelial nitric oxide synthase (eNOS) via the PI3K/AKT pathway, subsequently promoting nitric oxide (NO) generation (13). Reduction of adenosine in wild-type mice or deletion of the Adora2b receptor gene in mutant mice significantly attenuated PI3K/AKT activation and eNOS phosphorylation (13). Based on the potential renoprotective effects of Adora2b signaling and PI3K/AKT signaling pathways, targeted activation of the Adora2b-PI3K/AKT signaling axis may synergistically mitigate drug-induced kidney injury through multiple mechanistic pathways, while conversely, pathway inhibition may cause or exacerbate the risk of renal AEs.

Maintenance of renal homeostasis depends on biological processes such as cell membrane repair, vascular development, and the transport of various substances (14,15). The key molecule for renal cell membrane repair, MG53 (TRIM72), ensures renal structural integrity through dynamic repair mechanisms and participates in regulating inflammatory responses during kidney injury. Additionally, aldosterone and antidiuretic hormone precisely regulate sodium and water reabsorption, respectively, coordinating with renal tubular transport of bicarbonate and urea to maintain the homeostasis of the internal environment (14,15). Normal renal vascular development depends on the regulation of VEGF. However, although VEGF inhibitors (such as bevacizumab) can suppress pathological angiogenesis, they may directly damage the glomeruli, leading to proteinuria, hypertension, and other adverse conditions (16). Aldosterone, as a core member of the mineralocorticoid family, dominates renal Na^+^ reabsorption by regulating the transcription and activity of epithelial sodium channels (ENaC), thereby playing a key role in fluid balance and blood pressure regulation (17). Imbalance in aldosterone's physiological function is not only closely related to systemic diseases such as hypertension but also directly threatens renal homeostasis (17). Weakened aldosterone-mediated sodium reabsorption can lead to hyponatremia, hyperkalemia, and ineffective blood volume, resulting in reduced renal perfusion and subsequently inducing AKI. Concurrently, defects in aldosterone's regulation of acid-base balance may accelerate the progression of renal insufficiency (18,19).

Antidiuretic hormone (ADH, also known as vasopressin or arginine vasopressin) is primarily synthesized in the hypothalamus and stored in the posterior pituitary, serving as a critical hormone in maintaining fluid osmotic balance, blood pressure regulation, and kidney function (20–23). On one hand, ADH binds to V2 receptors in the renal collecting duct, thereby enhancing aquaporin activity to promote water reabsorption, concentrate urine, and reduce urine volume; on the other hand, through activating ENaC and urea transporters, it synergistically increases the reabsorption of sodium and urea, establishing a high osmotic gradient in the medulla to maintain urine concentrating capacity (20). If ADH function is inhibited, this disruption will lead to impaired water reabsorption, resulting in polyuria, hypernatremia, and hemoconcentration, while secondary volume depletion can reduce renal perfusion, thereby exacerbating the risk of AKI (22).

Reabsorption of bicarbonate (HCO_3_^-^) by the renal proximal tubules represents a key step in maintaining acid-base balance in the body. This process is collaboratively mediated by the Na⁺/H⁺ exchanger (NHE3) and carbonic anhydrase (CA) located on the tubular apical membrane. When this process is inhibited, the reduced HCO_3_^-^ reabsorption efficiency can lead to proximal renal tubular acidosis, resulting in electrolyte disorders and renal function impairment (24,25). The urea transport system plays a key role in maintaining nitrogen balance and osmotic regulation—through the medullary urea cycling mediated by specific urea transporters (UTs), which collectively play a decisive role in the inner medullary urine concentration mechanism (26–28). Abnormal urea transport is associated with azotemia, nephrogenic diabetes insipidus, renal tubular concentration dysfunction, and various pathological conditions (26–29). Urea transporter B (UT-B) gene knockout mice exhibited severe renal dysfunction and structural damage in long-term observations, with renal medullary atrophy likely resulting from severe polyuria and hydronephrosis (30). As the principal organ for excretion and homeostatic regulation, normal kidney function highly depends on the precise regulation of substance transport networks for glucose, amino acids, ions, and small molecule metabolites. Dysfunction of these transport systems is closely associated with renal tubular acidosis, diabetic nephropathy, CKD, AKI, and various other pathological states (31–39), indicating that the integrity of renal tubular substance transport pathways constitutes an important molecular basis for maintaining renal physiological functions.

In summary, the pathological mechanisms of EGFR-TKI-related nephrotoxicity involve abnormalities in multiple coordinately regulated molecular networks. Clinical monitoring strategies should focus on fluctuations in urine output, dynamic changes in blood pressure, and abnormal electrolyte levels (such as hypernatremia/hyperkalemia), while maintaining vigilance regarding the secondary effects of non-renal symptoms (such as diarrhea and dehydration) on renal perfusion. Targeted regulation of signaling pathways such as IL-6, Adora2b, and PI3K/AKT may represent promising therapeutic targets for mitigating EGFR-TKIs nephrotoxicity.

**References**

1. Mihara M, Hashizume M, Yoshida H, Suzuki M, Shiina M. IL-6/IL-6 receptor system and its role in physiological and pathological conditions. Clin Sci Lond Engl 1979. 2012 Feb;122(4):143–59.

2. Wang J, Xiong M, Fan Y, Liu C, Wang Q, Yang D, et al. Mecp2 protects kidney from ischemia-reperfusion injury through transcriptional repressing IL-6/STAT3 signaling. Theranostics. 2022;12(8):3896–910.

3. Zhao Q, Dai H, Jiang H, Zhang N, Hou F, Zheng Y, et al. Activation of the IL-6/STAT3 pathway contributes to the pathogenesis of membranous nephropathy and is a target for Mahuang Fuzi and Shenzhuo Decoction (MFSD) to repair podocyte damage. Biomed Pharmacother Biomedecine Pharmacother. 2024 May;174:116583.

4. Ehrentraut H, Westrich JA, Eltzschig HK, Clambey ET. Adora2b adenosine receptor engagement enhances regulatory T cell abundance during endotoxin-induced pulmonary inflammation. PloS One. 2012;7(2):e32416.

5. Tak E, Ridyard D, Kim JH, Zimmerman M, Werner T, Wang XX, et al. CD73-dependent generation of adenosine and endothelial Adora2b signaling attenuate diabetic nephropathy. J Am Soc Nephrol JASN. 2014 Mar;25(3):547–63.

6. Wang H, Gao L, Zhao C, Fang F, Liu J, Wang Z, et al. The role of PI3K/Akt signaling pathway in chronic kidney disease. Int Urol Nephrol. 2024 Aug;56(8):2623–33.

7. Guan T, Zheng Y, Jin S, Wang S, Hu M, Liu X, et al. Troxerutin alleviates kidney injury in rats via PI3K/AKT pathway by enhancing MAP4 expression. Food Nutr Res. 2022;66.

8. Wang D, Jin M, Zhao X, Zhao T, Lin W, He Z, et al. Correction: FGF1ΔHBS ameliorates chronic kidney disease via PI3K/AKT mediated suppression of oxidative stress and inflammation. Cell Death Dis. 2023 Apr 19;14(4):277.

9. Meng F, Zhang Z, Chen C, Liu Y, Yuan D, Hei Z, et al. PI3K/AKT activation attenuates acute kidney injury following liver transplantation by inducing FoxO3a nuclear export and deacetylation. Life Sci. 2021 May 1;272:119119.

10. Zhang B, Zeng M, Li B, Kan Y, Wang S, Cao B, et al. Arbutin attenuates LPS-induced acute kidney injury by inhibiting inflammation and apoptosis via the PI3K/Akt/Nrf2 pathway. Phytomedicine Int J Phytother Phytopharm. 2021 Feb;82:153466.

11. Fahmy MI, Khalaf SS, Yassen NN, Sayed RH. Nicorandil attenuates cisplatin-induced acute kidney injury in rats via activation of PI3K/AKT/mTOR signaling cascade and inhibition of autophagy. Int Immunopharmacol. 2024 Jan 25;127:111457.

12. Asgaritarghi G, Farsani SSM, Sadeghizadeh D, Najaﬁ F, Sadeghizadeh M. Anti-Cancer Role of Dendrosomal Nano Solanine in Chronic Myelogenous Leukemia Cell Line through Attenuation of PI3K/AKT/mTOR Signaling Pathway and Inhibition of hTERT Expression. Curr Mol Pharmacol. 2023 Mar 27;16(5):592–608.

13. Wen J, Grenz A, Zhang Y, Dai Y, Kellems RE, Blackburn MR, et al. A2B adenosine receptor contributes to penile erection via PI3K/AKT signaling cascade-mediated eNOS activation. FASEB J Off Publ Fed Am Soc Exp Biol. 2011 Aug;25(8):2823–30.

14. Cai C, Masumiya H, Weisleder N, Matsuda N, Nishi M, Hwang M, et al. MG53 nucleates assembly of cell membrane repair machinery. Nat Cell Biol. 2009 Jan;11(1):56–64.

15. Li H, Duann P, Li Z, Zhou X, Ma J, Rovin BH, et al. The cell membrane repair protein MG53 modulates transcription factor NF-κB signaling to control kidney fibrosis. Kidney Int. 2022 Jan;101(1):119–30.

16. Eremina V, Jefferson JA, Kowalewska J, Hochster H, Haas M, Weisstuch J, et al. VEGF inhibition and renal thrombotic microangiopathy. N Engl J Med. 2008 Mar 13;358(11):1129–36.

17. Tsilosani A, Gao C, Zhang W. Aldosterone-Regulated Sodium Transport and Blood Pressure. Front Physiol. 2022;13:770375.

18. Johnston JG, Welch AK, Cain BD, Sayeski PP, Gumz ML, Wingo CS. Aldosterone: Renal Action and Physiological Effects. Compr Physiol. 2023 Mar 30;13(2):4409–91.

19. Wagner CA. Effect of mineralocorticoids on acid-base balance. Nephron Physiol. 2014;128(1–2):26–34.

20. Warren AM, Grossmann M, Christ-Crain M, Russell N. Syndrome of Inappropriate Antidiuresis: From Pathophysiology to Management. Endocr Rev. 2023 Sep 15;44(5):819–61.

21. Boone M, Deen PMT. Physiology and pathophysiology of the vasopressin-regulated renal water reabsorption. Pflugers Arch. 2008 Sep;456(6):1005–24.

22. Cuzzo B, Padala SA, Lappin SL. Physiology, Vasopressin. In: StatPearls [Internet]. Treasure Island (FL): StatPearls Publishing; 2025 [cited 2025 May 10]. Available from: http://www.ncbi.nlm.nih.gov/books/NBK526069/

23. Sankrityayan H, Rao PD, Shelke V, Kulkarni YA, Mulay SR, Gaikwad AB. Endoplasmic Reticulum Stress and Renin-Angiotensin System Crosstalk in Endothelial Dysfunction. Curr Mol Pharmacol. 2023;16(2):139–46.

24. Rodríguez-Soriano J. New insights into the pathogenesis of renal tubular acidosis--from functional to molecular studies. Pediatr Nephrol Berl Ger. 2000 Oct;14(12):1121–36.

25. Mustaqeem R, Arif A. Renal Tubular Acidosis. In: StatPearls [Internet]. Treasure Island (FL): StatPearls Publishing; 2025 [cited 2025 May 10]. Available from: http://www.ncbi.nlm.nih.gov/books/NBK519044/

26. Klein JD, Blount MA, Sands JM. Urea transport in the kidney. Compr Physiol. 2011 Apr;1(2):699–729.

27. Fenton RA, Yang B. Urea transporter knockout mice and their renal phenotypes. Subcell Biochem. 2014;73:137–52.

28. Bankir L, Yang B. New insights into urea and glucose handling by the kidney, and the urine concentrating mechanism. Kidney Int. 2012 Jun;81(12):1179–98.

29. Shayakul C, Clémençon B, Hediger MA. The urea transporter family (SLC14): physiological, pathological and structural aspects. Mol Aspects Med. 2013;34(2–3):313–22.

30. Zhou L, Meng Y, Lei T, Zhao D, Su J, Zhao X, et al. UT-B-deficient mice develop renal dysfunction and structural damage. BMC Nephrol. 2012 Jan 30;13:6.

31. Hebert SC, Mount DB, Gamba G. Molecular physiology of cation-coupled Cl- cotransport: the SLC12 family. Pflugers Arch. 2004 Feb;447(5):580–93.

32. Bazúa-Valenti S, Castañeda-Bueno M, Gamba G. Physiological role of SLC12 family members in the kidney. Am J Physiol Renal Physiol. 2016 Jul 1;311(1):F131-144.

33. Walker V. The Intricacies of Renal Phosphate Reabsorption-An Overview. Int J Mol Sci. 2024 Apr 25;25(9):4684.

34. Huo X, Liu K. Renal organic anion transporters in drug-drug interactions and diseases. Eur J Pharm Sci Off J Eur Fed Pharm Sci. 2018 Jan 15;112:8–19.

35. Saito H. Pathophysiological regulation of renal SLC22A organic ion transporters in acute kidney injury: pharmacological and toxicological implications. Pharmacol Ther. 2010 Jan;125(1):79–91.

36. Ailabouni A, Prasad B. Organic cation transporters 2: Structure, regulation, functions, and clinical implications. Drug Metab Dispos Biol Fate Chem. 2025 Mar;53(3):100044.

37. Lv J, Yu H, Du S, Xu P, Zhao Y, Qi W, et al. Targeting endoplasmic reticulum stress: an innovative therapeutic strategy for podocyte-related kidney diseases. J Transl Med. 2025 Jan 21;23(1):95.

38. He J, Yang B. Aquaporins in Renal Diseases. Int J Mol Sci. 2019 Jan 16;20(2):366.

39. Klaassen CD, Aleksunes LM. Xenobiotic, bile acid, and cholesterol transporters: function and regulation. Pharmacol Rev. 2010 Mar;62(1):1–96.
