# Supplementary material for: Renal adverse events in EGFR-TKI treatment: Comprehensive characterization of clinical patterns and molecular underpinnings
Source: Genes Dis. 2025 Nov 28;13(4):101953. doi: 10.1016/j.gendis.2025.101953 (PMC12993402; doi:10.1016/j.gendis.2025.101953)
Supplement: Table S1 — Clinical characteristic analysis of cancer patients with EGFR-TKIs as the primary suspected drug in the FAERS database (January 2013 to December 2023) [file mmc2.docx]

Supplementary Table 1: Clinical characteristics of tumor patients using EGFR-TKIs as included in the analysis sourced from the FAERS database.

|  |  |  |  |  |
| --- | --- | --- | --- | --- |
|  | **Characteristics** | **Count** | **Percent(%)** |  |
|  | **Age groups (years)** |  |  |  |
|  | ≥65 | 10649 | 34.4 |  |
|  | <65 | 6273 | 20.3 |  |
|  | Unknown or missing | 14001 | 45.3 |  |
|  | **Gender** |  |  |  |
|  | Female | 14991 | 48.5 |  |
|  | Male | 9139 | 29.5 |  |
|  | Unknown or missing | 6793 | 22.0 |  |
|  | **Suspected drugs** |  |  |  |
|  | Afatinib | 3737 | 12.1 |  |
|  | Dacomitinib | 177 | 0.6 |  |
|  | Erlotinib | 16735 | 54.1 |  |
|  | Gefitinib | 1877 | 6.1 |  |
|  | Osimertinib | 8397 | 27.1 |  |
|  | **Outcome** |  |  |  |
|  | DE | 9001 | 29.1 |  |
|  | DS | 220 | 0.7 |  |
|  | HO | 5295 | 17.1 |  |
|  | LT | 360 | 1.2 |  |
|  | OT | 9124 | 29.5 |  |
|  | RI | 8 | <0.1 |  |
|  | CA | 5 | <0.1 |  |
|  | Unknown or missing | 6910 | 22.4 |  |
|  | **Top 5 reporting countries** |  |  |  |
|  | US | 11432 | 37.0 |  |
|  | GB | 6974 | 22.6 |  |
|  | JP | 3144 | 10.2 |  |
|  | CN | 2022 | 6.5 |  |
|  | FR | 715 | 2.3 |  |
|  | **Total** | 30923 | 100.0 |  |
|  | Abbreviations:EGFR-TKIs,epidermal growth factor receptor tyrosine kinase inhibitors;FAERS,the FDA adverse event reporting system;DE,Death;DS,Disability;HO,Hospitalization - Initial or Prolonged;LT,Life-Threatening;OT,Other Serious (Important Medical Event);RI,Required Intervention to Prevent Permanent Impairment/Damage;CA,Congenital Anomaly;US,the United States;GB,Great Britain;JB,Japan;CN,China;FR,France. | | |  |
