# Supplementary material for: Renal adverse events in EGFR-TKI treatment: Comprehensive characterization of clinical patterns and molecular underpinnings
Source: Genes Dis. 2025 Nov 28;13(4):101953. doi: 10.1016/j.gendis.2025.101953 (PMC12993402; doi:10.1016/j.gendis.2025.101953)
Supplement: Table S2 — Clinical characteristic analysis of cancer patients with EGFR-TKIs as the primary suspected drug in the VigiBase database (through December 2023) [file mmc3.docx]

|  |  |  |  |  |
| --- | --- | --- | --- | --- |
|  | **Characteristics** | **Count** | **Percent(%)** |  |
|  | **Age groups (years)** |  |  |  |
|  | ≥65 | 18312 | 36.0 |  |
|  | <65 | 11137 | 21.9 |  |
|  | Unknown or missing | 21447 | 42.1 |  |
|  | **Gender** |  |  |  |
|  | Female | 28158 | 55.3 |  |
|  | Male | 20729 | 40.7 |  |
|  | Unknown or missing | 2009 | 4.0 |  |
|  | **Suspected drugs** |  |  |  |
|  | Afatinib | 5218 | 10.3 |  |
|  | Dacomitinib | 563 | 1.1 |  |
|  | Erlotinib | 31406 | 61.7 |  |
|  | Gefitinib | 4369 | 8.6 |  |
|  | Osimertinib | 9340 | 18.3 |  |
|  | **Outcome** |  |  |  |
|  | DE | 16859 | 33.1 |  |
|  | Other | 15615 | 30.7 |  |
|  | Unknown or missing | 18422 | 36.2 |  |
|  | **Seriousness** |  |  |  |
|  | Serious | 36691 | 72.1 |  |
|  | Not serious | 14129 | 27.8 |  |
|  | Unknown or missing | 76 | 0.1 |  |
|  | **Reporting region** |  |  |  |
|  | Americas | 32815 | 64.5 |  |
|  | European | 10903 | 21.4 |  |
|  | Western Pacific | 6124 | 12.0 |  |
|  | South-East Asia | 932 | 1.8 |  |
|  | Eastern Mediterranean | 106 | 0.2 |  |
|  | African | 16 | <0.1 |  |
|  | **Total** | 50896 | 100.0 |  |
|  | Abbreviations:EGFR-TKIs,epidermal growth factor receptor tyrosine kinase inhibitors;DE,Death. | | |  |

Supplementary Table 2: Clinical characteristics of tumor patients using EGFR-TKIs as included in the analysis sourced from the VigiBase database.
