# Supplementary material for: Renal adverse events in EGFR-TKI treatment: Comprehensive characterization of clinical patterns and molecular underpinnings
Source: Genes Dis. 2025 Nov 28;13(4):101953. doi: 10.1016/j.gendis.2025.101953 (PMC12993402; doi:10.1016/j.gendis.2025.101953)
Supplement: Table S3 — Clinical characteristics of patients experiencing EGFR-TKI-related renal adverse events in the FAERS database (January 2013 to December 2023). [file mmc4.docx]

|  | **Characteristics** | **Count** | **Percent(%)** |  |
| --- | --- | --- | --- | --- |
|  | **Age groups (years)** |  |  |  |
|  | ≥65 | 377 | 52.7 |  |
|  | <65 | 150 | 20.9 |  |
|  | Unknown or missing | 189 | 26.4 |  |
|  | **Gender** |  |  |  |
|  | Female | 317 | 44.3 |  |
|  | male | 238 | 33.2 |  |
|  | Unknown or missing | 161 | 22.5 |  |
|  | **Reporting year** |  |  |  |
|  | 2013 | 43 | 6.0 |  |
|  | 2014 | 60 | 8.4 |  |
|  | 2015 | 91 | 12.7 |  |
|  | 2016 | 88 | 12.3 |  |
|  | 2017 | 111 | 15.5 |  |
|  | 2018 | 89 | 12.4 |  |
|  | 2019 | 74 | 10.3 |  |
|  | 2020 | 49 | 6.8 |  |
|  | 2021 | 53 | 7.4 |  |
|  | 2022 | 19 | 2.7 |  |
|  | 2023 | 39 | 5.4 |  |
|  | **Suspected drugs** |  |  |  |
|  | Afatinib | 183 | 25.6 |  |
|  | Dacomitinib | 8 | 1.1 |  |
|  | Erlotinib | 321 | 44.8 |  |
|  | Gefitinib | 43 | 6.0 |  |
|  | Osimertinib | 161 | 22.5 |  |
|  | **Outcome** |  |  |  |
|  | DE | 91 | 12.7 |  |
|  | DS | 7 | 1.0 |  |
|  | HO | 260 | 36.3 |  |
|  | LT | 20 | 2.8 |  |
|  | OT | 304 | 42.5 |  |
|  | Unknown or missing | 34 | 4.7 |  |
|  | **Top 5 reporting countries** |  |  |  |
|  | US | 175 | 24.4 |  |
|  | GB | 146 | 20.4 |  |
|  | JP | 144 | 20.1 |  |
|  | CN | 42 | 5.9 |  |
|  | FR | 29 | 4.1 |  |
|  | **Total** | 716 | 100.0 |  |
|  | Abbreviations : EGFR-TKI,epidermal growth factor receptor tyrosine kinase inhibitors;FAERS,the FDA adverse event reporting system;DE,Death;DS,Disability;HO,Hospitalization - Initial or Prolonged;LT,Life-Threatening;OT,Other Serious (Important Medical Event);RI,Required Intervention to Prevent Permanent Impairment/Damage;CA,Congenital Anomaly;US,the United States;GB,Great Britain;JB,Japan;CN,China;FR,France. | | |  |

Supplementary Table 3: Clinical characteristics of patients with EGFR-TKIs associated renal adverse effects sourced from the FAERS database.
