# Supplementary material for: Renal adverse events in EGFR-TKI treatment: Comprehensive characterization of clinical patterns and molecular underpinnings
Source: Genes Dis. 2025 Nov 28;13(4):101953. doi: 10.1016/j.gendis.2025.101953 (PMC12993402; doi:10.1016/j.gendis.2025.101953)
Supplement: Table S4 — Clinical characteristics of patients experiencing EGFR-TKI-related renal adverse events in the VigiBase database (through December 2023). [file mmc5.docx]

Supplementary Table 4: Clinical characteristics of patients with EGFR-TKIs associated renal adverse effects sourced from the VigiBase database.

|  |  |  |  |  |
| --- | --- | --- | --- | --- |
|  | **Characteristics** | **Count** | **Percent(%)** |  |
|  | **Age groups (years)** |  |  |  |
|  | ≥65 | 526 | 52.3 |  |
|  | <65 | 244 | 24.2 |  |
|  | Unknown or missing | 236 | 23.5 |  |
|  | **Gender** |  |  |  |
|  | Female | 527 | 52.4 |  |
|  | male | 451 | 44.8 |  |
|  | Unknown or missing | 28 | 2.8 |  |
|  | **Reporting year** |  |  |  |
|  | <2013 | 275 | 27.3 |  |
|  | 2013 | 72 | 7.2 |  |
|  | 2014 | 77 | 7.7 |  |
|  | 2015 | 131 | 13.0 |  |
|  | 2016 | 89 | 8.8 |  |
|  | 2017 | 100 | 9.9 |  |
|  | 2018 | 93 | 9.2 |  |
|  | 2019 | 51 | 5.1 |  |
|  | 2020 | 30 | 3.0 |  |
|  | 2021 | 31 | 3.1 |  |
|  | 2022 | 30 | 3.0 |  |
|  | 2023 | 27 | 2.7 |  |
|  | **Suspected drugs** |  |  |  |
|  | Afatinib | 217 | 21.6 |  |
|  | Dacomitinib | 3 | 0.3 |  |
|  | Erlotinib | 562 | 55.9 |  |
|  | Gefitinib | 116 | 11.5 |  |
|  | Osimertinib | 108 | 10.7 |  |
|  | **Outcome** |  |  |  |
|  | DE | 224 | 22.3 |  |
|  | Other | 586 | 58.2 |  |
|  | Unknown or missing | 196 | 19.5 |  |
|  | **Seriousness** |  |  |  |
|  | Serious | 928 | 92.2 |  |
|  | Not serious | 75 | 7.5 |  |
|  | Unknown or missing | 3 | 0.3 |  |
|  | **Reporting region** |  |  |  |
|  | Americas | 578 | 57.5 |  |
|  | European | 284 | 28.2 |  |
|  | Western Pacific | 139 | 13.8 |  |
|  | South-East Asia | 4 | 0.4 |  |
|  | Eastern Mediterranean | 1 | 0.1 |  |
|  | **Total** | 1006 | 100.0 |  |
|  | Abbreviations : EGFR-TKIs,epidermal growth factor receptor tyrosine kinase inhibitors;DE,Death. | | |  |
