# Supplementary material for: Renal adverse events in EGFR-TKI treatment: Comprehensive characterization of clinical patterns and molecular underpinnings
Source: Genes Dis. 2025 Nov 28;13(4):101953. doi: 10.1016/j.gendis.2025.101953 (PMC12993402; doi:10.1016/j.gendis.2025.101953)
Supplement: Table S5 — Statistical analysis results of renal adverse events and concurrent adverse events associated with EGFR-TKIs. [file mmc6.docx]

**Supplementary Table 5. Statistical analysis results of renal adverse events and concurrent adverse events associated with EGFR-TKIs.**

| **FAERS database** | | | **VigiBase database** | | |
| --- | --- | --- | --- | --- | --- |
| PT | Number | p | PT | Number | p |
| Diarrhoea | 263 | <2e-16 | Diarrhoea | 415 | <2e-16 |
| Rash | 116 | 0.467 | Dehydration | 186 | <2e-16 |
| Dehydration | 105 | <2e-16 | Rash | 148 | 0.236 |
| Nausea | 97 | <2e-16 | Vomiting | 141 | <2e-16 |
| Vomiting | 91 | <2e-16 | Nausea | 139 | <2e-16 |
| Decreased appetite | 85 | <2e-16 | Fatigue | 98 | 1.08e-12 |
| Malignant neoplasm progression | 66 | 0.0178 | Decreased appetite | 92 | 2.48e-15 |
| Fatigue | 64 | 1.47e-05 | Asthenia | 72 | 2.42e-15 |
| Anaemia | 59 | <2e-16 | Dyspnoea | 70 | 9.52e-11 |
| Asthenia | 50 | 6.62e-10 | Death | 65 | <2e-16 |
| Dyspnoea | 46 | 4.73e-06 | Anaemia | 64 | <2e-16 |
| Death | 45 | <2e-16 | Malignant neoplasm progression | 63 | 0.549 |
| Hypertension | 39 | <2e-16 | Weight decreased | 55 | 1.54e-09 |
| Blood creatinine increased | 36 | <2e-16 | Hypotension | 54 | <2e-16 |
| Off label use | 36 | 0.0925 | Pneumonia | 51 | 2.16e-07 |
| Weight decreased | 35 | 0.000156 | Blood creatinine increased | 45 | <2e-16 |
| Constipation | 33 | 2.37e-11 | Pyrexia | 44 | 2.41e-11 |
| Pneumonia | 33 | 5.61e-05 | Off label use | 42 | 0.0245 |
| Stomatitis | 30 | 0.0228 | Urinary tract infection | 42 | <2e-16 |
| Abdominal pain | 28 | 4.68e-12 | Cough | 41 | 1.01e-06 |
| Thrombocytopenia | 28 | <2e-16 | Abdominal pain | 38 | 9.65e-14 |
| Alopecia | 27 | 0.0109 | Alopecia | 35 | 0.00115 |
| Dry skin | 27 | 0.807 | Dry skin | 34 | 0.721 |
| Hypotension | 26 | 5.26e-15 | Dizziness | 33 | 2.98e-10 |
| Pyrexia | 26 | 0.000821 | Stomatitis | 32 | 0.0125 |
| Pruritus | 25 | 0.594 | Hypertension | 31 | <2e-16 |
| Urinary tract infection | 24 | <2e-16 | Pain | 31 | 0.000871 |
| Oedema peripheral | 24 | 2.08e-12 | Pleural effusion | 30 | 7.87e-07 |
| Dermatitis acneiform | 23 | 0.000234 | Thrombocytopenia | 30 | 2.83e-14 |
| Neutropenia | 23 | 1.74e-10 | Pruritus | 29 | 0.973 |
| Pleural effusion | 23 | 0.00551 | Respiratory failure | 29 | 5.19e-16 |
| Paronychia | 22 | 0.00154 | Oedema peripheral | 29 | <2e-16 |
| Hypokalaemia | 22 | 3.75e-13 | Constipation | 29 | 4.35e-06 |
| Epistaxis | 21 | 1.9e-06 | Hyponatraemia | 29 | 2.49e-16 |
| Hyponatraemia | 21 | 6.88e-12 | Sepsis | 28 | 3.35e-14 |
| Interstitial lung disease | 21 | 0.0738 | Hypokalaemia | 28 | 6.43e-16 |
| Cough | 20 | 0.238 | Disease progression | 27 | 0.982 |
| Pain | 20 | 0.0937 | Fall | 25 | 9.29e-09 |
| Malaise | 20 | 0.0922 | Hypophagia | 24 | 1.06e-12 |
| Platelet count decreased | 19 | 3.22e-07 | Epistaxis | 24 | 5.38e-06 |
| Dizziness | 19 | 0.0058 | Hyperkalaemia | 23 | <2e-16 |
| Haemorrhage | 19 | 1.74e-09 | General physical health deterioration | 23 | 3.9e-11 |
| Hepatic function abnormal | 18 | 1.2e-07 | Back pain | 22 | 3.56e-07 |
| Sepsis | 18 | 5.55e-08 | Mental status changes | 22 | 7.6e-15 |
| Mucosal inflammation | 17 | 2.48e-07 | Muscle spasms | 21 | 7.52e-06 |
| Acne | 17 | 0.841 | Tachycardia | 19 | 4.48e-13 |
| Aspartate aminotransferase increased | 15 | 2.44e-07 | Headache | 19 | 0.00854 |
| Infection | 15 | 6.41e-05 | Malaise | 19 | 1 |
| Alanine aminotransferase increased | 15 | 9.61e-07 | Abdominal pain upper | 19 | 0.000523 |
| Neuropathy peripheral | 14 | 1.89e-06 | Pulmonary embolism | 18 | 0.00807 |
| Respiratory failure | 14 | 0.0176 | Atrial fibrillation | 18 | 1.7e-07 |
| Cardiac failure | 14 | 0.000144 | Acne | 18 | 0.872 |
| Fall | 14 | 0.176 | Metastases to liver | 18 | 2.57e-06 |
| Muscle spasms | 14 | 0.000843 | Neutropenia | 17 | 7.07e-06 |
| Pulmonary embolism | 14 | 0.0133 | Dermatitis acneiform | 17 | 1 |
| Insomnia | 14 | 0.000417 | Chest pain | 17 | 7.98e-05 |
| Atrial fibrillation | 13 | 2.22e-05 | Mucosal inflammation | 16 | 0.0198 |
| Abdominal pain upper | 13 | 0.0182 | Arthralgia | 16 | 0.00157 |
| Arthralgia | 13 | 0.00189 | Abdominal distension | 15 | 2.77e-06 |
| Abdominal distension | 13 | 1.67e-06 | Blood bilirubin increased | 15 | 1.02e-07 |
| Leukopenia | 13 | 4.36e-07 | Peripheral swelling | 15 | 2.5e-05 |
| Hypophagia | 12 | 1.66e-05 | Confusional state | 15 | 0.00162 |
| Deep vein thrombosis | 12 | 0.000148 | Cardiac failure | 15 | 3.19e-05 |
| Oedema | 12 | 4.37e-06 | Platelet count decreased | 15 | 3.12e-05 |
| Back pain | 12 | 0.0329 | Cellulitis | 14 | 3.03e-07 |
| Headache | 12 | 0.459 | Hypovolaemia | 14 | 2.47e-16 |
| General physical health deterioration | 11 | 0.053 | Muscular weakness | 14 | 1.78e-05 |
| Septic shock | 11 | 2.99e-08 | Depression | 14 | 0.000341 |
| Skin exfoliation | 11 | 0.418 | Infection | 14 | 0.00244 |
| Peripheral swelling | 11 | 0.021 | Insomnia | 14 | 0.000388 |
| Liver disorder | 10 | 0.00243 | Paronychia | 14 | 0.808 |
| Hepatic failure | 10 | 1.51e-07 | Hepatic failure | 13 | 2.03e-11 |
| Hyperkalaemia | 10 | 6.39e-08 | Staphylococcal infection | 13 | 3.01e-08 |
| Depression | 10 | 0.00255 | Deep vein thrombosis | 13 | 0.000796 |
| Skin disorder | 9 | 0.192 | Toxicity to various agents | 13 | 8.54e-06 |
| Dysuria | 9 | 7.03e-06 | Metastases to central nervous system | 13 | 0.49 |
| Nail disorder | 9 | 0.0496 | Haemorrhage | 13 | 0.000224 |
| Pain in extremity | 9 | 0.503 | Haemoglobin decreased | 13 | 0.000767 |
| Muscular weakness | 9 | 0.00257 | International normalised ratio increased | 12 | 4.05e-07 |
| Eating disorder | 9 | 6.5e-05 | Febrile neutropenia | 12 | 0.000176 |
| Dry mouth | 9 | 0.132 | Chills | 12 | 0.000689 |
| Erythema | 9 | 0.656 | Multiple organ dysfunction syndrome | 12 | 6.7e-10 |
| Dry eye | 9 | 0.223 | Drug ineffective | 12 | 0.00273 |
| Hypoalbuminaemia | 8 | 1.69e-06 | Hypoxia | 12 | 6.54e-05 |
| Haemoglobin decreased | 8 | 0.00347 | Gastrointestinal haemorrhage | 12 | 0.00119 |
| Drug ineffective | 8 | 0.000259 | Myocardial infarction | 12 | 0.00196 |
| Cellulitis | 8 | 0.000284 | Aspartate aminotransferase increased | 12 | 0.000218 |
| Anxiety | 8 | 0.02 | Dysuria | 12 | 1.33e-07 |
| Confusional state | 8 | 0.0735 | Lethargy | 12 | 2.19e-06 |
| Dysphagia | 8 | 0.526 | Neuropathy peripheral | 12 | 0.000282 |
| Myocardial infarction | 8 | 0.0192 | Metabolic acidosis | 12 | 2.5e-14 |
| Metastases to central nervous system | 8 | 0.317 | Hypercalcaemia | 11 | 4.61e-11 |
| Skin ulcer | 8 | 0.095 | Septic shock | 11 | 1.48e-07 |
| Chest pain | 8 | 0.154 | Vision blurred | 11 | 0.0213 |
| Skin discolouration | 8 | 0.00116 | Transaminases increased | 11 | 3.79e-05 |
| Drug resistance | 8 | 0.00165 | Skin exfoliation | 11 | 0.919 |
| Blood glucose increased | 7 | 1.08e-05 | Dyspepsia | 11 | 0.183 |
| Vision blurred | 7 | 0.204 | Anxiety | 11 | 0.00244 |
| Blood pressure decreased | 7 | 0.000166 | Pain in extremity | 11 | 0.425 |
| Tachycardia | 7 | 0.000268 | Blood sodium decreased | 10 | 5.15e-06 |
| Metabolic acidosis | 7 | 1.06e-09 | Liver disorder | 10 | 0.00445 |
| Cerebrovascular accident | 7 | 0.211 | Myalgia | 10 | 0.00236 |
| Abdominal discomfort | 7 | 0.669 | Seizure | 10 | 0.0269 |
| Gastrooesophageal reflux disease | 7 | 0.103 | Liver function test abnormal | 10 | 7.14e-06 |
| Flatulence | 7 | 0.00409 | Pericardial effusion | 10 | 0.000227 |
| Syncope | 7 | 0.00464 | Visual impairment | 10 | 0.00913 |
| Dysgeusia | 7 | 0.732 | Oedema | 10 | 0.000652 |
| Dyspepsia | 7 | 0.716 | Thrombotic microangiopathy | 10 | 1.49e-13 |
| Pneumonitis | 7 | 0.708 | Gastrooesophageal reflux disease | 10 | 0.0593 |
| Cardiac failure congestive | 7 | 0.00699 | Dry mouth | 10 | 0.152 |
| Myalgia | 7 | 0.0256 | Weight increased | 9 | 0.000283 |
| Onychoclasis | 7 | 0.348 | Blood pressure increased | 9 | 0.000246 |
| Incorrect dose administered | 6 | 0.947 | Hepatic function abnormal | 9 | 0.00808 |
| Visual impairment | 6 | 0.294 | Syncope | 9 | 0.00152 |
| Gait disturbance | 6 | 0.642 | Alanine aminotransferase increased | 9 | 0.0171 |
| Metastases to bone | 6 | 0.931 | Nail disorder | 9 | 0.28 |
| Febrile neutropenia | 6 | 0.0289 | Dry eye | 9 | 0.371 |
| Thrombosis | 6 | 0.471 | Abdominal discomfort | 9 | 0.317 |
| Palpitations | 6 | 0.00167 | Dysphagia | 9 | 0.72 |
| Neoplasm progression | 6 | 0.31 | Dysgeusia | 9 | 0.443 |
| Blood alkaline phosphatase increased | 6 | 0.00081 | Incorrect dose administered | 9 | 0.697 |
| Lymphadenopathy | 6 | 0.00526 | Contusion | 9 | 9.7e-05 |
| Contusion | 6 | 0.00738 | Cerebrovascular accident | 8 | 0.526 |
| Blood magnesium decreased | 6 | 4.55e-05 | Gait disturbance | 8 | 0.0678 |
| Blood potassium decreased | 6 | 0.0049 | Interstitial lung disease | 8 | 1.32e-05 |
| Seizure | 6 | 0.0626 | Thrombosis | 8 | 0.317 |
| Nasopharyngitis | 6 | 0.459 | Hyperhidrosis | 8 | 0.000187 |
| Delirium | 5 | 0.0012 | Skin discolouration | 8 | 0.0011 |
| Electrolyte imbalance | 5 | 0.00197 | Drug intolerance | 8 | 0.0612 |
| Hyperglycaemia | 5 | 0.00197 | Gastrointestinal disorder | 8 | 0.00877 |
| White blood cell count decreased | 5 | 0.411 | Cardiac arrest | 8 | 0.00167 |
| Burning sensation | 5 | 0.0286 | Nephrolithiasis | 8 | 1.05e-05 |
| Hypoxia | 5 | 0.0332 | Eating disorder | 8 | 0.000222 |
| Hypovolaemia | 5 | 7.24e-06 | Dyspnoea exertional | 8 | 0.00444 |
| Blood bilirubin increased | 5 | 0.0207 | Onychoclasis | 8 | 0.544 |
| Blood urine present | 5 | 0.00338 | Pneumonia aspiration | 8 | 0.000157 |
| Gastritis | 5 | 0.00494 | Skin lesion | 8 | 0.00198 |
| Pneumonia aspiration | 5 | 0.0117 | Cardiac failure congestive | 8 | 0.00259 |
| Skin lesion | 5 | 0.0134 | Haematochezia | 8 | 0.000488 |
| Gastric ulcer | 5 | 0.0064 | Urine output decreased | 8 | 3.61e-08 |
| Thrombotic microangiopathy | 5 | 3.86e-08 | Hypomagnesaemia | 8 | 5.87e-05 |
| Gastrointestinal disorder | 5 | 0.109 | Lung neoplasm malignant | 8 | 1 |
| Haemoptysis | 5 | 0.613 | Skin toxicity | 8 | 1 |
| Mouth ulceration | 5 | 0.193 | Bacterial infection | 8 | 5.26e-07 |
| Pericardial effusion | 5 | 0.103 | Somnolence | 8 | 0.00948 |
| Metastases to liver | 5 | 0.616 | Gastritis | 8 | 8.87e-05 |
| Rash pruritic | 5 | 0.603 | Disseminated intravascular coagulation | 8 | 1.21e-06 |
| Blood creatine phosphokinase increased | 5 | 0.011 | Oropharyngeal pain | 8 | 0.0173 |
| Chills | 5 | 0.0603 | Flatulence | 8 | 0.00532 |
| Ascites | 5 | 0.0184 | Arthritis | 7 | 0.000244 |
| Embolism | 5 | 0.0134 | Hyperbilirubinaemia | 7 | 0.000144 |
| Hair texture abnormal | 5 | 0.234 | Nervous system disorder | 7 | 4.81e-05 |
| Skin fissures | 5 | 1 | Leukocytosis | 7 | 1.22e-05 |
| Faeces discoloured | 5 | 0.00137 | Blood glucose increased | 7 | 0.000903 |
| Eye discharge | 5 | 0.0126 | Pancytopenia | 7 | 0.00208 |
| Lacrimation increased | 5 | 0.0537 | Ascites | 7 | 0.00171 |
| Eye pain | 5 | 0.0674 | Blood potassium increased | 7 | 7.26e-07 |
| Multiple organ dysfunction syndrome | 5 | 0.000194 | Pneumonitis | 7 | 0.382 |
| Disseminated intravascular coagulation | 5 | 0.0012 | Rash pruritic | 7 | 0.244 |
| Memory impairment | 5 | 0.202 | Haemoptysis | 7 | 0.637 |
| Cardiac disorder | 5 | 0.0775 | Blood urea increased | 7 | 0.000103 |
| Cystitis | 4 | 0.0639 | Leukopenia | 7 | 0.0209 |
| Overdose | 4 | 0.0216 | Faeces discoloured | 7 | 0.000199 |
| Hepatitis | 4 | 0.0103 | Flank pain | 7 | 2.01e-06 |
| Hyperbilirubinaemia | 4 | 0.000533 | Paraesthesia | 7 | 0.031 |
| Haematochezia | 4 | 0.0729 | Blood urine present | 7 | 0.000129 |
| Atelectasis | 4 | 0.00546 | White blood cell count decreased | 7 | 0.0902 |
| Pancytopenia | 4 | 0.0698 | Rhabdomyolysis | 7 | 2.52e-06 |
| Diabetes mellitus | 4 | 0.00245 | Dysphonia | 7 | 0.00918 |
| Lung disorder | 4 | 0.433 | Blood albumin decreased | 7 | 7.08e-06 |
| Transaminases increased | 4 | 0.0342 | Hyperglycaemia | 7 | 0.000161 |
| Arthritis | 4 | 0.0159 | Haematocrit decreased | 7 | 0.000606 |
| C-reactive protein increased | 4 | 0.00178 | Lung disorder | 7 | 1 |
| Chronic obstructive pulmonary disease | 4 | 0.0406 | Loss of consciousness | 7 | 0.0105 |
| Drug intolerance | 4 | 0.554 | Skin disorder | 7 | 0.792 |
| Pneumothorax | 4 | 0.547 | Memory impairment | 7 | 0.0301 |
| Disease progression | 4 | 0.0256 | Tremor | 7 | 0.0169 |
| Urticaria | 4 | 0.586 | Mass | 6 | 0.00946 |
| Hepatic enzyme increased | 4 | 0.0729 | Blood magnesium decreased | 6 | 0.000316 |
| International normalised ratio increased | 4 | 0.00612 | Candida infection | 6 | 0.00119 |
| Nasal congestion | 4 | 0.00484 | Feeling abnormal | 6 | 0.154 |
| Blood pressure increased | 4 | 0.131 | Vertigo | 6 | 0.00543 |
| Mental status changes | 4 | 0.00762 | Chronic obstructive pulmonary disease | 6 | 0.0422 |
| Arrhythmia | 4 | 0.00327 | Bacteraemia | 6 | 3.34e-06 |
| Blister | 4 | 1 | Mouth ulceration | 6 | 0.0553 |
| Flank pain | 4 | 0.000673 | Skin fissures | 6 | 0.86 |
| Metastases to lung | 4 | 0.366 | Wheezing | 6 | 0.00303 |
| Somnolence | 4 | 0.324 | Jaundice | 6 | 0.00166 |
| Palmar-plantar erythrodysaesthesia syndrome | 4 | 0.119 | Red blood cell count decreased | 6 | 0.000398 |
| Tumour lysis syndrome | 4 | 8.26e-05 | Osteopenia | 6 | 2.52e-07 |
| Paraesthesia | 4 | 0.298 | Skin ulcer | 6 | 0.485 |
| Feeling abnormal | 4 | 0.311 | Acute respiratory failure | 6 | 0.00946 |
| Furuncle | 4 | 0.00427 | Bone pain | 6 | 0.0383 |
| Hypocalcaemia | 4 | 0.00427 | Melaena | 6 | 0.000904 |
| Productive cough | 4 | 0.156 | Embolism | 6 | 0.00245 |
| Cataract | 4 | 0.0528 | Ill-defined disorder | 6 | 0.0121 |
| Influenza | 4 | 0.0892 | Erythema | 6 | 0.0873 |
| Enterocolitis | 4 | 0.00125 | Malignant pleural effusion | 6 | 0.00166 |
| Urine output decreased | 4 | 0.000837 | Electrolyte imbalance | 6 | 0.00028 |
| Glomerular filtration rate decreased | 4 | 0.000173 | Ulcer | 6 | 0.00576 |
| Amylase increased | 4 | 0.0147 | Non-small cell lung cancer | 6 | 0.303 |
| Pollakiuria | 4 | 0.0201 | Cystitis | 6 | 0.011 |
| Trichiasis | 4 | 0.000673 | Gastric ulcer | 6 | 0.00141 |
| Hair growth abnormal | 4 | 0.374 | Lymphadenopathy | 5 | 0.0346 |
| Altered state of consciousness | 4 | 0.00612 | Condition aggravated | 5 | 0.111 |
| Alanine aminotransferase abnormal | 4 | 1.85e-05 | Blood alkaline phosphatase increased | 5 | 0.0332 |
| Electrocardiogram QT prolonged | 4 | 0.303 | Feeling cold | 5 | 0.00929 |
| Aphasia | 4 | 0.0322 | Frequent bowel movements | 5 | 0.000677 |
| Lactic acidosis | 3 | 4.86e-05 | Skin infection | 5 | 0.0126 |
| Leukocytosis | 3 | 0.00908 | Overdose | 5 | 0.00277 |
| Metastases to peritoneum | 3 | 0.0195 | Neoplasm progression | 5 | 0.988 |
| Cardiac arrest | 3 | 0.191 | Pulmonary oedema | 5 | 0.429 |
| Orthostatic hypotension | 3 | 0.00457 | Influenza | 5 | 0.0375 |
| Oral candidiasis | 3 | 0.0174 | Hair texture abnormal | 5 | 0.252 |
| Hypophosphataemia | 3 | 0.00297 | Burning sensation | 5 | 0.0784 |
| Small intestinal haemorrhage | 3 | 0.000404 | Tumour lysis syndrome | 5 | 6.07e-08 |
| Ejection fraction decreased | 3 | 0.0949 | Hepatic cirrhosis | 5 | 5.16e-06 |
| Urinary retention | 3 | 0.0216 | Hepatic enzyme increased | 5 | 0.263 |
| Malignant pleural effusion | 3 | 0.109 | Acute hepatic failure | 5 | 6.93e-07 |
| Tinnitus | 3 | 0.0403 | Delirium | 5 | 0.000114 |
| Drug interaction | 3 | 0.258 | Lung infiltration | 5 | 0.036 |
| Skin infection | 3 | 0.0949 | Urinary retention | 5 | 0.000168 |
| Performance status decreased | 3 | 0.073 | Malnutrition | 5 | 0.00077 |
| Bronchitis | 3 | 0.179 | Recurrent cancer | 5 | 0.00171 |
| Liver function test abnormal | 3 | 0.065 | Hair growth abnormal | 5 | 0.41 |
| Osteopenia | 3 | 0.00131 | Skin reaction | 5 | 0.41 |
| Jaundice | 3 | 0.0573 | Metastases to bone | 5 | 1 |
| Dyspnoea exertional | 3 | 0.162 | Fungal infection | 5 | 0.0245 |
| Skin toxicity | 3 | 0.486 | Hepatitis | 5 | 0.00253 |
| Swelling | 3 | 0.258 | Hypocalcaemia | 5 | 0.00459 |
| Hip fracture | 3 | 0.114 | Hip fracture | 5 | 0.0119 |
| Hepatic cirrhosis | 3 | 0.00178 | Hypoalbuminaemia | 5 | 0.000448 |
| Loss of consciousness | 3 | 0.433 | Pneumothorax | 5 | 0.234 |
| Rash erythematous | 3 | 0.749 | Hepatic encephalopathy | 5 | 2.07e-05 |
| Coma | 3 | 0.135 | Coma | 5 | 0.00424 |
| Visual acuity reduced | 3 | 0.0815 | Colitis | 5 | 0.0105 |
| Oral pain | 3 | 1 | Organ failure | 5 | 3.52e-07 |
| Adverse drug reaction | 3 | 0.0573 | Pollakiuria | 5 | 0.00535 |
| Clostridium difficile colitis | 3 | 0.00372 | Aphasia | 5 | 0.0213 |
| Condition aggravated | 3 | 0.493 | Productive cough | 5 | 0.0853 |
| Staphylococcal infection | 3 | 0.0689 | Hypoglycaemia | 5 | 0.00154 |
| Blood sodium decreased | 3 | 0.0403 | Adverse drug reaction | 5 | 0.00929 |
| Metastasis | 3 | 0.517 | Neoplasm malignant | 4 | 1 |
| Disorientation | 3 | 0.0344 | Dialysis | 4 | 7.47e-07 |
| Cerebral infarction | 3 | 0.74 | Pancreatitis | 4 | 0.106 |
| Growth of eyelashes | 3 | 0.245 | Hypervolaemia | 4 | 0.00013 |
| Hypothyroidism | 3 | 0.0155 | Blood creatine phosphokinase increased | 4 | 0.0207 |
| Laboratory test abnormal | 3 | 0.00457 | Acidosis | 4 | 5.06e-06 |
| Oropharyngeal pain | 3 | 0.767 | Failure to thrive | 4 | 0.0182 |
| Ageusia | 3 | 0.482 | Metastatic neoplasm | 4 | 0.0458 |
| Amnesia | 3 | 0.436 | Arteriosclerosis | 4 | 9.41e-05 |
| Neoplasm malignant | 3 | 0.433 | Acute respiratory distress syndrome | 4 | 0.0326 |
| Candida infection | 3 | 0.0468 | Influenza like illness | 4 | 0.011 |
| Dysphonia | 3 | 0.264 | Body temperature increased | 4 | 0.0182 |
| Frequent bowel movements | 3 | 0.0155 | Bradycardia | 4 | 0.000835 |
| Pancreatitis | 3 | 0.124 | Renal cyst | 4 | 9.41e-05 |
| Skin abrasion | 3 | 0.000635 | Glomerular filtration rate decreased | 4 | 4.48e-05 |
| Discomfort | 3 | 0.239 | Neurological symptom | 4 | 0.00118 |
| Joint swelling | 3 | 0.209 | Shock | 4 | 9.41e-05 |
| Nephrolithiasis | 3 | 0.0611 | Fluid retention | 4 | 0.00721 |
| Bone pain | 3 | 0.509 | Eye pain | 4 | 0.312 |
| Fungal infection | 3 | 0.151 | Bronchitis | 4 | 0.106 |
| Shock | 3 | 0.00457 | Oral pain | 4 | 1 |
| Rash generalised | 3 | 1 | Metastases to lung | 4 | 0.374 |
| Lymphopenia | 3 | 0.073 | Tachypnoea | 4 | 0.000571 |
| Eye pruritus | 3 | 0.751 | Sleep apnoea syndrome | 4 | 9.97e-06 |
| Ulcer | 3 | 0.209 | Hypothyroidism | 4 | 0.00541 |
| Glossitis | 3 | 0.0216 | Oral candidiasis | 4 | 0.00936 |
| Cardiac tamponade | 3 | 0.00778 | Hepatorenal syndrome | 4 | 9.97e-06 |
| Speech disorder | 3 | 0.114 | Joint swelling | 4 | 0.14 |
| Clostridium difficile infection | 3 | 0.0502 | Arrhythmia | 4 | 0.00213 |
| Bone marrow failure | 3 | 0.203 | Herpes zoster | 4 | 0.147 |
| Hair disorder | 3 | 0.0689 | Palpitations | 4 | 0.0458 |
| Metastases to meninges | 3 | 1 | Amnesia | 4 | 0.178 |
| Viral infection | 3 | 0.0105 | Disorientation | 4 | 0.0293 |
| Acute respiratory failure | 3 | 0.129 | Blood creatine increased | 4 | 6.62e-05 |
| Pulmonary alveolar haemorrhage | 3 | 0.0174 | Atelectasis | 4 | 0.0262 |
| Blood potassium increased | 3 | 0.00131 | Cardiomegaly | 4 | 0.000294 |
| Mass | 3 | 0.203 | Musculoskeletal discomfort | 4 | 0.000835 |
| Hepatotoxicity | 3 | 0.065 | Hypoaesthesia | 4 | 1 |
| Weight increased | 3 | 0.439 | Blood glucose decreased | 4 | 0.00394 |
| Eye irritation | 3 | 1 | Photophobia | 4 | 0.00488 |
| Photophobia | 3 | 0.0195 | Cardiac disorder | 4 | 0.151 |
| Lung infection | 3 | 0.759 | Nasal congestion | 4 | 0.0148 |
| Pneumonia bacterial | 3 | 0.0537 | Nasopharyngitis | 4 | 0.578 |
| Feeding disorder | 3 | 0.748 | Thermal burn | 4 | 0.000571 |
| Nail discolouration | 3 | 0.0996 | Haemorrhoids | 4 | 0.0086 |
| Gastroenteritis | 3 | 0.0373 | Rheumatoid nodule | 4 | 1.52e-07 |
| Retroperitoneal fibrosis | 3 | 4.86e-05 | Blood pressure decreased | 4 | 0.0567 |
| Aspartate aminotransferase abnormal | 3 | 0.00131 | Lacrimation increased | 4 | 0.364 |
| Rash maculo-papular | 3 | 0.124 | Swelling | 4 | 0.352 |
| Hypoaesthesia | 3 | 1 | Product dose omission issue | 4 | 1 |
| Urinary incontinence | 3 | 0.0435 | Cholelithiasis | 4 | 0.00936 |
| Ingrowing nail | 3 | 0.129 | Gait inability | 4 | 0.0417 |
| COVID-19 | 3 | 0.168 | Eye discharge | 4 | 0.0522 |
| Hepatitis acute | 2 | 0.00311 | Hepatomegaly | 4 | 0.000694 |
| Tachypnoea | 2 | 0.0773 | Drug interaction | 4 | 0.151 |
| Chest discomfort | 2 | 1 | Aortic stenosis | 3 | 4e-04 |
| Toothache | 2 | 0.0256 | Lung cancer metastatic | 3 | 0.455 |
| Uterine leiomyoma | 2 | 0.00755 | Diabetes mellitus | 3 | 0.0143 |
| Mouth haemorrhage | 2 | 0.0706 | Cerebral infarction | 3 | 0.435 |
| Wrong technique in drug usage process | 2 | 0.00311 | Neutrophil count decreased | 3 | 0.467 |
| Aortic stenosis | 2 | 0.0137 | Blepharitis | 3 | 0.0991 |
| Hypoglycaemia | 2 | 0.0912 | Cardio-respiratory arrest | 3 | 0.138 |
| Type 2 diabetes mellitus | 2 | 0.0104 | Micturition disorder | 3 | 4e-04 |
| Multi-organ failure | 2 | 0.0303 | Blood lactate dehydrogenase increased | 3 | 0.00503 |
| Device related infection | 2 | 0.0579 | Generalised oedema | 3 | 0.00356 |
| Angina pectoris | 2 | 0.0405 | Haemolytic uraemic syndrome | 3 | 0.000147 |
| Convulsion | 2 | 0.145 | Aspiration | 3 | 0.0175 |
| Renal function test abnormal | 2 | 0.00158 | Neck pain | 3 | 0.166 |
| Herpes zoster | 2 | 1 | Growth of eyelashes | 3 | 0.447 |
| Duodenal ulcer | 2 | 0.129 | Diverticulum | 3 | 0.00356 |
| Cardiomyopathy | 2 | 0.28 | Polyuria | 3 | 0.000833 |
| Coagulopathy | 2 | 0.0256 | Dementia | 3 | 0.0471 |
| Ecchymosis | 2 | 0.0173 | Rales | 3 | 4e-04 |
| Haemorrhage intracranial | 2 | 0.0405 | Erythema multiforme | 3 | 0.0873 |
| Arteriosclerosis | 2 | 0.0104 | Eye swelling | 3 | 0.166 |
| Neurological symptom | 2 | 0.0353 | Diverticulitis | 3 | 0.0873 |
| Osteoarthritis | 2 | 0.0912 | Blood culture positive | 3 | 0.0102 |
| Umbilical hernia | 2 | 0.00311 | Colonic fistula | 3 | 3.03e-05 |
| Urine flow decreased | 2 | 0.00511 | Blood potassium decreased | 3 | 0.44 |
| Vasculitis | 2 | 0.0706 | Pseudomonas infection | 3 | 0.00293 |
| Fluid retention | 2 | 0.137 | Dysarthria | 3 | 0.0592 |
| Cholecystitis acute | 2 | 0.0256 | Blister | 3 | 0.636 |
| Asthma | 2 | 0.245 | Cataract | 3 | 0.147 |
| Hypercalcaemia | 2 | 0.0641 | Wrong technique in product usage process | 3 | 0.025 |
| Influenza like illness | 2 | 0.113 | C-reactive protein increased | 3 | 0.00683 |
| Joint injury | 2 | 0.0256 | Feeding disorder | 3 | 0.458 |
| Neck pain | 2 | 0.365 | Cystitis haemorrhagic | 3 | 0.00426 |
| Rhinitis | 2 | 0.0579 | Pancreatic carcinoma | 3 | 0.151 |
| Vertigo | 2 | 0.186 | Blood lactic acid increased | 3 | 7.47e-05 |
| Body temperature increased | 2 | 0.113 | Haematemesis | 3 | 0.252 |
| White blood cell count increased | 2 | 0.121 | Osteoporosis | 3 | 0.00896 |
| Metastatic neoplasm | 2 | 0.263 | Pleuritic pain | 3 | 0.0158 |
| Nerve compression | 2 | 0.0461 | Respiratory disorder | 3 | 0.0592 |
| Spinal column stenosis | 2 | 0.0213 | Laboratory test abnormal | 3 | 0.0192 |
| Sudden death | 2 | 0.0773 | Palmar-plantar erythrodysaesthesia syndrome | 3 | 0.761 |
| Hyperhidrosis | 2 | 0.314 | Orthostatic hypotension | 3 | 0.0192 |
| Folliculitis | 2 | 0.145 | Inflammation | 3 | 0.442 |
| Fungal skin infection | 2 | 0.0461 | Rhinorrhoea | 3 | 1 |
| Henoch-Schonlein purpura | 2 | 0.0256 | Cerebral haemorrhage | 3 | 0.0727 |
| Hypoacusis | 2 | 0.28 | White blood cell count increased | 3 | 0.133 |
| Mental impairment | 2 | 0.0706 | Osteoarthritis | 3 | 0.0192 |
| Retching | 2 | 0.129 | Ileus | 3 | 0.133 |
| Glossodynia | 2 | 0.398 | Pain of skin | 3 | 0.452 |
| Nasal dryness | 2 | 0.656 | Sinusitis | 3 | 0.185 |
| Swollen tongue | 2 | 0.288 | Pyelonephritis | 3 | 0.000833 |
| Ocular icterus | 2 | 0.00755 | Treatment noncompliance | 3 | 0.021 |
| Throat irritation | 2 | 0.169 | Drug eruption | 3 | 0.111 |
| Nail infection | 2 | 0.39 | Adenocarcinoma | 3 | 0.0389 |
| Acne pustular | 2 | 0.0773 | Haemodialysis | 3 | 3.03e-05 |
| Musculoskeletal chest pain | 2 | 0.237 | Heart rate increased | 3 | 0.482 |
| Bacterial infection | 2 | 0.0773 | Eye irritation | 3 | 0.637 |
| Hypersensitivity | 2 | 1 | Hepatic mass | 3 | 0.00148 |
| Intestinal dilatation | 2 | 0.00158 | Peripheral coldness | 3 | 0.0114 |
| Swelling face | 2 | 0.696 | Rhinitis | 3 | 0.0143 |
| Hypomagnesaemia | 2 | 0.161 | Asthma | 3 | 0.0192 |
| Pruritus generalised | 2 | 0.169 | Joint injury | 3 | 0.00238 |
| Restlessness | 2 | 0.0579 | Mouth haemorrhage | 3 | 0.0143 |
| Haemorrhoids | 2 | 0.113 | Acne pustular | 3 | 0.0229 |
| Oral herpes | 2 | 0.145 | Laboratory test interference | 3 | 7.47e-05 |
| Peripheral coldness | 2 | 0.0579 | Lung consolidation | 3 | 0.00293 |
| Proctalgia | 2 | 0.0303 | Fungal skin infection | 3 | 0.00896 |
| Eczema | 2 | 0.373 | Skin abrasion | 3 | 0.00896 |
| Papule | 2 | 0.0256 | Bone disorder | 3 | 0.0128 |
| Blood iron decreased | 2 | 0.0256 | Visual acuity reduced | 3 | 0.12 |
| Lip swelling | 2 | 0.653 | Onychomadesis | 3 | 0.19 |
| Micturition disorder | 2 | 0.00511 | Performance status decreased | 3 | 0.129 |
| Skin haemorrhage | 2 | 0.186 | Glossodynia | 3 | 0.211 |
| Blood glucose decreased | 2 | 0.0706 | Metastases to adrenals | 3 | 0.0271 |
| Rash macular | 2 | 0.656 | Anal incontinence | 3 | 0.00785 |
| Temperature intolerance | 2 | 0.0256 | Biliary obstruction | 3 | 0.0128 |
| Acidosis | 2 | 0.000535 | Anaemia of chronic disease | 3 | 3.03e-05 |
| Blood lactate dehydrogenase increased | 2 | 0.0173 | Hypophosphataemia | 3 | 0.00683 |
| Intertrigo | 2 | 0.00311 | Folliculitis | 3 | 0.638 |
| Loose tooth | 2 | 0.00311 | Metabolic encephalopathy | 3 | 0.000592 |
| Colonic fistula | 2 | 0.00158 | Slow response to stimuli | 3 | 3.03e-05 |
| Brain oedema | 2 | 0.331 | Eye pruritus | 3 | 1 |
| Pelvic pain | 2 | 0.0256 | Renal function test abnormal | 3 | 0.000147 |
| Tenderness | 2 | 0.0461 | Disturbance in attention | 3 | 0.0128 |
| Pain of skin | 2 | 0.653 | Splenomegaly | 3 | 0.000147 |
| Ventricular hypokinesia | 2 | 0.0104 | Brain oedema | 3 | 0.0798 |
| Cancer pain | 2 | 0.113 | Uterine leiomyoma | 3 | 4e-04 |
| Inflammation | 2 | 0.661 | Obstruction | 3 | 0.0019 |
| Hiccups | 2 | 0.0461 | Ureteric obstruction | 3 | 7.47e-05 |
| Blood lactic acid increased | 2 | 0.000535 | Urinary tract obstruction | 3 | 0.000147 |
| Generalised oedema | 2 | 0.0405 | Enterocolitis | 3 | 0.025 |
| Hypercoagulation | 2 | 0.00158 | Enterococcal infection | 3 | 7.47e-05 |
| Pancreatitis acute | 2 | 0.0579 | Clostridium difficile infection | 3 | 0.0592 |
| Eyelid ptosis | 2 | 0.0303 | Hair disorder | 3 | 0.103 |
| Blepharitis | 2 | 0.0706 | Pancreatitis acute | 3 | 0.0835 |
| Cholangitis | 2 | 0.186 | Hiccups | 3 | 0.00896 |
| Ill-defined disorder | 2 | 0.684 | Coagulopathy | 3 | 0.0114 |
| Sinus disorder | 2 | 0.0461 | Retching | 3 | 0.0658 |
| Lymphocyte count decreased | 2 | 0.153 | Musculoskeletal chest pain | 3 | 0.0912 |
| Musculoskeletal pain | 2 | 1 | Rash erythematous | 3 | 1 |
| Small intestinal obstruction | 2 | 0.113 | Depressed level of consciousness | 3 | 0.0592 |
| Oesophageal haemorrhage | 2 | 0.00158 | Lung neoplasm | 3 | 0.0692 |
| Oesophagitis | 2 | 0.203 | Small intestinal obstruction | 3 | 0.142 |
| Blood creatine increased | 2 | 0.00311 | Hypercoagulation | 3 | 0.000833 |
| Blood creatinine abnormal | 2 | 0.00311 | Oesophageal haemorrhage | 3 | 0.000147 |
| Intestinal obstruction | 2 | 0.323 | Hepatic necrosis | 3 | 7.47e-05 |
| Peritonitis | 2 | 0.121 | Dermatitis | 3 | 0.635 |
| Lymphangiosis carcinomatosa | 2 | 0.398 | Gallbladder disorder | 3 | 0.00503 |
| Cardiac valve disease | 2 | 0.00158 | Metastasis | 3 | 1 |
| Hemiparesis | 2 | 0.153 | Tinnitus | 3 | 0.0592 |
| Ocular toxicity | 2 | 0.00755 | Urine flow decreased | 3 | 0.000254 |
| Acute hepatic failure | 2 | 0.0137 | Product administration error | 2 | 0.0226 |
| Klebsiella sepsis | 2 | 0.00311 | Metastases to peritoneum | 2 | 0.131 |
| Encephalopathy | 2 | 0.0641 | Hepatotoxicity | 2 | 0.678 |
| Hypovolaemic shock | 2 | 0.0173 | Localised oedema | 2 | 0.0191 |
| Acute myocardial infarction | 2 | 0.169 | Hemiplegia | 2 | 0.0191 |
| Pulmonary oedema | 2 | 0.591 | Refusal of treatment by relative | 2 | 0.00375 |
| Renal cyst | 2 | 0.0353 | Respiratory rate increased | 2 | 0.0535 |
| Hyperuricaemia | 2 | 0.00511 | Panic attack | 2 | 0.0128 |
| Eye swelling | 2 | 0.314 | Magnesium metabolism disorder | 2 | 0.00039 |
| Madarosis | 2 | 0.177 | Lower gastrointestinal haemorrhage | 2 | 0.0695 |
| Pulmonary toxicity | 2 | 0.186 | Exercise tolerance decreased | 2 | 0.00767 |
| Toxicity to various agents | 2 | 1 | Herpes simplex | 2 | 0.0128 |
| Dermatitis | 2 | 1 | Activated partial thromboplastin time prolonged | 2 | 0.0586 |
| Blood pressure abnormal | 2 | 0.0173 | Rash papular | 2 | 1 |
| Eye disorder | 2 | 1 | Abnormal faeces | 2 | 0.0346 |
| Ocular hyperaemia | 2 | 1 | Iron deficiency anaemia | 2 | 0.0303 |
| Onychomadesis | 2 | 0.382 | Encephalitis | 2 | 0.00767 |
| Onychomycosis | 2 | 0.161 | Blood calcium decreased | 2 | 0.0991 |
| Wheezing | 2 | 0.254 | Prothrombin time prolonged | 2 | 0.18 |
| Platelet disorder | 2 | 0.00311 | Urethral obstruction | 2 | 0.00039 |
| Drug eruption | 2 | 0.297 | Incontinence | 2 | 0.039 |
| Drug-induced liver injury | 2 | 0.675 | Ear neoplasm | 2 | 0.00116 |
| Recurrent cancer | 2 | 0.137 | Sensory disturbance | 2 | 0.0264 |
| Subdural haematoma | 2 | 0.0641 | Glycosuria | 2 | 0.00039 |
| Liver injury | 2 | 0.314 | Intertrigo | 2 | 0.0226 |
| Sleep disorder | 2 | 0.137 | Haemorrhage urinary tract | 2 | 0.00116 |
| Toxic skin eruption | 2 | 0.0641 | Blood calcium increased | 2 | 0.0191 |
| Metabolic disorder | 2 | 0.0104 | Campylobacter infection | 2 | 0.00039 |
| Metastases to adrenals | 2 | 0.106 | Rectal haemorrhage | 2 | 0.655 |
| Pericarditis | 2 | 0.0353 | Aortic arteriosclerosis | 2 | 0.0101 |
| Brain neoplasm | 2 | 0.365 | Device related infection | 2 | 0.118 |
| Presyncope | 2 | 0.0256 | Malignant neoplasm of spinal cord | 2 | 0.00116 |
| Disturbance in attention | 2 | 0.0461 | Lymphopenia | 2 | 0.369 |
| Blood bilirubin abnormal | 2 | 0.00311 | Skin haemorrhage | 2 | 0.238 |
| Salivary duct inflammation | 2 | 0.000535 | Duodenal ulcer | 2 | 0.201 |
| Localised oedema | 2 | 0.00755 | Rash macular | 2 | 0.715 |
| Cardiac failure acute | 2 | 0.0842 | Cachexia | 2 | 0.125 |
| Gliosis | 2 | 0.00158 | Madarosis | 2 | 0.187 |
| Nervous system disorder | 2 | 0.129 | Stevens-Johnson syndrome | 2 | 0.376 |
| Colitis | 2 | 0.28 | Spinal osteoarthritis | 2 | 0.0436 |
| Thirst | 2 | 0.0461 | Therapy non-responder | 2 | 0.664 |
| Melaena | 2 | 0.137 | Gastric haemorrhage | 2 | 0.0929 |
| Cachexia | 2 | 0.113 | Spinal stenosis | 2 | 0.0191 |
| Hemiplegia | 2 | 0.0173 | Proctalgia | 2 | 0.0264 |
| Macular oedema | 2 | 0.00311 | Pneumonia bacterial | 2 | 0.131 |
| Ulcerative keratitis | 2 | 0.145 | Cardiac failure acute | 2 | 0.0264 |
| Venous thrombosis | 2 | 0.0518 | Agitation | 2 | 0.145 |
| Metastases to pleura | 2 | 0.129 | Intestinal ischaemia | 2 | 0.0436 |
| Blood urea increased | 2 | 0.0842 | Diplopia | 2 | 0.187 |
| Metabolic encephalopathy | 2 | 0.00511 | Nail growth abnormal | 2 | 0.152 |
| Conjunctivitis | 2 | 1 | Bone marrow failure | 2 | 0.0809 |
| Rhabdomyolysis | 2 | 0.129 | Basal cell carcinoma | 2 | 0.0436 |
| Bone disorder | 2 | 0.0461 | Blood creatinine abnormal | 2 | 0.0101 |
| Upper gastrointestinal haemorrhage | 2 | 0.0579 | Megacolon | 2 | 0.00555 |
| Carcinoembryonic antigen increased | 2 | 0.709 | Renal tubular injury | 2 | 0.00039 |
| Gingival bleeding | 2 | 0.145 | Large intestine perforation | 2 | 0.138 |
| Pulmonary thrombosis | 2 | 0.271 | Inappropriate schedule of product administration | 2 | 0.18 |
| Pallor | 1 | 0.131 | Discomfort | 2 | 0.311 |
| Neutrophil count decreased | 1 | 0.731 | Therapeutic product ineffective | 2 | 0.152 |
| Campylobacter infection | 1 | 0.0232 | Urticaria | 2 | 0.78 |
| Ear discomfort | 1 | 0.227 | Urinary tract infection pseudomonal | 2 | 0.00039 |
| Migraine | 1 | 0.389 | Impaired healing | 2 | 0.304 |
| Hydroureter | 1 | 0.0232 | Skin laceration | 2 | 0.355 |
| Non-small cell lung cancer | 1 | 0.389 | Type 2 diabetes mellitus | 2 | 0.0128 |
| Renal failure chronic | 1 | 0.0232 | Panophthalmitis | 2 | 0.00116 |
| Upper limb fracture | 1 | 0.456 | Migraine | 2 | 0.125 |
| Arthritis bacterial | 1 | 0.0679 | Chest discomfort | 2 | 0.694 |
| Bursitis infective | 1 | 0.0232 | Clostridium difficile colitis | 2 | 0.166 |
| Diabetic nephropathy | 1 | 0.0232 | Radiation pneumonitis | 2 | 0.0929 |
| Pyuria | 1 | 0.0232 | Conjunctivitis | 2 | 0.214 |
| Adenocarcinoma pancreas | 1 | 0.0458 | Eye disorder | 2 | 1 |
| Bacterial sepsis | 1 | 0.0679 | Dysstasia | 2 | 0.0929 |
| Intra-abdominal haematoma | 1 | 0.0232 | Acute interstitial pneumonitis | 2 | 0.0264 |
| Immune thrombocytopenic purpura | 1 | 0.131 | Gastrointestinal infection | 2 | 0.0128 |
| Cerebellar haemorrhage | 1 | 0.0458 | Brain neoplasm malignant | 2 | 0.112 |
| International normalised ratio abnormal | 1 | 0.0895 | Urine leukocyte esterase positive | 2 | 0.00116 |
| Gastroenteritis clostridial | 1 | 0.0458 | Post-traumatic neck syndrome | 2 | 0.00116 |
| Erosive oesophagitis | 1 | 0.111 | Abdominal pain lower | 2 | 0.0484 |
| Stenotrophomonas infection | 1 | 0.0232 | Venous thrombosis | 2 | 0.0226 |
| Suicidal ideation | 1 | 0.171 | Intentional product use issue | 2 | 1 |
| Abdominal pain lower | 1 | 0.171 | Hypertensive emergency | 2 | 0.00039 |
| Cytomegalovirus test | 1 | 0.0232 | Neoplasm | 2 | 1 |
| Pneumonia pseudomonal | 1 | 0.0458 | Blood iron decreased | 2 | 0.039 |
| Increased upper airway secretion | 1 | 0.171 | Lip swelling | 2 | 0.653 |
| Paraplegia | 1 | 0.0895 | Nephropathy toxic | 2 | 0.0191 |
| Anaemia macrocytic | 1 | 0.0895 | Pyelocaliectasis | 2 | 0.00039 |
| Hyperlipidaemia | 1 | 0.0458 | Temperature intolerance | 2 | 0.0303 |
| Sleep apnoea syndrome | 1 | 0.0895 | Radiation oesophagitis | 2 | 0.00767 |
| Gastric perforation | 1 | 0.171 | Sinus tachycardia | 2 | 0.131 |
| Glomerulonephropathy | 1 | 0.0232 | Urosepsis | 2 | 0.0226 |
| Intussusception | 1 | 0.0895 | Abdominal sepsis | 2 | 0.00039 |
| Suture related complication | 1 | 0.0232 | Nasal dryness | 2 | 0.694 |
| Drug administration error | 1 | 0.131 | Loose tooth | 2 | 0.00375 |
| Cardiac death | 1 | 0.0679 | Toothache | 2 | 0.0346 |
| Blood pressure fluctuation | 1 | 0.28 | Furuncle | 2 | 0.231 |
| Local swelling | 1 | 0.528 | Swelling face | 2 | 1 |
| Cervical vertebral fracture | 1 | 0.131 | Swollen tongue | 2 | 0.369 |
| Ovarian cancer recurrent | 1 | 0.0232 | Full blood count abnormal | 2 | 0.138 |
| Adverse event | 1 | 0.296 | Nerve compression | 2 | 0.0128 |
| Biliary tract disorder | 1 | 0.0895 | Lymphoedema | 2 | 0.0346 |
| Cholelithiasis | 1 | 0.599 | Hepatic lesion | 2 | 0.0436 |
| Basal cell carcinoma | 1 | 0.263 | Exostosis | 2 | 0.00228 |
| Brain neoplasm malignant | 1 | 0.43 | Oral herpes | 2 | 0.152 |
| Iron deficiency anaemia | 1 | 0.245 | Unresponsive to stimuli | 2 | 0.105 |
| Magnesium metabolism disorder | 1 | 0.0232 | Weight fluctuation | 2 | 0.0484 |
| Malignant neoplasm of spinal cord | 1 | 0.0458 | Oesophagobronchial fistula | 2 | 0.00116 |
| Osteoporosis | 1 | 0.344 | Functional gastrointestinal disorder | 2 | 0.00767 |
| Pleuritic pain | 1 | 0.329 | Nail discolouration | 2 | 0.383 |
| Post-traumatic neck syndrome | 1 | 0.0458 | Prostatic disorder | 2 | 0.00767 |
| Urine leukocyte esterase positive | 1 | 0.0458 | Trichiasis | 2 | 0.064 |
| Alkalosis hypokalaemic | 1 | 0.0232 | Blood uric acid increased | 2 | 0.0101 |
| Blood pressure | 1 | 0.0232 | Psychotic disorder | 2 | 0.0264 |
| Miliaria | 1 | 0.0679 | Thrombotic thrombocytopenic purpura | 2 | 0.0303 |
| Lung neoplasm malignant | 1 | 1 | Mobility decreased | 2 | 0.105 |
| Heart rate increased | 1 | 1 | Tenderness | 2 | 0.105 |
| Panic attack | 1 | 0.0895 | Pelvic pain | 2 | 0.0264 |
| Phobia | 1 | 0.0232 | Viral infection | 2 | 0.187 |
| Stent placement | 1 | 0.0679 | Adrenal disorder | 2 | 0.00228 |
| Urinary tract obstruction | 1 | 0.111 | Hypogammaglobulinaemia | 2 | 0.00116 |
| Vomiting projectile | 1 | 0.0458 | Streptococcal urinary tract infection | 2 | 0.00039 |
| Lung adenocarcinoma metastatic | 1 | 0.0895 | Exposure to radiation | 2 | 0.00039 |
| Dysstasia | 1 | 0.344 | Ventricular tachycardia | 2 | 0.0809 |
| Peripheral embolism | 1 | 0.111 | Onychomycosis | 2 | 0.166 |
| Inappropriate schedule of drug administration | 1 | 0.443 | Ingrowing nail | 2 | 0.666 |
| Lung consolidation | 1 | 0.131 | Urinary incontinence | 2 | 0.112 |
| Catheter site pain | 1 | 0.0458 | Blood pressure abnormal | 2 | 0.0535 |
| Urethral obstruction | 1 | 0.0232 | Gastrointestinal toxicity | 2 | 0.376 |
| Hip arthroplasty | 1 | 0.171 | Ventricular extrasystoles | 2 | 0.0346 |
| Polyp | 1 | 0.0458 | Ejection fraction decreased | 2 | 0.668 |
| Computerised tomogram abnormal | 1 | 0.151 | Respiratory distress | 2 | 0.673 |
| Oesophageal obstruction | 1 | 0.0895 | Platelet count | 2 | 0.00228 |
| Tongue discolouration | 1 | 0.28 | White blood cell count | 2 | 0.0158 |
| Hepatomegaly | 1 | 0.296 | Hypovolaemic shock | 2 | 0.0264 |
| Penile swelling | 1 | 0.0232 | Culture urine positive | 2 | 0.0101 |
| Petechiae | 1 | 0.209 | Sinus disorder | 2 | 0.0436 |
| Rheumatoid nodule | 1 | 0.0232 | Throat irritation | 2 | 0.223 |
| Skin reaction | 1 | 1 | Ocular hyperaemia | 2 | 1 |
| Vascular calcification | 1 | 0.0679 | Speech disorder | 2 | 0.699 |
| Hyperproteinaemia | 1 | 0.0232 | Mental impairment | 2 | 0.0436 |
| Hyperventilation | 1 | 0.0679 | Sudden death | 2 | 0.253 |
| Limb discomfort | 1 | 0.313 | Henoch-Schonlein purpura | 2 | 0.039 |
| Night sweats | 1 | 0.43 | Bedridden | 2 | 0.064 |
| Sputum discoloured | 1 | 0.245 | Gingival bleeding | 2 | 0.0991 |
| Upper extremity mass | 1 | 0.0232 | Movement disorder | 2 | 0.138 |
| Nosocomial infection | 1 | 0.0458 | Magnetic resonance imaging head abnormal | 2 | 0.0191 |
| Neutropenic sepsis | 1 | 0.111 | Lipase increased | 2 | 0.152 |
| Tumour haemorrhage | 1 | 0.227 | Amylase increased | 2 | 0.159 |
| Blood sodium increased | 1 | 0.0458 | Lung adenocarcinoma | 2 | 0.245 |
| Gastrointestinal motility disorder | 1 | 0.245 | Hiatus hernia | 2 | 0.0695 |
| Genital herpes | 1 | 0.0679 | Lymphoma | 2 | 0.0191 |
| Proctitis | 1 | 0.151 | Xeroderma | 2 | 0.0695 |
| Protein urine | 1 | 0.0458 | Oesophageal ulcer | 2 | 0.0809 |
| Purulent discharge | 1 | 0.171 | Duodenal stenosis | 2 | 0.00767 |
| Rectal abscess | 1 | 0.111 | Oesophageal stenosis | 2 | 0.0484 |
| Malignant ascites | 1 | 0.151 | Acute myocardial infarction | 2 | 0.245 |
| Painful respiration | 1 | 0.171 | Kidney infection | 2 | 0.0586 |
| Mycobacterium chelonae infection | 1 | 0.0232 | Seborrhoeic dermatitis | 2 | 0.0303 |
| Heart rate irregular | 1 | 0.296 | Large intestinal ulcer | 2 | 0.00228 |
| Neoplasm | 1 | 0.732 | Rash pustular | 2 | 0.168 |
| Rhinalgia | 1 | 0.151 | Hypoacusis | 2 | 0.659 |
| Diverticulum intestinal | 1 | 0.0679 | Ocular icterus | 2 | 0.0158 |
| Large intestine perforation | 1 | 0.296 | Rectal ulcer | 2 | 0.0128 |
| Oral mucosal eruption | 1 | 0.296 | Cardiac tamponade | 2 | 0.064 |
| Glycosuria | 1 | 0.0232 | Immune thrombocytopenia | 2 | 0.0226 |
| Rash papular | 1 | 0.58 | Computerised tomogram abnormal | 2 | 0.0868 |
| Haemorrhage urinary tract | 1 | 0.0458 | Petechiae | 2 | 0.0868 |
| Hernia | 1 | 0.263 | Vascular calcification | 2 | 0.00555 |
| Hydrocele | 1 | 0.0458 | Encephalopathy | 2 | 0.105 |
| Hyperplasia | 1 | 0.0679 | Motor dysfunction | 2 | 0.0346 |
| Ileus | 1 | 0.58 | Escherichia urinary tract infection | 2 | 0.00555 |
| Joint effusion | 1 | 0.0679 | Nail infection | 2 | 0.696 |
| Scrotal swelling | 1 | 0.0232 | Heart rate decreased | 2 | 0.0484 |
| Spinal osteoarthritis | 1 | 0.209 | Cognitive disorder | 2 | 0.223 |
| Urinary bladder haemorrhage | 1 | 0.19 | Alcohol use | 2 | 0.00039 |
| Ear neoplasm | 1 | 0.0232 | Autoimmune disorder | 2 | 0.00116 |
| Excoriation | 1 | 0.0895 | Hyperproteinaemia | 2 | 0.00039 |
| Musculoskeletal discomfort | 1 | 0.296 | Limb discomfort | 2 | 0.0484 |
| Sensory disturbance | 1 | 0.19 | Night sweats | 2 | 0.187 |
| Lymphoedema | 1 | 0.28 | Limb mass | 2 | 0.00767 |
| Non-cardiac chest pain | 1 | 0.171 | Sputum discoloured | 2 | 0.0586 |
| Adenocarcinoma | 1 | 0.209 | Hyperventilation | 2 | 0.00375 |
| Bile duct obstruction | 1 | 0.209 | Anaemia macrocytic | 2 | 0.00228 |
| Emotional distress | 1 | 0.19 | Hyperlipidaemia | 2 | 0.00555 |
| Facial pain | 1 | 0.389 | Umbilical hernia | 2 | 0.00555 |
| Foot deformity | 1 | 0.0895 | Klebsiella sepsis | 2 | 0.00555 |
| Lasegue's test negative | 1 | 0.0232 | Oesophageal varices haemorrhage | 2 | 0.0226 |
| Renal cancer | 1 | 0.0895 | Toxic skin eruption | 2 | 0.216 |
| Respiratory tract congestion | 1 | 0.151 | Gastroenteritis | 2 | 0.0809 |
| Rhinitis allergic | 1 | 0.131 | Congenital absence of bile ducts | 1 | 0.0198 |
| Sinus operation | 1 | 0.151 | Spinal disorder | 1 | 0.13 |
| Spinal pain | 1 | 0.344 | Granulocytopenia | 1 | 0.095 |
| Splenomegaly | 1 | 0.0458 | Prothrombin level increased | 1 | 0.0391 |
| Nasal discomfort | 1 | 0.626 | Bursitis infective | 1 | 0.0581 |
| Procedural complication | 1 | 0.0679 | Renal arteriosclerosis | 1 | 0.0198 |
| Oesophageal spasm | 1 | 0.0232 | Occult blood | 1 | 0.0581 |
| Osteosclerosis | 1 | 0.111 | Vascular occlusion | 1 | 0.13 |
| Rales | 1 | 0.0679 | Metabolic disorder | 1 | 0.164 |
| Sinusitis | 1 | 1 | Cholangitis infective | 1 | 0.0768 |
| Weight fluctuation | 1 | 0.296 | Rhinalgia | 1 | 0.181 |
| Product use issue | 1 | 1 | Blood disorder | 1 | 0.259 |
| Fluid intake reduced | 1 | 0.245 | Fluid imbalance | 1 | 0.0198 |
| Intestinal ischaemia | 1 | 0.19 | Neutropenic colitis | 1 | 0.0391 |
| Peritoneal adhesions | 1 | 0.0232 | Arterial haemorrhage | 1 | 0.0768 |
| Skin irritation | 1 | 1 | Gastrointestinal motility disorder | 1 | 0.273 |
| Appendicitis perforated | 1 | 0.0232 | Urine ketone body present | 1 | 0.13 |
| Intentional product misuse | 1 | 0.57 | Retroperitoneal fibrosis | 1 | 0.0391 |
| Blood creatinine decreased | 1 | 0.0895 | Renal tubular atrophy | 1 | 0.0198 |
| Oral dysaesthesia | 1 | 0.0458 | Carnitine | 1 | 0.0198 |
| Hunger | 1 | 0.209 | Lymphangiosis carcinomatosa | 1 | 0.483 |
| Oral mucosal blistering | 1 | 0.59 | Alkalosis hypokalaemic | 1 | 0.0198 |
| Seborrhoeic dermatitis | 1 | 0.296 | Drug resistance | 1 | 0.0114 |
| Dementia | 1 | 0.516 | Gliosis | 1 | 0.0391 |
| Slow response to stimuli | 1 | 0.0458 | Pulmonary mass | 1 | 1 |
| Gout | 1 | 0.245 | Joint stiffness | 1 | 0.273 |
| Shock symptom | 1 | 0.0232 | Pseudomembranous colitis | 1 | 0.164 |
| Angioplasty | 1 | 0.0232 | Device dislocation | 1 | 0.0198 |
| Blood calcium increased | 1 | 0.131 | Normochromic normocytic anaemia | 1 | 0.0581 |
| Faeces hard | 1 | 0.0895 | Nervousness | 1 | 0.381 |
| Bacterial translocation | 1 | 0.0232 | Illness | 1 | 0.513 |
| Affect lability | 1 | 0.0458 | Oesophageal spasm | 1 | 0.0198 |
| Dermatitis diaper | 1 | 0.111 | Hepatic cytolysis | 1 | 0.568 |
| Ocular discomfort | 1 | 0.263 | Emotional distress | 1 | 0.229 |
| Xerosis | 1 | 0.245 | Blood creatinine decreased | 1 | 0.095 |
| Sinus tachycardia | 1 | 0.227 | Diabetes insipidus | 1 | 0.0581 |
| Occult blood | 1 | 0.0679 | Muscle haemorrhage | 1 | 0.095 |
| Food aversion | 1 | 0.0458 | Vitamin B12 deficiency | 1 | 0.0581 |
| Nail growth abnormal | 1 | 0.456 | Metastatic squamous cell carcinoma | 1 | 0.0768 |
| Ingrown hair | 1 | 0.0232 | Trismus | 1 | 0.095 |
| Bradycardia | 1 | 0.171 | Localised infection | 1 | 1 |
| Troponin increased | 1 | 0.151 | Hydrocele | 1 | 0.0581 |
| Abdominal infection | 1 | 0.0679 | Malignant ascites | 1 | 0.113 |
| Gastrointestinal pain | 1 | 0.329 | Purulent discharge | 1 | 0.229 |
| Breath sounds abnormal | 1 | 0.263 | Hallucination | 1 | 1 |
| Creatinine renal clearance decreased | 1 | 0.131 | Transfusion reaction | 1 | 0.0391 |
| Decubitus ulcer | 1 | 0.389 | Appetite disorder | 1 | 0.368 |
| Anal fungal infection | 1 | 0.0232 | Pulmonary hypertension | 1 | 0.316 |
| Fear | 1 | 0.0679 | Red blood cell elliptocytes present | 1 | 0.0198 |
| Generalised tonic-clonic seizure | 1 | 0.209 | Red blood cell morphology abnormal | 1 | 0.0198 |
| Oral fungal infection | 1 | 0.171 | Thinking abnormal | 1 | 0.113 |
| Gastrointestinal haemorrhage | 1 | 0.729 | Heart rate irregular | 1 | 0.368 |
| Photosensitivity reaction | 1 | 0.456 | Metastases to pleura | 1 | 0.503 |
| Glucose tolerance impaired | 1 | 0.0458 | Bicytopenia | 1 | 0.0391 |
| Left ventricular dysfunction | 1 | 0.359 | Ageusia | 1 | 0.525 |
| Appendicitis | 1 | 0.151 | Spinal cord oedema | 1 | 0.0198 |
| Sensory level abnormal | 1 | 0.0232 | Radiation injury | 1 | 0.181 |
| Pancreatic mass | 1 | 0.0458 | Subcutaneous emphysema | 1 | 0.0581 |
| Duodenal ulcer haemorrhage | 1 | 0.227 | Procedural complication | 1 | 0.113 |
| Pneumonia cryptococcal | 1 | 0.0458 | Faeces hard | 1 | 0.0768 |
| Sluggishness | 1 | 0.111 | Altered state of consciousness | 1 | 0.343 |
| Cardiac failure chronic | 1 | 0.263 | Breast disorder | 1 | 0.0391 |
| Facial paralysis | 1 | 0.374 | Staphylococcal sepsis | 1 | 0.13 |
| Pulmonary congestion | 1 | 0.417 | Affect lability | 1 | 0.0391 |
| Aphthous ulcer | 1 | 0.539 | Blepharospasm | 1 | 0.0581 |
| Diabetic retinopathy | 1 | 0.0458 | Ear discomfort | 1 | 0.229 |
| Blood alkaline phosphatase abnormal | 1 | 0.0679 | Duodenal ulcer haemorrhage | 1 | 0.259 |
| Prothrombin time abnormal | 1 | 0.0232 | Bacterial translocation | 1 | 0.0198 |
| Acute respiratory distress syndrome | 1 | 0.58 | Hepatic pain | 1 | 0.164 |
| Food poisoning | 1 | 0.131 | Tardive dyskinesia | 1 | 0.0768 |
| Gangrene | 1 | 0.131 | Benign prostatic hyperplasia | 1 | 0.0391 |
| Body temperature decreased | 1 | 0.171 | Enteritis infectious | 1 | 0.0581 |
| Renal hypertrophy | 1 | 0.0232 | Arthropathy | 1 | 0.244 |
| Vulvovaginal pain | 1 | 0.111 | Intestinal dilatation | 1 | 0.0581 |
| Hepatic infarction | 1 | 0.0232 | Muscle disorder | 1 | 0.095 |
| Hepatic necrosis | 1 | 0.0895 | Ovarian mass | 1 | 0.0391 |
| Hepatic vein thrombosis | 1 | 0.0232 | Gastroenteritis clostridial | 1 | 0.0391 |
| Megacolon | 1 | 0.0679 | Glomerulosclerosis | 1 | 0.0391 |
| Pleural disorder | 1 | 0.0895 | Blood urea nitrogen/creatinine ratio increased | 1 | 0.0391 |
| Metastatic squamous cell carcinoma | 1 | 0.0679 | Helicobacter gastritis | 1 | 0.0198 |
| Hair colour changes | 1 | 0.417 | Food intolerance | 1 | 0.213 |
| Radiation injury | 1 | 0.111 | Poor peripheral circulation | 1 | 0.164 |
| Trismus | 1 | 0.0458 | Appendicitis perforated | 1 | 0.0581 |
| Cell death | 1 | 0.0458 | Upper gastrointestinal haemorrhage | 1 | 0.428 |
| Ototoxicity | 1 | 0.0458 | Nasal disorder | 1 | 0.181 |
| Ventricular dysfunction | 1 | 0.131 | Haematoma | 1 | 0.417 |
| Ureteric obstruction | 1 | 0.0232 | Eyelid ptosis | 1 | 0.213 |
| Arthropod bite | 1 | 0.0679 | Blood chloride decreased | 1 | 0.288 |
| Heat illness | 1 | 0.0895 | Metastases to heart | 1 | 0.181 |
| Motion sickness | 1 | 0.209 | Metastases to soft tissue | 1 | 0.0768 |
| Mucocutaneous rash | 1 | 0.0679 | Cardiotoxicity | 1 | 0.316 |
| Mucous membrane disorder | 1 | 0.171 | Abdominal tenderness | 1 | 0.244 |
| Skin plaque | 1 | 0.0458 | Haemorrhoidal haemorrhage | 1 | 0.316 |
| Skin wound | 1 | 0.0679 | Diverticulum intestinal | 1 | 0.213 |
| Vasodilatation | 1 | 0.0458 | Oral mucosal eruption | 1 | 0.329 |
| Bone density decreased | 1 | 0.0895 | Cholangitis | 1 | 0.593 |
| Choking sensation | 1 | 0.0232 | Central nervous system lesion | 1 | 0.576 |
| Increased tendency to bruise | 1 | 0.19 | Cerebral disorder | 1 | 0.356 |
| Oesophageal irritation | 1 | 0.0895 | Sneezing | 1 | 0.259 |
| Oesophageal pain | 1 | 0.171 | Intestinal perforation | 1 | 1 |
| Pharyngeal disorder | 1 | 0.171 | Joint effusion | 1 | 0.0768 |
| Pulmonary pain | 1 | 0.374 | Protein urine | 1 | 0.0391 |
| Trichorrhexis | 1 | 0.443 | Arterial thrombosis | 1 | 0.0768 |
| Eyelid oedema | 1 | 0.359 | Osteosclerosis | 1 | 0.113 |
| Oxygen saturation decreased | 1 | 0.618 | Chemical burn | 1 | 0.0581 |
| Thrombotic thrombocytopenic purpura | 1 | 0.151 | Metabolic alkalosis | 1 | 0.0198 |
| Cardiac myxoma | 1 | 0.0232 | Myoclonus | 1 | 0.0768 |
| Acute coronary syndrome | 1 | 0.245 | Angioplasty | 1 | 0.0198 |
| Tumour compression | 1 | 0.131 | Oral fungal infection | 1 | 0.213 |
| Ureteric compression | 1 | 0.0232 | Microcytic anaemia | 1 | 0.0391 |
| Hepatic cancer | 1 | 0.171 | Lip exfoliation | 1 | 0.0581 |
| Pancreatic carcinoma | 1 | 0.151 | Skin texture abnormal | 1 | 0.0768 |
| International normalised ratio fluctuation | 1 | 0.0232 | Gastric varices | 1 | 0.0198 |
| Intentional overdose | 1 | 0.0679 | Splenic vein thrombosis | 1 | 0.0391 |
| Suicide attempt | 1 | 0.111 | Therapy interrupted | 1 | 0.164 |
| Blindness transient | 1 | 0.0679 | Face oedema | 1 | 0.44 |
| Pathological fracture | 1 | 0.245 | Lasegue's test negative | 1 | 0.0198 |
| Feeling of despair | 1 | 0.0458 | Respiratory tract congestion | 1 | 0.164 |
| Imaging procedure abnormal | 1 | 0.0232 | Product use in unapproved indication | 1 | 1 |
| Lacrimal disorder | 1 | 0.0458 | Vomiting projectile | 1 | 0.0391 |
| Lacrimation decreased | 1 | 0.0458 | Thrombophlebitis | 1 | 0.213 |
| Emphysematous cystitis | 1 | 0.0232 | Peritoneal adhesions | 1 | 0.0198 |
| Purpura | 1 | 1 | Hypersensitivity vasculitis | 1 | 0.197 |
| Malnutrition | 1 | 0.417 | Migraine without aura | 1 | 0.0198 |
| Duodenitis | 1 | 0.131 | Brain neoplasm | 1 | 1 |
| Liver abscess | 1 | 0.151 | Malignant hypertension | 1 | 0.0391 |
| Abdominal lymphadenopathy | 1 | 0.0458 | Onycholysis | 1 | 0.462 |
| Atypical pneumonia | 1 | 0.209 | Neoplasm recurrence | 1 | 0.316 |
| Chest X-ray abnormal | 1 | 0.171 | Nodule | 1 | 0.513 |
| Cholestasis | 1 | 0.417 | Diabetic nephropathy | 1 | 0.0198 |
| Cytomegalovirus infection | 1 | 0.111 | Lung cyst | 1 | 0.095 |
| Ileus paralytic | 1 | 0.151 | Blood potassium | 1 | 0.113 |
| Inflammatory marker increased | 1 | 0.111 | Metastases to meninges | 1 | 0.525 |
| Lumbar vertebral fracture | 1 | 0.19 | Gastrointestinal pain | 1 | 0.405 |
| Paralysis | 1 | 0.403 | Clostridial infection | 1 | 0.229 |
| Rectal lesion | 1 | 0.0232 | Blindness transient | 1 | 0.13 |
| Ear swelling | 1 | 0.0232 | Hepatic cancer | 1 | 0.213 |
| Localised infection | 1 | 1 | Ventricular fibrillation | 1 | 0.273 |
| Skin mass | 1 | 0.359 | Cardiac valve disease | 1 | 0.0391 |
| Mucosal dryness | 1 | 0.171 | Renal neoplasm | 1 | 0.148 |
| Acquired haemoglobinopathy | 1 | 0.0232 | Small intestinal haemorrhage | 1 | 0.213 |
| Spinal compression fracture | 1 | 0.43 | Balance disorder | 1 | 1 |
| Prostatic disorder | 1 | 0.111 | Bursitis | 1 | 0.244 |
| Adenomyosis | 1 | 0.0232 | Arthritis bacterial | 1 | 0.0391 |
| Endometrial atrophy | 1 | 0.0232 | Abdominal mass | 1 | 0.13 |
| Hepatic steatosis | 1 | 0.209 | Bone lesion | 1 | 0.405 |
| Leukocyturia | 1 | 0.0232 | Mucosal disorder | 1 | 0.44 |
| Ovarian cyst | 1 | 0.0679 | Cardiopulmonary failure | 1 | 0.197 |
| Ependymoma | 1 | 0.0232 | Renal tubular acidosis | 1 | 0.0391 |
| Hypermagnesaemia | 1 | 0.0232 | Blood urea abnormal | 1 | 0.0581 |
| Hypoperfusion | 1 | 0.0458 | Inappropriate antidiuretic hormone secretion | 1 | 0.259 |
| Depressed level of consciousness | 1 | 0.608 | Breath sounds abnormal | 1 | 0.428 |
| Hepatic mass | 1 | 0.151 | Vulvovaginal pain | 1 | 0.148 |
| Hypersensitivity vasculitis | 1 | 0.263 | Physical deconditioning | 1 | 0.0198 |
| Abnormal faeces | 1 | 0.227 | Pneumonitis aspiration | 1 | 0.0391 |
| Breast disorder | 1 | 0.0458 | Hypercapnia | 1 | 0.095 |
| Impaired healing | 1 | 1 | Generalised tonic-clonic seizure | 1 | 0.343 |
| Laceration | 1 | 0.516 | Blood chloride increased | 1 | 0.13 |
| Lip exfoliation | 1 | 0.0232 | Anaphylactic reaction | 1 | 0.259 |
| Skin texture abnormal | 1 | 0.0232 | Flushing | 1 | 1 |
| Wound haemorrhage | 1 | 0.111 | Acid peptic disease | 1 | 0.0198 |
| Pyoderma | 1 | 0.0458 | Lower respiratory tract infection | 1 | 0.44 |
| Gamma-glutamyltransferase increased | 1 | 0.528 | Muscle contractions involuntary | 1 | 0.0581 |
| Rash vesicular | 1 | 0.417 | Self-medication | 1 | 0.0391 |
| Subdiaphragmatic abscess | 1 | 0.0232 | Hair colour changes | 1 | 0.601 |
| Benign genitourinary tract neoplasm | 1 | 0.0232 | Ear swelling | 1 | 0.0581 |
| Scleral discolouration | 1 | 0.0458 | Skin mass | 1 | 0.288 |
| Food intolerance | 1 | 0.227 | Oesophageal fistula | 1 | 0.0391 |
| Gastrointestinal infection | 1 | 0.171 | Product use issue | 1 | 1 |
| Nasal ulcer | 1 | 0.57 | Musculoskeletal pain | 1 | 0.726 |
| Rectal ulcer | 1 | 0.131 | Atrioventricular block first degree | 1 | 0.0768 |
| Urinary tract infection bacterial | 1 | 0.0232 | Bacterial test positive | 1 | 0.148 |
| Poor quality sleep | 1 | 0.344 | Gastrointestinal necrosis | 1 | 0.197 |
| Escherichia urinary tract infection | 1 | 0.0895 | Pneumonia klebsiella | 1 | 0.0768 |
| Incontinence | 1 | 0.151 | Post procedural haemorrhage | 1 | 0.095 |
| Nasal crusting | 1 | 0.313 | Streptococcal infection | 1 | 0.148 |
| Feeling of body temperature change | 1 | 0.111 | Wound infection | 1 | 0.197 |
| Respiratory acidosis | 1 | 0.0895 | Hernia | 1 | 0.316 |
| Dementia Alzheimer's type | 1 | 0.111 | Hyperplasia | 1 | 0.0391 |
| Erosive duodenitis | 1 | 0.0232 | Urinary bladder haemorrhage | 1 | 0.164 |
| Necrotising oesophagitis | 1 | 0.0232 | Scrotal swelling | 1 | 0.0391 |
| Vascular compression | 1 | 0.0232 | Nasal discomfort | 1 | 1 |
| Colitis ulcerative | 1 | 0.151 | Metastases to thyroid | 1 | 0.164 |
| Urinary tract infection enterococcal | 1 | 0.0232 | Pulmonary granuloma | 1 | 0.0391 |
| Abnormal loss of weight | 1 | 0.296 | Thyroid neoplasm | 1 | 0.0198 |
| Head and neck cancer metastatic | 1 | 0.0232 | Lip pain | 1 | 0.493 |
| Prostate cancer | 1 | 0.313 | Painful respiration | 1 | 0.164 |
| Infected dermal cyst | 1 | 0.0232 | Genital herpes | 1 | 0.0768 |
| Metastases to lymph nodes | 1 | 1 | Rectal abscess | 1 | 0.113 |
| Salivary hypersecretion | 1 | 0.359 | Proctitis | 1 | 0.181 |
| Teeth brittle | 1 | 0.0679 | Synovial cyst | 1 | 0.0581 |
| Tooth fracture | 1 | 0.111 | Jaundice cholestatic | 1 | 0.288 |
| Blastocystis infection | 1 | 0.0232 | Sciatica | 1 | 0.181 |
| Gastric haemorrhage | 1 | 0.359 | Ventricular hypokinesia | 1 | 0.13 |
| Pathogen resistance | 1 | 0.0458 | Blood urine | 1 | 0.0581 |
| Feeling hot | 1 | 1 | Inguinal hernia | 1 | 0.095 |
| Small cell lung cancer | 1 | 0.481 | Enterobacter pneumonia | 1 | 0.0198 |
| Antineutrophil cytoplasmic antibody increased | 1 | 0.0232 | Neutrophilia | 1 | 0.113 |
| Gait inability | 1 | 0.313 | Horner's syndrome | 1 | 0.0391 |
| Venous thrombosis limb | 1 | 0.344 | Ischaemic hepatitis | 1 | 0.0198 |
| Cystitis haemorrhagic | 1 | 0.0895 | Peroneal nerve palsy | 1 | 0.148 |
| Diverticulitis | 1 | 0.505 | Wound haemorrhage | 1 | 0.095 |
| Cardio-respiratory arrest | 1 | 1 | Dermatitis diaper | 1 | 0.095 |
| Lower respiratory tract infection | 1 | 0.359 | Mycobacterium chelonae infection | 1 | 0.0198 |
| Haematocrit decreased | 1 | 0.19 | Sluggishness | 1 | 0.095 |
| Red blood cell count decreased | 1 | 0.43 | Spinal pain | 1 | 0.368 |
| Movement disorder | 1 | 1 | Foot deformity | 1 | 0.095 |
| Renal tubular injury | 1 | 0.0232 | Facial pain | 1 | 0.472 |
| Foreign body sensation in eyes | 1 | 0.209 | Renal cancer | 1 | 0.148 |
| Panophthalmitis | 1 | 0.0232 | Rhinitis allergic | 1 | 0.113 |
| Arterial haemorrhage | 1 | 0.0458 | Sinus operation | 1 | 0.164 |
| Device dislocation | 1 | 0.0232 | Haematospermia | 1 | 0.0581 |
| Vascular occlusion | 1 | 0.131 | Angina pectoris | 1 | 0.343 |
| Choking | 1 | 0.245 | Blood pressure measurement | 1 | 0.0581 |
| Feeding tube user | 1 | 0.0232 | Upper limb fracture | 1 | 0.368 |
| Product administration error | 1 | 0.0232 | Serum ferritin increased | 1 | 0.0198 |
| Wrong technique in product usage process | 1 | 0.493 | Stent placement | 1 | 0.095 |
| Grip strength decreased | 1 | 0.0895 | Phobia | 1 | 0.0198 |
| Lid margin discharge | 1 | 0.0232 | Clubbing | 1 | 0.0391 |
| Skin laceration | 1 | 0.28 | Strangury | 1 | 0.0198 |
| Stevens-Johnson syndrome | 1 | 0.58 | Hypertransaminasaemia | 1 | 1 |
| Bronchospasm | 1 | 0.209 | Spinal fracture | 1 | 0.356 |
| Muscle disorder | 1 | 0.131 | Food poisoning | 1 | 0.148 |
| Upper respiratory tract congestion | 1 | 0.0232 | Gangrene | 1 | 0.0581 |
| Pulmonary nocardiosis | 1 | 0.0458 | Fibromyalgia | 1 | 0.13 |
| Plasma cell myeloma | 1 | 0.131 | Cervix carcinoma recurrent | 1 | 0.0198 |
| Blood calcium decreased | 1 | 0.245 | Shock symptom | 1 | 0.0768 |
| Blood cholesterol increased | 1 | 0.131 | Hyperthyroidism | 1 | 0.273 |
| Subcutaneous abscess | 1 | 0.111 | Miliaria | 1 | 0.0581 |
| Aspartate aminotransferase | 1 | 0.0232 | Systemic lupus erythematosus | 1 | 0.113 |
| Breast cancer | 1 | 0.417 | Renal artery stenosis | 1 | 0.0198 |
| Colon cancer | 1 | 0.329 | Aortic valve calcification | 1 | 0.0198 |
| Blood albumin decreased | 1 | 0.313 | Hypertensive crisis | 1 | 0.113 |
| Product use in unapproved indication | 1 | 1 | Vulvovaginal pruritus | 1 | 0.164 |
| Urethritis | 1 | 0.0232 | Vulvovaginal mycotic infection | 1 | 0.095 |
| Duodenal stenosis | 1 | 0.151 | Stress | 1 | 0.393 |
| Oesophageal stenosis | 1 | 0.245 | Carotid artery occlusion | 1 | 0.0581 |
| Oesophageal ulcer | 1 | 0.296 | Cyst | 1 | 0.288 |
| Cervix carcinoma recurrent | 1 | 0.0232 | Pneumonia cryptococcal | 1 | 0.113 |
| Enterococcal infection | 1 | 0.0232 | Cholecystitis acute | 1 | 0.181 |
| Fibromyalgia | 1 | 0.0895 | Thrombocytosis | 1 | 0.113 |
| Intentional product use issue | 1 | 0.524 | Cholestasis | 1 | 0.343 |
| Migraine without aura | 1 | 0.0232 | Depressed mood | 1 | 0.428 |
| Erythema multiforme | 1 | 1 | Varicose vein | 1 | 0.095 |
| Cutaneous vasculitis | 1 | 0.227 | Calculus urinary | 1 | 0.0198 |
| Pulse absent | 1 | 0.0458 | Cardiac asthma | 1 | 0.0198 |
| Wound | 1 | 0.528 | Extrasystoles | 1 | 0.13 |
| Hyperkeratosis | 1 | 0.263 | Myocardial ischaemia | 1 | 0.244 |
| Ventricular extrasystoles | 1 | 0.19 | Necrotising oesophagitis | 1 | 0.0198 |
| Lung adenocarcinoma | 1 | 0.374 | Erosive duodenitis | 1 | 0.0391 |
| Pharyngitis | 1 | 0.296 | Ocular toxicity | 1 | 0.13 |
| Pancreatic enzymes increased | 1 | 0.0232 | Diabetic retinopathy | 1 | 0.0198 |
| Pancreatic enlargement | 1 | 0.0232 | Gamma radiation therapy to prostate | 1 | 0.0198 |
| Stress cardiomyopathy | 1 | 0.245 | Bronchopulmonary disease | 1 | 0.0391 |
| Disease recurrence | 1 | 0.43 | Peritonitis | 1 | 0.559 |
| Radiation pneumonitis | 1 | 0.505 | Antineutrophil cytoplasmic antibody increased | 1 | 0.0198 |
| Postrenal failure | 1 | 0.0232 | Pyuria | 1 | 0.0198 |
| Mucosal disorder | 1 | 0.151 | Occult blood positive | 1 | 0.181 |
| Anaphylactic shock | 1 | 0.19 | Venous thrombosis limb | 1 | 0.259 |
| Chondrocalcinosis | 1 | 0.0232 | Blood lactate dehydrogenase | 1 | 0.0581 |
| Varicose ulceration | 1 | 0.0232 | Blood sodium | 1 | 0.0581 |
| Dry throat | 1 | 0.456 | Haematocrit | 1 | 0.0391 |
| Meningitis aseptic | 1 | 0.0679 | PCO2 | 1 | 0.0198 |
| Chronic gastritis | 1 | 0.171 | Eructation | 1 | 0.585 |
| Limb injury | 1 | 0.58 | Hyperphagia | 1 | 0.0581 |
| Nodule | 1 | 0.389 | Aspartate aminotransferase | 1 | 0.0581 |
| Dyschezia | 1 | 0.131 | Troponin increased | 1 | 0.244 |
| Lymphatic disorder | 1 | 0.0679 | Fistula | 1 | 0.229 |
| Protein urine present | 1 | 0.171 | Hypothermia | 1 | 0.13 |
| Renal adenoma | 1 | 0.0232 | Glossitis | 1 | 0.493 |
| Illness | 1 | 0.359 | Vasculitis necrotising | 1 | 0.0198 |
| Infusion site pain | 1 | 0.0232 | Aggression | 1 | 0.213 |
| Spinal fracture | 1 | 0.374 | Hypernatraemia | 1 | 0.197 |
| Ventricular arrhythmia | 1 | 0.131 | Pulmonary pain | 1 | 0.273 |
| Wolff-Parkinson-White syndrome | 1 | 0.0232 | Oesophageal pain | 1 | 0.316 |
| Wrist fracture | 1 | 0.245 | Oesophageal irritation | 1 | 0.0581 |
| Osteomyelitis | 1 | 0.245 | Increased tendency to bruise | 1 | 0.148 |
| Dental caries | 1 | 0.28 | Choking sensation | 1 | 0.0391 |
| Agitation | 1 | 0.227 | Trichorrhexis | 1 | 0.417 |
| Mobility decreased | 1 | 0.344 | Bone density decreased | 1 | 0.113 |
| Therapy interrupted | 1 | 0.171 | Pharyngeal disorder | 1 | 0.197 |
| Gastrointestinal perforation | 1 | 0.389 | Face injury | 1 | 0.113 |
| Lower gastrointestinal haemorrhage | 1 | 0.131 | Bacterial sepsis | 1 | 0.0768 |
| Complications of transplanted liver | 1 | 0.0232 | Adenocarcinoma pancreas | 1 | 0.0768 |
| Vasculitis necrotising | 1 | 0.0232 | Intra-abdominal haematoma | 1 | 0.0198 |
| Central nervous system lesion | 1 | 1 | Colitis ischaemic | 1 | 0.259 |
| Cerebral disorder | 1 | 0.389 | Feeling of body temperature change | 1 | 0.197 |
| Dysarthria | 1 | 0.56 | Ingrown hair | 1 | 0.0768 |
| Encephalitis | 1 | 0.19 | Therapy partial responder | 1 | 0.593 |
| Muscle contractions involuntary | 1 | 0.0895 | Foreign body sensation in eyes | 1 | 0.213 |
| Pseudomonas infection | 1 | 0.111 | Intentional product misuse | 1 | 1 |
| Pulmonary mass | 1 | 1 | Vasculitis | 1 | 0.356 |
| Spinal cord oedema | 1 | 0.0232 | Carcinoid syndrome | 1 | 0.0198 |
| Chemical burn | 1 | 0.0232 | Oral dysaesthesia | 1 | 0.0768 |
| Left ventricular failure | 1 | 0.171 | Hunger | 1 | 0.164 |
| Mitral valve incompetence | 1 | 0.263 | Peripheral embolism | 1 | 0.13 |
| Product dose omission issue | 1 | 0.59 | Iatrogenic injury | 1 | 0.0391 |
| Pulmonary hypertension | 1 | 0.296 | Ileus paralytic | 1 | 0.273 |
| Micturition urgency | 1 | 0.171 | Catheter site pain | 1 | 0.0768 |
| Tumour marker abnormal | 1 | 0.0679 | Aphthous ulcer | 1 | 1 |
| Induration | 1 | 0.0232 | Pathogen resistance | 1 | 0.0768 |
| Inhibitory drug interaction | 1 | 0.0232 | Rash maculo-papular | 1 | 0.27 |
| Product prescribing error | 1 | 0.0458 | Pulmonary haemorrhage | 1 | 0.617 |
| Musculoskeletal stiffness | 1 | 0.539 | Urogenital haemorrhage | 1 | 0.0198 |
| Skin odour abnormal | 1 | 0.131 | Oral mucosal blistering | 1 | 0.568 |
| Scab | 1 | 1 | Hepatic cyst | 1 | 0.095 |
| Sputum increased | 1 | 0.151 | Thyroid disorder | 1 | 0.273 |
| Sneezing | 1 | 0.227 | International normalised ratio fluctuation | 1 | 0.0198 |
| Micturition frequency decreased | 1 | 0.0232 | Skin cancer metastatic | 1 | 0.0198 |
| Rhinorrhoea | 1 | 0.272 | Bladder hypertrophy | 1 | 0.0198 |
| Tumour marker increased | 1 | 0.528 | Hypertonic bladder | 1 | 0.0768 |
| Circumoral oedema | 1 | 0.0232 | Clostridium colitis | 1 | 0.13 |
| Tongue oedema | 1 | 0.0679 | Diffuse alveolar damage | 1 | 0.259 |
| Marasmus | 1 | 0.0232 | Pancreatic mass | 1 | 0.0581 |
| Coagulation time prolonged | 1 | 0.0679 | Alveolar lung disease | 1 | 0.0581 |
| Synovial cyst | 1 | 0.0458 | Haemolytic anaemia | 1 | 0.229 |
| Hepatic encephalopathy | 1 | 0.171 | Peripheral circulatory failure | 1 | 0.0391 |
| Gamma radiation therapy to prostate | 1 | 0.0232 | Gastrointestinal erosion | 1 | 0.0198 |
| Bronchopulmonary disease | 1 | 0.0232 | Ileal ulcer | 1 | 0.0198 |
| Glomerulonephritis rapidly progressive | 1 | 0.0232 | Vaginal discharge | 1 | 0.113 |
| Infectious pleural effusion | 1 | 0.227 | Exfoliative rash | 1 | 0.417 |
| Anaphylactic reaction | 1 | 0.359 | Cortisol increased | 1 | 0.0198 |
| Flushing | 1 | 0.59 | Pancreatic neoplasm | 1 | 0.113 |
| Cardiotoxicity | 1 | 0.505 | Gout | 1 | 0.302 |
| Bicytopenia | 1 | 0.0679 | Vocal cord paralysis | 1 | 0.381 |
| Orthopnoea | 1 | 0.0458 | Optic atrophy | 1 | 0.0391 |
| Pneumomediastinum | 1 | 0.111 | Upper respiratory tract infection | 1 | 1 |
| Shock haemorrhagic | 1 | 0.131 | Aspartate aminotransferase abnormal | 1 | 0.095 |
| Gastric ulcer haemorrhage | 1 | 0.344 | Blood alkaline phosphatase abnormal | 1 | 0.164 |
| Malignant hypertension | 1 | 0.0232 | Haemoglobin abnormal | 1 | 0.229 |
| Aortic valve calcification | 1 | 0.0232 | Hyperuricaemia | 1 | 0.213 |
| Stress | 1 | 0.389 | Subdiaphragmatic abscess | 1 | 0.0198 |
| Ventricular tachycardia | 1 | 0.359 | Rash vesicular | 1 | 0.513 |
| Renal artery stenosis | 1 | 0.0232 | Heavy menstrual bleeding | 1 | 0.0768 |
| Tremor | 1 | 1 | Skin candida | 1 | 0.095 |
| Systemic lupus erythematosus | 1 | 0.0679 | Blood phosphorus increased | 1 | 0.0581 |
| Vulvovaginal pruritus | 1 | 0.171 | Urine abnormality | 1 | 0.113 |
| Aortic arteriosclerosis | 1 | 0.151 | Hydroureter | 1 | 0.0198 |
| Vulvovaginal mycotic infection | 1 | 0.0679 | Agranulocytosis | 1 | 0.316 |
| Hypertensive crisis | 1 | 0.0895 | Blood bicarbonate decreased | 1 | 0.0391 |
| Cyst | 1 | 0.245 | Platelet count increased | 1 | 0.343 |
| Carotid artery occlusion | 1 | 0.0458 | Renal cancer metastatic | 1 | 0.0581 |
| Functional gastrointestinal disorder | 1 | 0.19 | Hepatic vein thrombosis | 1 | 0.0391 |
| Therapeutic response decreased | 1 | 0.43 | Hepatic infarction | 1 | 0.0391 |
| Glomerulonephritis minimal lesion | 1 | 0.0232 | Polyp | 1 | 0.095 |
| Myocarditis | 1 | 0.344 | Hip arthroplasty | 1 | 0.13 |
| Face oedema | 1 | 0.313 | Scleral discolouration | 1 | 0.0581 |
| Catheter site discharge | 1 | 0.0232 | Cellulite | 1 | 0.0198 |
| Treatment noncompliance | 1 | 0.296 | Xerosis | 1 | 1 |
| Psychomotor retardation | 1 | 0.0679 | Ocular discomfort | 1 | 0.273 |
| Neurotoxicity | 1 | 0.171 | Nasal ulcer | 1 | 0.593 |
| General physical condition abnormal | 1 | 0.171 | Urinary tract infection bacterial | 1 | 0.0391 |
| Myelosuppression | 1 | 0.27 | International normalised ratio abnormal | 1 | 0.164 |
| Nodal arrhythmia | 1 | 0.0232 | Ejaculation disorder | 1 | 0.0198 |
| Arrhythmia supraventricular | 1 | 0.0458 | Blood pressure systolic decreased | 1 | 0.0768 |
| Peripheral motor neuropathy | 1 | 0.0458 | Pericardial drainage | 1 | 0.0198 |
| Ischaemia | 1 | 0.151 | Oxygen saturation decreased | 1 | 1 |
| Peripheral sensory neuropathy | 1 | 0.151 | Drug-induced liver injury | 1 | 1 |
| Drug hypersensitivity | 1 | 1 | Tongue discolouration | 1 | 0.316 |
| Blood uric acid increased | 1 | 0.131 | Oesophageal obstruction | 1 | 0.0768 |
| Dizziness postural | 1 | 0.131 | Gastrointestinal perforation | 1 | 0.576 |
| Cardiac dysfunction | 1 | 0.443 | Enteritis necroticans | 1 | 0.0198 |
| Kidney infection | 1 | 0.313 | Gastritis haemorrhagic | 1 | 0.113 |
| Blood creatine abnormal | 1 | 0.0232 | RET gene mutation | 1 | 0.0198 |
| Autoimmune hepatitis | 1 | 0.0679 | Muscle fatigue | 1 | 0.0581 |
|  |  |  | Muscle strength abnormal | 1 | 0.0198 |
|  |  |  | Myositis | 1 | 0.288 |
|  |  |  | Panniculitis | 1 | 0.0581 |
|  |  |  | Coordination abnormal | 1 | 0.244 |
|  |  |  | Cortisol decreased | 1 | 0.0391 |
|  |  |  | Enzyme level increased | 1 | 0.0391 |
|  |  |  | Biliary dilatation | 1 | 0.13 |
|  |  |  | Hypersensitivity | 1 | 0.525 |
|  |  |  | Nosocomial infection | 1 | 0.095 |
|  |  |  | Complication associated with device | 1 | 0.393 |
|  |  |  | Therapy change | 1 | 0.302 |
|  |  |  | Blood pressure diastolic decreased | 1 | 0.13 |
|  |  |  | Computerised tomogram abdomen abnormal | 1 | 0.0198 |
|  |  |  | Nasal crusting | 1 | 0.368 |
|  |  |  | Ammonia increased | 1 | 0.113 |
|  |  |  | Affective disorder | 1 | 0.0391 |
|  |  |  | Blood pressure systolic increased | 1 | 0.181 |
|  |  |  | Bladder disorder | 1 | 0.164 |
|  |  |  | Prostatic haemorrhage | 1 | 0.0198 |
|  |  |  | Urine output increased | 1 | 0.0391 |
|  |  |  | Pharyngeal erythema | 1 | 0.0768 |
|  |  |  | Vaginal prolapse | 1 | 0.0198 |
|  |  |  | Non-small cell lung cancer metastatic | 1 | 0.601 |
|  |  |  | Autoimmune thyroiditis | 1 | 0.0391 |
|  |  |  | Metastases to spine | 1 | 0.727 |
|  |  |  | Acquired oesophageal web | 1 | 0.0391 |
|  |  |  | Nail bed disorder | 1 | 0.244 |
|  |  |  | Ear pain | 1 | 0.343 |
|  |  |  | Thyroid hormones increased | 1 | 0.0198 |
|  |  |  | Internal haemorrhage | 1 | 0.244 |
|  |  |  | Wound secretion | 1 | 0.229 |
|  |  |  | Anti-platelet antibody positive | 1 | 0.0198 |
|  |  |  | Penile swelling | 1 | 0.0391 |
|  |  |  | Kidney enlargement | 1 | 0.0581 |
|  |  |  | Portal vein thrombosis | 1 | 0.0581 |
|  |  |  | Biliary cyst | 1 | 0.0198 |
|  |  |  | Haemochromatosis | 1 | 0.0198 |
|  |  |  | Hamartoma | 1 | 0.0198 |
|  |  |  | Scan abdomen abnormal | 1 | 0.0391 |
|  |  |  | Pulmonary alveolar haemorrhage | 1 | 0.368 |
|  |  |  | Renal hypertrophy | 1 | 0.0198 |
|  |  |  | Diplegia | 1 | 0.0581 |
|  |  |  | Diastolic dysfunction | 1 | 0.113 |
|  |  |  | Delusion | 1 | 0.0581 |
|  |  |  | Weaning failure | 1 | 0.0581 |
|  |  |  | Dyskinesia | 1 | 0.302 |
|  |  |  | Metastases to bone marrow | 1 | 0.181 |
|  |  |  | Metastases to lymph nodes | 1 | 1 |
|  |  |  | Prostate cancer metastatic | 1 | 0.0198 |
|  |  |  | Diabetes mellitus inadequate control | 1 | 0.164 |
|  |  |  | Muscle twitching | 1 | 0.244 |
|  |  |  | Postoperative renal failure | 1 | 0.0198 |
|  |  |  | Subdural haematoma | 1 | 0.316 |
|  |  |  | Subdural hygroma | 1 | 0.0581 |
|  |  |  | Eczema asteatotic | 1 | 0.13 |
|  |  |  | Photosensitivity reaction | 1 | 1 |
|  |  |  | Oesophagitis | 1 | 1 |
|  |  |  | Unevaluable event | 1 | 0.55 |
|  |  |  | Disease recurrence | 1 | 0.593 |
|  |  |  | Body temperature decreased | 1 | 0.229 |
|  |  |  | Atrial flutter | 1 | 0.356 |
|  |  |  | Beta haemolytic streptococcal infection | 1 | 0.0391 |
|  |  |  | Embolic stroke | 1 | 0.229 |
|  |  |  | Meningitis | 1 | 0.273 |
|  |  |  | Areflexia | 1 | 0.095 |
|  |  |  | Blood creatine phosphokinase BB increased | 1 | 0.0198 |
|  |  |  | Cardiogenic shock | 1 | 0.259 |
|  |  |  | Pulseless electrical activity | 1 | 0.113 |
|  |  |  | Hyperphosphataemia | 1 | 0.0391 |
|  |  |  | Mallory-Weiss syndrome | 1 | 0.0581 |
|  |  |  | Nutritional condition abnormal | 1 | 0.0581 |
|  |  |  | Tooth loss | 1 | 0.197 |
|  |  |  | Glucose tolerance impaired | 1 | 0.113 |
|  |  |  | Acute myeloid leukaemia | 1 | 0.329 |
|  |  |  | Oesophageal disorder | 1 | 0.213 |
|  |  |  | Cardiomyopathy | 1 | 0.576 |
|  |  |  | Haemorrhage intracranial | 1 | 0.302 |
|  |  |  | Pneumonia pseudomonal | 1 | 0.113 |
|  |  |  | Ecchymosis | 1 | 0.273 |
|  |  |  | Cytomegalovirus test | 1 | 0.0198 |
|  |  |  | COVID-19 pneumonia | 1 | 0.181 |
|  |  |  | Bile duct stenosis | 1 | 0.095 |
|  |  |  | COVID-19 | 1 | 1 |
|  |  |  | Poisoning | 1 | 0.0581 |
|  |  |  | Spontaneous bacterial peritonitis | 1 | 0.0198 |
|  |  |  | Restlessness | 1 | 0.329 |
|  |  |  | Hyperkeratosis | 1 | 0.316 |
|  |  |  | Rectal perforation | 1 | 0.0581 |
|  |  |  | Pulse absent | 1 | 0.0768 |
|  |  |  | Cutaneous vasculitis | 1 | 0.44 |
|  |  |  | Wound | 1 | 0.585 |
|  |  |  | Cancer pain | 1 | 0.302 |
|  |  |  | Renal vessel disorder | 1 | 0.0391 |
|  |  |  | Abscess | 1 | 0.462 |
|  |  |  | Abnormal loss of weight | 1 | 0.316 |
|  |  |  | Head and neck cancer metastatic | 1 | 0.0198 |
|  |  |  | Blood sodium increased | 1 | 0.113 |
|  |  |  | Delusional perception | 1 | 0.0198 |
|  |  |  | Pulmonary congestion | 1 | 0.451 |
|  |  |  | Neurological decompensation | 1 | 0.113 |
|  |  |  | Presyncope | 1 | 0.343 |
|  |  |  | Enterobacter infection | 1 | 0.0391 |
|  |  |  | Skin striae | 1 | 0.0198 |
|  |  |  | Nail dystrophy | 1 | 0.44 |
|  |  |  | Adverse event | 1 | 0.493 |
|  |  |  | Tooth fracture | 1 | 0.113 |
|  |  |  | Teeth brittle | 1 | 0.0581 |
|  |  |  | Thirst | 1 | 0.316 |
|  |  |  | Salivary hypersecretion | 1 | 0.316 |
|  |  |  | Ovarian cancer recurrent | 1 | 0.0198 |
|  |  |  | Drug resistance mutation | 1 | 0.213 |
|  |  |  | Blood pressure fluctuation | 1 | 0.302 |
|  |  |  | Imaging procedure abnormal | 1 | 0.0391 |
|  |  |  | Lacrimal disorder | 1 | 0.0391 |
|  |  |  | Feeling of despair | 1 | 0.0581 |
|  |  |  | Liver injury | 1 | 0.213 |
|  |  |  | Lacrimation decreased | 1 | 0.0581 |
|  |  |  | Sleep disorder | 1 | 0.513 |
|  |  |  | Eosinophilia | 1 | 0.164 |
|  |  |  | Liver abscess | 1 | 0.273 |
|  |  |  | Electrocardiogram QT prolonged | 1 | 1 |
|  |  |  | Urinary tract infection enterococcal | 1 | 0.0198 |
|  |  |  | Hepatitis fulminant | 1 | 0.0581 |
