# Supplementary material for: Renal adverse events in EGFR-TKI treatment: Comprehensive characterization of clinical patterns and molecular underpinnings
Source: Genes Dis. 2025 Nov 28;13(4):101953. doi: 10.1016/j.gendis.2025.101953 (PMC12993402; doi:10.1016/j.gendis.2025.101953)
Supplement: Table S6 — The 2 × 2 contingency table, detailed calculation formulas for each disproportionality analysis method, and the predefined positive signal thresholds applied in this study. [file mmc7.docx]

Supplementary Table 6: Four-grid table and formula for ROR and IC calculation used for signal detection.

| Database | Target AEs | All other AEs | Total |
| --- | --- | --- | --- |
| Target drug | a | b | a+b |
| All other drugs | c | d | c+d |
| Total | a+c | b+d | a+b+c+d |

| Algorithms | Equation | Criteria |
| --- | --- | --- |
| ROR | ROR=ad/bc | lower limit of 95% CI>1,  N≥3 |
|  | 95%CI=e^ln(ROR)±1.96(1/a+1/b+1/c+1/d)^0.5^ |  |
| BCPNN | IC=log_2_a(a+b+c+d)/((a+c)(a+b)) | IC_025_>0 |
|  | IC_025_=e^ln(IC)-1.96(1/a+1/b+1/c+1/d)^0.5^ |  |

Abbreviations: BCPNN, Bayesian confidence propagation neural network; CI, confidence interval; EBGM, empirical Bayesian geometric mean; IC, information component; IC025, the lower limit of the 95% two-sided CI of the IC; N, the number of co-occurrences; ROR, reporting odds ratio.

a: number of reports containing both the suspect drug and the suspect adverse drug reaction. b: number of reports containing the suspect adverse drug reaction with other medications (except the drug of interest). c: number of reports containing the suspect drug with other adverse drug reactions (except the event of interest). d: number of reports containing other medications and other adverse drug reactions.
