# Supplementary material for: Renal adverse events in EGFR-TKI treatment: Comprehensive characterization of clinical patterns and molecular underpinnings
Source: Genes Dis. 2025 Nov 28;13(4):101953. doi: 10.1016/j.gendis.2025.101953 (PMC12993402; doi:10.1016/j.gendis.2025.101953)
Supplement: Table S7 — Overall adverse reaction risk signals for EGFR-TKIs in the FAERS and VigiBase database. [file mmc8.docx]

**Supplementary Table 7. Overall adverse reaction risk signals for EGFR-TKIs in the FAERS and VigiBase database.**

| **PT** | **ROR** | **Upper limit of the 95% confidence interval for ROR** | **Lower limit of the 95% confidence interval for ROR** | **IC** | **IC025** | **P value** | **Database** |
| --- | --- | --- | --- | --- | --- | --- | --- |
| Prerenal failure | 2.96 | 5.01 | 1.75 | 1.49 | 0.88 | 2.33E-05 | FAERS |
| Azotaemia | 1.73 | 3.27 | 0.92 | 0.77 | 0.41 | 0.09 | FAERS |
| Proteinuria | 0.90 | 1.13 | 0.73 | -0.14 | NA | 0.37 | FAERS |
| Hydronephrosis | 0.66 | 1.08 | 0.40 | -0.59 | NA | 0.1 | FAERS |
| Haematuria | 0.65 | 0.86 | 0.49 | -0.61 | NA | 0 | FAERS |
| Nocturia | 0.63 | 1.08 | 0.37 | -0.64 | NA | 0.09 | FAERS |
| Renal disorder | 0.62 | 0.79 | 0.49 | -0.67 | NA | 6.37E-05 | FAERS |
| Chronic kidney disease | 0.62 | 0.93 | 0.41 | -0.68 | NA | 0.02 | FAERS |
| Renal impairment | 0.61 | 0.73 | 0.51 | -0.70 | NA | 3.93E-08 | FAERS |
| Urine odour abnormal | 0.59 | 1.32 | 0.26 | -0.75 | NA | 0.19 | FAERS |
| Renal failure | 0.57 | 0.67 | 0.48 | -0.80 | NA | 1.78E-11 | FAERS |
| Acute kidney injury | 0.57 | 0.66 | 0.49 | -0.79 | NA | 7.25E-15 | FAERS |
| Chromaturia | 0.54 | 0.87 | 0.34 | -0.86 | NA | 0.01 | FAERS |
| Renal injury | 0.50 | 1.22 | 0.21 | -0.97 | NA | 0.12 | FAERS |
| Oliguria | 0.49 | 1.52 | 0.16 | -1.02 | NA | 0.21 | FAERS |
| Renal tubular necrosis | 0.41 | 1.09 | 0.15 | -1.27 | NA | 0.06 | FAERS |
| Nephropathy | 0.40 | 1.08 | 0.15 | -1.29 | NA | 0.06 | FAERS |
| Nephrotic syndrome | 0.39 | 0.81 | 0.18 | -1.35 | NA | 0.01 | FAERS |
| Anuria | 0.31 | 0.97 | 0.10 | -1.65 | NA | 0.03 | FAERS |
| Renal pain | 0.27 | 0.73 | 0.10 | -1.84 | NA | 0.01 | FAERS |
| Nephritis | 0.21 | 0.67 | 0.07 | -2.19 | NA | 0 | FAERS |
| Tubulointerstitial nephritis | 0.13 | 0.42 | 0.04 | -2.86 | NA | 4.62E-05 | FAERS |
| Prerenal failure | 3.02 | 5.57 | 1.64 | 1.53 | 0.83 | 1.97E-04 | VigiBase |
| Chromaturia | 1.03 | 1.38 | 0.76 | 0.04 | 0.03 | 0.87 | VigiBase |
| Acute kidney injury | 0.81 | 0.90 | 0.73 | -0.30 | NA | 1.17E-04 | VigiBase |
| Hydronephrosis | 0.77 | 1.17 | 0.50 | -0.37 | NA | 0.22 | VigiBase |
| Renal failure | 0.70 | 0.79 | 0.62 | -0.50 | NA | 2.40E-08 | VigiBase |
| Proteinuria | 0.68 | 0.87 | 0.53 | -0.54 | NA | 0 | VigiBase |
| Haematuria | 0.67 | 0.85 | 0.53 | -0.56 | NA | 0 | VigiBase |
| Renal disorder | 0.65 | 0.81 | 0.52 | -0.61 | NA | 8.67E-05 | VigiBase |
| Azotaemia | 0.64 | 1.23 | 0.33 | -0.64 | NA | 0.18 | VigiBase |
| Nocturia | 0.61 | 0.99 | 0.38 | -0.69 | NA | 0.04 | VigiBase |
| Anuria | 0.59 | 1.24 | 0.28 | -0.76 | NA | 0.16 | VigiBase |
| Urine odour abnormal | 0.58 | 1.22 | 0.27 | -0.78 | NA | 0.14 | VigiBase |
| Renal impairment | 0.54 | 0.65 | 0.46 | -0.86 | NA | 5.94E-12 | VigiBase |
| Chronic kidney disease | 0.50 | 0.72 | 0.34 | -0.99 | NA | 1.84E-04 | VigiBase |
| Nephropathy | 0.41 | 0.99 | 0.17 | -1.27 | NA | 0.04 | VigiBase |
| Nephrotic syndrome | 0.41 | 0.76 | 0.22 | -1.28 | NA | 0 | VigiBase |
| Renal injury | 0.40 | 1.07 | 0.15 | -1.30 | NA | 0.06 | VigiBase |
| Renal pain | 0.33 | 0.70 | 0.16 | -1.56 | NA | 0 | VigiBase |
| Oliguria | 0.30 | 0.92 | 0.10 | -1.73 | NA | 0.03 | VigiBase |
| Renal tubular necrosis | 0.30 | 0.72 | 0.12 | -1.71 | NA | 0 | VigiBase |
| Tubulointerstitial nephritis | 0.20 | 0.45 | 0.09 | -2.28 | NA | 1.47E-05 | VigiBase |
