# Supplementary material for: Renal adverse events in EGFR-TKI treatment: Comprehensive characterization of clinical patterns and molecular underpinnings
Source: Genes Dis. 2025 Nov 28;13(4):101953. doi: 10.1016/j.gendis.2025.101953 (PMC12993402; doi:10.1016/j.gendis.2025.101953)
Supplement: Table S8 — EGFR-TKIs renal adverse reaction risk signals from FAERS and VigiBase databases. [file mmc9.docx]

**Supplementary Table 8. EGFR-TKIs renal adverse reaction risk signals from FAERS and VigiBase databases.**

| **PT** | **ROR** | **Upper limit of the 95% confidence interval for ROR** | **Lower limit of the 95% confidence interval for ROR** | **IC** | **IC025** | **P value** | **Drug** | **Database** |
| --- | --- | --- | --- | --- | --- | --- | --- | --- |
| Prerenal failure | 13.96 | 25.05 | 7.78 | 3.72 | 2.07 | 2.96E-31 | Afatinib | FAERS |
| Azotaemia | 4.04 | 10.87 | 1.50 | 1.99 | 0.74 | 0 | Afatinib | FAERS |
| Proteinuria | 1.35 | 1.73 | 1.06 | 0.43 | 0.34 | 0.01 | Erlotinib | FAERS |
| Acute kidney injury | 1.33 | 1.67 | 1.06 | 0.40 | 0.32 | 0.02 | Afatinib | FAERS |
| Chromaturia | 1.80 | 3.36 | 0.97 | 0.84 | 0.45 | 0.06 | Afatinib | FAERS |
| Renal impairment | 1.12 | 1.49 | 0.84 | 0.16 | 0.12 | 0.44 | Osimertinib | FAERS |
| Acute kidney injury | 1.79 | 4.32 | 0.74 | 0.84 | 0.35 | 0.19 | Dacomitinib | FAERS |
| Renal disorder | 1.04 | 1.61 | 0.67 | 0.05 | 0.03 | 0.87 | Afatinib | FAERS |
| Hydronephrosis | 2.03 | 6.31 | 0.65 | 1.02 | 0.33 | 0.21 | Gefitinib | FAERS |
| Renal failure | 0.82 | 1.15 | 0.59 | -0.28 | NA | 0.25 | Afatinib | FAERS |
| Haematuria | 0.73 | 1.04 | 0.51 | -0.45 | NA | 0.08 | Erlotinib | FAERS |
| Renal impairment | 0.90 | 1.62 | 0.50 | -0.16 | NA | 0.72 | Gefitinib | FAERS |
| Proteinuria | 1.09 | 2.43 | 0.49 | 0.13 | 0.06 | 0.83 | Gefitinib | FAERS |
| Renal injury | 1.46 | 4.54 | 0.47 | 0.54 | 0.17 | 0.51 | Osimertinib | FAERS |
| Azotaemia | 1.24 | 3.35 | 0.46 | 0.31 | 0.12 | 0.66 | Erlotinib | FAERS |
| Renal disorder | 0.71 | 1.15 | 0.44 | -0.48 | NA | 0.16 | Osimertinib | FAERS |
| Haematuria | 0.76 | 1.34 | 0.43 | -0.39 | NA | 0.34 | Osimertinib | FAERS |
| Chronic kidney disease | 0.92 | 2.04 | 0.41 | -0.13 | NA | 0.83 | Afatinib | FAERS |
| Renal impairment | 0.62 | 0.94 | 0.40 | -0.70 | NA | 0.02 | Afatinib | FAERS |
| Renal failure | 0.57 | 0.82 | 0.39 | -0.81 | NA | 0 | Osimertinib | FAERS |
| Renal failure | 0.50 | 0.64 | 0.39 | -0.99 | NA | 8.52E-09 | Erlotinib | FAERS |
| Renal disorder | 0.50 | 0.71 | 0.35 | -1.00 | NA | 7.44E-05 | Erlotinib | FAERS |
| Acute kidney injury | 0.48 | 0.67 | 0.34 | -1.06 | NA | 1.33E-05 | Osimertinib | FAERS |
| Chronic kidney disease | 0.74 | 1.65 | 0.33 | -0.43 | NA | 0.46 | Osimertinib | FAERS |
| Renal failure | 0.60 | 1.16 | 0.31 | -0.73 | NA | 0.13 | Gefitinib | FAERS |
| Acute kidney injury | 0.39 | 0.49 | 0.31 | -1.34 | NA | 2.68E-16 | Erlotinib | FAERS |
| Hydronephrosis | 0.60 | 1.21 | 0.30 | -0.72 | NA | 0.15 | Erlotinib | FAERS |
| Renal impairment | 0.40 | 0.54 | 0.30 | -1.31 | NA | 2.83E-10 | Erlotinib | FAERS |
| Nocturia | 0.58 | 1.22 | 0.28 | -0.77 | NA | 0.15 | Erlotinib | FAERS |
| Urine odour abnormal | 0.72 | 1.93 | 0.27 | -0.46 | NA | 0.51 | Erlotinib | FAERS |
| Nephrotic syndrome | 0.80 | 2.48 | 0.26 | -0.32 | NA | 0.7 | Osimertinib | FAERS |
| Nocturia | 0.80 | 2.50 | 0.26 | -0.31 | NA | 0.71 | Afatinib | FAERS |
| Chronic kidney disease | 0.47 | 0.88 | 0.25 | -1.07 | NA | 0.02 | Erlotinib | FAERS |
| Haematuria | 0.65 | 2.02 | 0.21 | -0.62 | NA | 0.46 | Gefitinib | FAERS |
| Hydronephrosis | 0.59 | 1.83 | 0.19 | -0.76 | NA | 0.36 | Osimertinib | FAERS |
| Proteinuria | 0.37 | 0.78 | 0.18 | -1.43 | NA | 0.01 | Osimertinib | FAERS |
| Chromaturia | 0.39 | 0.81 | 0.18 | -1.36 | NA | 0.01 | Erlotinib | FAERS |
| Renal tubular necrosis | 0.56 | 1.75 | 0.18 | -0.82 | NA | 0.31 | Erlotinib | FAERS |
| Acute kidney injury | 0.30 | 0.66 | 0.13 | -1.75 | NA | 0 | Gefitinib | FAERS |
| Haematuria | 0.31 | 0.83 | 0.12 | -1.67 | NA | 0.01 | Afatinib | FAERS |
| Nephrotic syndrome | 0.30 | 0.94 | 0.10 | -1.70 | NA | 0.03 | Erlotinib | FAERS |

| **PT** | **ROR** | **Upper limit of the 95% confidence interval for ROR** | **Lower limit of the 95% confidence interval for ROR** | **IC** | **IC025** | **P value** | **Drug** | **Database** |
| --- | --- | --- | --- | --- | --- | --- | --- | --- |
| Prerenal failure | 17.63 | 34.53 | 9.00 | 4.06 | 2.07 | 7.25E-31 | Afatinib | VigiBase |
| Chromaturia | 2.57 | 4.79 | 1.38 | 1.36 | 0.73 | 0 | Gefitinib | VigiBase |
| Haematuria | 2.06 | 3.24 | 1.31 | 1.04 | 0.66 | 0 | Gefitinib | VigiBase |
| Acute kidney injury | 1.55 | 1.91 | 1.27 | 0.63 | 0.51 | 2.25E-05 | Afatinib | VigiBase |
| Chromaturia | 1.99 | 3.51 | 1.13 | 0.99 | 0.56 | 0.02 | Afatinib | VigiBase |
| Renal disorder | 1.18 | 1.80 | 0.77 | 0.23 | 0.15 | 0.46 | Afatinib | VigiBase |
| Azotaemia | 2.37 | 7.36 | 0.76 | 1.24 | 0.40 | 0.12 | Gefitinib | VigiBase |
| Renal failure | 0.99 | 1.32 | 0.75 | -0.01 | NA | 0.97 | Afatinib | VigiBase |
| Acute kidney injury | 0.99 | 1.37 | 0.72 | -0.01 | NA | 0.97 | Gefitinib | VigiBase |
| Proteinuria | 0.94 | 1.23 | 0.72 | -0.09 | NA | 0.66 | Erlotinib | VigiBase |
| Renal impairment | 0.93 | 1.34 | 0.65 | -0.10 | NA | 0.71 | Afatinib | VigiBase |
| Acute kidney injury | 0.73 | 0.84 | 0.63 | -0.45 | NA | 1.11E-05 | Erlotinib | VigiBase |
| Renal failure | 0.69 | 0.81 | 0.59 | -0.52 | NA | 6.46E-06 | Erlotinib | VigiBase |
| Hydronephrosis | 1.55 | 4.14 | 0.58 | 0.63 | 0.24 | 0.38 | Gefitinib | VigiBase |
| Nephropathy | 1.77 | 5.49 | 0.57 | 0.82 | 0.26 | 0.32 | Afatinib | VigiBase |
| Renal impairment | 0.79 | 1.15 | 0.54 | -0.34 | NA | 0.21 | Osimertinib | VigiBase |
| Chromaturia | 0.83 | 1.26 | 0.54 | -0.27 | NA | 0.38 | Erlotinib | VigiBase |
| Hydronephrosis | 0.85 | 1.42 | 0.51 | -0.23 | NA | 0.54 | Erlotinib | VigiBase |
| Proteinuria | 0.93 | 1.87 | 0.47 | -0.10 | NA | 0.84 | Gefitinib | VigiBase |
| Renal disorder | 0.87 | 1.62 | 0.47 | -0.20 | NA | 0.66 | Gefitinib | VigiBase |
| Renal failure | 0.69 | 1.05 | 0.46 | -0.53 | NA | 0.08 | Gefitinib | VigiBase |
| Nephrotic syndrome | 1.36 | 4.22 | 0.44 | 0.44 | 0.14 | 0.59 | Gefitinib | VigiBase |
| Haematuria | 0.55 | 0.77 | 0.40 | -0.85 | NA | 3.98E-04 | Erlotinib | VigiBase |
| Renal disorder | 0.52 | 0.71 | 0.38 | -0.93 | NA | 2.17E-05 | Erlotinib | VigiBase |
| Chronic kidney disease | 0.58 | 0.90 | 0.37 | -0.78 | NA | 0.01 | Erlotinib | VigiBase |
| Nocturia | 0.97 | 2.58 | 0.36 | -0.05 | NA | 0.94 | Osimertinib | VigiBase |
| Renal failure | 0.49 | 0.72 | 0.34 | -1.02 | NA | 2.38E-04 | Osimertinib | VigiBase |
| Renal disorder | 0.58 | 1.04 | 0.32 | -0.79 | NA | 0.06 | Osimertinib | VigiBase |
| Renal impairment | 0.41 | 0.53 | 0.32 | -1.28 | NA | 2.15E-12 | Erlotinib | VigiBase |
| Renal impairment | 0.53 | 0.96 | 0.29 | -0.91 | NA | 0.03 | Gefitinib | VigiBase |
| Urine odour abnormal | 0.67 | 1.62 | 0.28 | -0.57 | NA | 0.37 | Erlotinib | VigiBase |
| Haematuria | 0.56 | 1.11 | 0.28 | -0.84 | NA | 0.09 | Afatinib | VigiBase |
| Acute kidney injury | 0.39 | 0.58 | 0.27 | -1.34 | NA | 1.29E-06 | Osimertinib | VigiBase |
| Nocturia | 0.53 | 1.01 | 0.27 | -0.92 | NA | 0.05 | Erlotinib | VigiBase |
| Chronic kidney disease | 0.64 | 1.53 | 0.26 | -0.65 | NA | 0.31 | Afatinib | VigiBase |
| Nocturia | 0.77 | 2.40 | 0.25 | -0.37 | NA | 0.65 | Afatinib | VigiBase |
| Haematuria | 0.46 | 0.96 | 0.22 | -1.13 | NA | 0.03 | Osimertinib | VigiBase |
| Acute kidney injury | 0.69 | 2.15 | 0.22 | -0.53 | NA | 0.52 | Dacomitinib | VigiBase |
| Anuria | 0.54 | 1.45 | 0.20 | -0.87 | NA | 0.22 | Erlotinib | VigiBase |
| Renal pain | 0.39 | 0.94 | 0.16 | -1.35 | NA | 0.03 | Erlotinib | VigiBase |
| Proteinuria | 0.35 | 0.84 | 0.15 | -1.51 | NA | 0.01 | Osimertinib | VigiBase |
| Renal tubular necrosis | 0.39 | 1.04 | 0.15 | -1.34 | NA | 0.05 | Erlotinib | VigiBase |
| Azotaemia | 0.34 | 1.07 | 0.11 | -1.53 | NA | 0.05 | Erlotinib | VigiBase |
| Nephrotic syndrome | 0.26 | 0.70 | 0.10 | -1.91 | NA | 0 | Erlotinib | VigiBase |
| Tubulointerstitial nephritis | 0.16 | 0.51 | 0.05 | -2.58 | NA | 3.71E-04 | Erlotinib | VigiBase |
