# Supplementary material for: Renal adverse events in EGFR-TKI treatment: Comprehensive characterization of clinical patterns and molecular underpinnings
Source: Genes Dis. 2025 Nov 28;13(4):101953. doi: 10.1016/j.gendis.2025.101953 (PMC12993402; doi:10.1016/j.gendis.2025.101953)
Supplement: Table S9 — Comparison of adverse reaction risk signals among five EGFR-TKIs. [file mmc10.docx]

**Supplementary Table 9. Comparing adverse reaction risk signals among five EGFR-TKIs.**

| **PT** | **ROR** | **Upper limit of the 95% confidence interval for ROR** | **Lower limit of the 95% confidence interval for ROR** | **IC** | **IC025** | **P value** | **Drug** | **Database** |
| --- | --- | --- | --- | --- | --- | --- | --- | --- |
| Prerenal failure | 19.45 | 68.93 | 5.49 | 2.23 | 0.63 | 9.25E-11 | Afatinib | FAERS |
| Chromaturia | 6.08 | 15.40 | 2.40 | 1.70 | 0.67 | 1.42E-05 | Afatinib | FAERS |
| Acute kidney injury | 3.14 | 4.20 | 2.34 | 1.20 | 0.89 | 6.58E-16 | Afatinib | FAERS |
| Proteinuria | 3.62 | 6.35 | 2.07 | 0.57 | 0.33 | 1.46E-06 | Erlotinib | FAERS |
| Renal impairment | 2.32 | 3.34 | 1.61 | 0.86 | 0.60 | 3.08E-06 | Osimertinib | FAERS |
| Acute kidney injury | 3.17 | 7.72 | 1.30 | 1.63 | 0.67 | 0.01 | Dacomitinib | FAERS |
| Renal disorder | 1.91 | 3.20 | 1.14 | 0.72 | 0.43 | 0.01 | Afatinib | FAERS |
| Renal failure | 1.57 | 2.32 | 1.07 | 0.52 | 0.35 | 0.02 | Afatinib | FAERS |
| Hydronephrosis | 3.52 | 12.37 | 1.00 | 1.61 | 0.46 | 0.04 | Gefitinib | FAERS |
| Renal injury | 5.62 | 33.64 | 0.94 | 1.51 | 0.25 | 0.03 | Osimertinib | FAERS |
| Azotaemia | 3.24 | 11.48 | 0.91 | 1.23 | 0.35 | 0.05 | Afatinib | FAERS |
| Renal impairment | 1.50 | 2.79 | 0.81 | 0.54 | 0.29 | 0.2 | Gefitinib | FAERS |
| Haematuria | 1.30 | 2.31 | 0.73 | 0.16 | 0.09 | 0.37 | Erlotinib | FAERS |
| Renal disorder | 1.18 | 2.04 | 0.68 | 0.18 | 0.11 | 0.55 | Osimertinib | FAERS |
| Renal failure | 0.99 | 1.49 | 0.66 | -0.01 | NA | 0.95 | Osimertinib | FAERS |
| Chronic kidney disease | 1.62 | 4.08 | 0.64 | 0.55 | 0.22 | 0.3 | Afatinib | FAERS |
| Nephrotic syndrome | 2.81 | 12.56 | 0.63 | 1.02 | 0.23 | 0.16 | Osimertinib | FAERS |
| Haematuria | 1.22 | 2.33 | 0.63 | 0.22 | 0.11 | 0.56 | Osimertinib | FAERS |
| Renal impairment | 1.00 | 1.60 | 0.63 | 0.00 | 0.00 | 1 | Afatinib | FAERS |
| Renal failure | 0.77 | 1.07 | 0.55 | -0.19 | NA | 0.11 | Erlotinib | FAERS |
| Renal failure | 1.06 | 2.08 | 0.54 | 0.07 | 0.04 | 0.87 | Gefitinib | FAERS |
| Acute kidney injury | 0.79 | 1.15 | 0.54 | -0.27 | NA | 0.22 | Osimertinib | FAERS |
| Proteinuria | 1.22 | 2.81 | 0.53 | 0.27 | 0.12 | 0.64 | Gefitinib | FAERS |
| Chronic kidney disease | 1.25 | 3.15 | 0.50 | 0.25 | 0.10 | 0.64 | Osimertinib | FAERS |
| Renal disorder | 0.64 | 1.02 | 0.40 | -0.33 | NA | 0.06 | Erlotinib | FAERS |
| Nocturia | 1.33 | 4.75 | 0.37 | 0.33 | 0.09 | 0.66 | Afatinib | FAERS |
| Acute kidney injury | 0.49 | 0.66 | 0.37 | -0.55 | NA | 1.73E-06 | Erlotinib | FAERS |
| Renal impairment | 0.46 | 0.66 | 0.32 | -0.62 | NA | 2.07E-05 | Erlotinib | FAERS |
| Haematuria | 1.00 | 3.20 | 0.31 | -0.01 | NA | 0.99 | Gefitinib | FAERS |
| Hydronephrosis | 0.82 | 2.19 | 0.31 | -0.13 | NA | 0.7 | Erlotinib | FAERS |
| Urine odour abnormal | 1.65 | 8.99 | 0.30 | 0.28 | 0.05 | 0.56 | Erlotinib | FAERS |
| Nocturia | 0.82 | 2.35 | 0.29 | -0.13 | NA | 0.71 | Erlotinib | FAERS |
| Renal tubular necrosis | 2.47 | 23.73 | 0.26 | 0.45 | 0.05 | 0.42 | Erlotinib | FAERS |
| Chronic kidney disease | 0.59 | 1.32 | 0.26 | -0.40 | NA | 0.19 | Erlotinib | FAERS |
| Hydronephrosis | 0.86 | 3.03 | 0.25 | -0.17 | NA | 0.82 | Osimertinib | FAERS |
| Acute kidney injury | 0.50 | 1.13 | 0.22 | -0.95 | NA | 0.09 | Gefitinib | FAERS |
| Chromaturia | 0.52 | 1.35 | 0.20 | -0.50 | NA | 0.17 | Erlotinib | FAERS |
| Proteinuria | 0.35 | 0.77 | 0.16 | -1.29 | NA | 0.01 | Osimertinib | FAERS |
| Haematuria | 0.43 | 1.20 | 0.16 | -1.06 | NA | 0.1 | Afatinib | FAERS |
| Azotaemia | 0.55 | 1.94 | 0.15 | -0.46 | NA | 0.34 | Erlotinib | FAERS |
| Nephrotic syndrome | 0.62 | 2.76 | 0.14 | -0.36 | NA | 0.52 | Erlotinib | FAERS |

| **PT** | **ROR** | **Upper limit of the 95% confidence interval for ROR** | **Lower limit of the 95% confidence interval for ROR** | **IC** | **IC025** | **P value** | **Drug** | **Database** |
| --- | --- | --- | --- | --- | --- | --- | --- | --- |
| Prerenal failure | 27.39 | 126.78 | 5.92 | 2.54 | 0.55 | 1.13E-10 | Afatinib | VigiBase |
| Haematuria | 3.80 | 6.45 | 2.24 | 1.60 | 0.94 | 9.72E-08 | Gefitinib | VigiBase |
| Acute kidney injury | 2.24 | 2.84 | 1.76 | 0.93 | 0.73 | 1.32E-11 | Afatinib | VigiBase |
| Proteinuria | 3.40 | 6.67 | 1.73 | 0.45 | 0.23 | 1.55E-04 | Erlotinib | VigiBase |
| Nephropathy | 9.13 | 54.63 | 1.52 | 2.09 | 0.35 | 0 | Afatinib | VigiBase |
| Chromaturia | 2.94 | 5.96 | 1.45 | 1.32 | 0.65 | 0 | Gefitinib | VigiBase |
| Renal impairment | 1.92 | 2.90 | 1.28 | 0.77 | 0.51 | 0 | Afatinib | VigiBase |
| Renal disorder | 2.06 | 3.38 | 1.26 | 0.84 | 0.51 | 0 | Afatinib | VigiBase |
| Azotaemia | 5.00 | 20.00 | 1.25 | 1.87 | 0.47 | 0.01 | Gefitinib | VigiBase |
| Chromaturia | 2.28 | 4.43 | 1.18 | 0.95 | 0.49 | 0.01 | Afatinib | VigiBase |
| Nephrotic syndrome | 4.29 | 16.58 | 1.11 | 1.72 | 0.45 | 0.02 | Gefitinib | VigiBase |
| Renal failure | 1.51 | 2.07 | 1.11 | 0.50 | 0.36 | 0.01 | Afatinib | VigiBase |
| Renal impairment | 1.55 | 2.38 | 1.01 | 0.52 | 0.34 | 0.04 | Osimertinib | VigiBase |
| Acute kidney injury | 1.25 | 1.75 | 0.89 | 0.29 | 0.20 | 0.2 | Gefitinib | VigiBase |
| Hydronephrosis | 2.22 | 6.57 | 0.75 | 1.00 | 0.34 | 0.14 | Gefitinib | VigiBase |
| Renal failure | 0.96 | 1.25 | 0.75 | -0.02 | NA | 0.78 | Erlotinib | VigiBase |
| Renal disorder | 1.37 | 2.65 | 0.71 | 0.41 | 0.21 | 0.35 | Gefitinib | VigiBase |
| Chronic kidney disease | 1.54 | 3.51 | 0.68 | 0.21 | 0.09 | 0.3 | Erlotinib | VigiBase |
| Proteinuria | 1.40 | 2.94 | 0.67 | 0.44 | 0.21 | 0.37 | Gefitinib | VigiBase |
| Renal failure | 0.98 | 1.52 | 0.63 | -0.02 | NA | 0.94 | Gefitinib | VigiBase |
| Acute kidney injury | 0.77 | 0.95 | 0.62 | -0.16 | NA | 0.01 | Erlotinib | VigiBase |
| Nocturia | 1.73 | 5.32 | 0.57 | 0.64 | 0.21 | 0.33 | Osimertinib | VigiBase |
| Hydronephrosis | 1.32 | 3.25 | 0.54 | 0.14 | 0.06 | 0.54 | Erlotinib | VigiBase |
| Renal impairment | 0.97 | 1.79 | 0.52 | -0.05 | NA | 0.91 | Gefitinib | VigiBase |
| Chronic kidney disease | 1.32 | 3.48 | 0.50 | 0.34 | 0.13 | 0.57 | Afatinib | VigiBase |
| Renal disorder | 0.86 | 1.62 | 0.46 | -0.19 | NA | 0.64 | Osimertinib | VigiBase |
| Renal failure | 0.67 | 1.00 | 0.44 | -0.51 | NA | 0.05 | Osimertinib | VigiBase |
| Haematuria | 0.64 | 1.02 | 0.40 | -0.29 | NA | 0.06 | Erlotinib | VigiBase |
| Renal disorder | 0.60 | 0.93 | 0.39 | -0.32 | NA | 0.02 | Erlotinib | VigiBase |
| Renal impairment | 0.53 | 0.76 | 0.38 | -0.41 | NA | 3.84E-04 | Erlotinib | VigiBase |
| Haematuria | 0.80 | 1.67 | 0.38 | -0.28 | NA | 0.55 | Afatinib | VigiBase |
| Nocturia | 1.30 | 4.54 | 0.37 | 0.32 | 0.09 | 0.68 | Afatinib | VigiBase |
| Chromaturia | 0.62 | 1.12 | 0.34 | -0.31 | NA | 0.11 | Erlotinib | VigiBase |
| Renal pain | 1.54 | 7.96 | 0.30 | 0.21 | 0.04 | 0.6 | Erlotinib | VigiBase |
| Urine odour abnormal | 1.54 | 7.96 | 0.30 | 0.21 | 0.04 | 0.6 | Erlotinib | VigiBase |
| Haematuria | 0.64 | 1.39 | 0.29 | -0.57 | NA | 0.25 | Osimertinib | VigiBase |
| Acute kidney injury | 0.44 | 0.67 | 0.29 | -1.05 | NA | 5.53E-05 | Osimertinib | VigiBase |
| Renal tubular necrosis | 2.47 | 22.10 | 0.28 | 0.37 | 0.04 | 0.4 | Erlotinib | VigiBase |
| Nocturia | 0.69 | 1.80 | 0.27 | -0.22 | NA | 0.45 | Erlotinib | VigiBase |
| Acute kidney injury | 0.85 | 2.65 | 0.27 | -0.23 | NA | 0.78 | Dacomitinib | VigiBase |
| Proteinuria | 0.47 | 1.17 | 0.19 | -0.97 | NA | 0.1 | Osimertinib | VigiBase |
| Anuria | 0.82 | 3.68 | 0.18 | -0.11 | NA | 0.8 | Erlotinib | VigiBase |
| Nephrotic syndrome | 0.41 | 1.46 | 0.12 | -0.63 | NA | 0.16 | Erlotinib | VigiBase |
| Tubulointerstitial nephritis | 0.62 | 3.06 | 0.12 | -0.31 | NA | 0.55 | Erlotinib | VigiBase |
| Azotaemia | 0.31 | 1.23 | 0.08 | -0.89 | NA | 0.08 | Erlotinib | VigiBase |
