# Supplementary material for: Renal adverse events in EGFR-TKI treatment: Comprehensive characterization of clinical patterns and molecular underpinnings
Source: Genes Dis. 2025 Nov 28;13(4):101953. doi: 10.1016/j.gendis.2025.101953 (PMC12993402; doi:10.1016/j.gendis.2025.101953)
Supplement: Table S10 — Primers used in RT-qPCR experiments in this study. [file mmc11.docx]

Supplementary table 10. qPCR primers used in this study.

| Primer name | Sequence (5’-3’) | |
| --- | --- | --- |
|  | Forward | Reverse |
| LCN-2 | GACAACCAATTCCAGGGGAAG | GCATACATCTTTTGCGGGTCT |
| KIM-1 | TCCGTGGCCCTTTTTGCTTA | GTAGTCGTGACCTTGGGTGG |
| AGT | CTCCAATTCAGGCCAAGACAT | TGTCAAGTTTTGCAGCGACTA |
| SPP1 | GAAGTTTCGCAGACCTGACAT | GTATGCACCATTCAACTCCTCG |
| IL-18 | TCTTCATTGACCAAGGAAATCGG | TCCGGGGTGCATTATCTCTAC |
| IL-1β | CCAAACCTCTTCGAGGCACA | GCTGCTTCAGACACTTGAGC |
| IL-6 | ACTCACCTCTTCAGAACGAATTG | CCATCTTTGGAAGGTTCAGGTTG |
| IL-10 | TCAAGGCGCATGTGAACTCC | GATGTCAAACTCACTCATGGCT |
| TNF-α | GAGGCCAAGCCCTGGTATG | CGGGCCGATTGATCTCAGC |
| IFN-γ | TCGGTAACTGACTTGAATGTCCA | TCGCTTCCCTGTTTTAGCTGC |
