# Supplementary material for: Renal adverse events in EGFR-TKI treatment: Comprehensive characterization of clinical patterns and molecular underpinnings
Source: Genes Dis. 2025 Nov 28;13(4):101953. doi: 10.1016/j.gendis.2025.101953 (PMC12993402; doi:10.1016/j.gendis.2025.101953)
Supplement: Table S11 — Serum biochemical test results in mouse models. [file mmc12.docx]

**Supplementary Table 11. Serum biochemical test results in mouse models.**

| **Serum biochemical test results** | | |
| --- | --- | --- |
| **Group** | **Value** | **Indicator** |
| Control | 11.30 | Crea (umol/L) |
| Control | 18.40 | Crea (umol/L) |
| Control | 12.20 | Crea (umol/L) |
| Control | 13.40 | Crea (umol/L) |
| Control | 16.40 | Crea (umol/L) |
| Control | 10.10 | Crea (umol/L) |
| Erlotinib | 19.00 | Crea (umol/L) |
| Erlotinib | 28.10 | Crea (umol/L) |
| Erlotinib | 17.50 | Crea (umol/L) |
| Erlotinib | 20.80 | Crea (umol/L) |
| Erlotinib | 19.50 | Crea (umol/L) |
| Erlotinib | 18.40 | Crea (umol/L) |
| Gefitinib | 14.40 | Crea (umol/L) |
| Gefitinib | 20.40 | Crea (umol/L) |
| Gefitinib | 19.80 | Crea (umol/L) |
| Gefitinib | 16.60 | Crea (umol/L) |
| Gefitinib | 23.20 | Crea (umol/L) |
| Gefitinib | 17.80 | Crea (umol/L) |
| Osimertinib | 16.20 | Crea (umol/L) |
| Osimertinib | 17.60 | Crea (umol/L) |
| Osimertinib | 22.80 | Crea (umol/L) |
| Osimertinib | 14.30 | Crea (umol/L) |
| Osimertinib | 17.70 | Crea (umol/L) |
| Osimertinib | 14.80 | Crea (umol/L) |
| Control | 9.58 | BUN (mmol/L) |
| Control | 7.89 | BUN (mmol/L) |
| Control | 8.35 | BUN (mmol/L) |
| Control | 8.61 | BUN (mmol/L) |
| Control | 9.07 | BUN (mmol/L) |
| Control | 7.76 | BUN (mmol/L) |
| Erlotinib | 16.90 | BUN (mmol/L) |
| Erlotinib | 13.52 | BUN (mmol/L) |
| Erlotinib | 11.57 | BUN (mmol/L) |
| Erlotinib | 11.61 | BUN (mmol/L) |
| Erlotinib | 12.72 | BUN (mmol/L) |
| Erlotinib | 12.44 | BUN (mmol/L) |
| Gefitinib | 10.46 | BUN (mmol/L) |
| Gefitinib | 8.95 | BUN (mmol/L) |
| Gefitinib | 12.37 | BUN (mmol/L) |
| Gefitinib | 9.95 | BUN (mmol/L) |
| Gefitinib | 9.81 | BUN (mmol/L) |
| Gefitinib | 9.08 | BUN (mmol/L) |
| Osimertinib | 12.53 | BUN (mmol/L) |
| Osimertinib | 10.84 | BUN (mmol/L) |
| Osimertinib | 12.99 | BUN (mmol/L) |
| Osimertinib | 13.55 | BUN (mmol/L) |
| Osimertinib | 11.28 | BUN (mmol/L) |
| Osimertinib | 13.10 | BUN (mmol/L) |
| Control | 2.40 | PHOS (mmol/L) |
| Control | 2.35 | PHOS (mmol/L) |
| Control | 2.61 | PHOS (mmol/L) |
| Control | 2.69 | PHOS (mmol/L) |
| Control | 2.56 | PHOS (mmol/L) |
| Control | 2.74 | PHOS (mmol/L) |
| Erlotinib | 2.52 | PHOS (mmol/L) |
| Erlotinib | 2.77 | PHOS (mmol/L) |
| Erlotinib | 2.75 | PHOS (mmol/L) |
| Erlotinib | 2.34 | PHOS (mmol/L) |
| Erlotinib | 2.27 | PHOS (mmol/L) |
| Erlotinib | 2.17 | PHOS (mmol/L) |
| Gefitinib | 3.39 | PHOS (mmol/L) |
| Gefitinib | 3.55 | PHOS (mmol/L) |
| Gefitinib | 3.51 | PHOS (mmol/L) |
| Gefitinib | 3.55 | PHOS (mmol/L) |
| Gefitinib | 3.06 | PHOS (mmol/L) |
| Gefitinib | 4.19 | PHOS (mmol/L) |
| Osimertinib | 2.82 | PHOS (mmol/L) |
| Osimertinib | 2.77 | PHOS (mmol/L) |
| Osimertinib | 3.17 | PHOS (mmol/L) |
| Osimertinib | 3.03 | PHOS (mmol/L) |
| Osimertinib | 3.46 | PHOS (mmol/L) |
| Osimertinib | 3.58 | PHOS (mmol/L) |
| Control | 28.20 | ALB (g/L) |
| Control | 27.80 | ALB (g/L) |
| Control | 28.00 | ALB (g/L) |
| Control | 27.30 | ALB (g/L) |
| Control | 27.80 | ALB (g/L) |
| Control | 27.40 | ALB (g/L) |
| Erlotinib | 25.00 | ALB (g/L) |
| Erlotinib | 24.50 | ALB (g/L) |
| Erlotinib | 25.80 | ALB (g/L) |
| Erlotinib | 25.10 | ALB (g/L) |
| Erlotinib | 26.70 | ALB (g/L) |
| Erlotinib | 25.00 | ALB (g/L) |
| Gefitinib | 27.00 | ALB (g/L) |
| Gefitinib | 27.50 | ALB (g/L) |
| Gefitinib | 27.80 | ALB (g/L) |
| Gefitinib | 27.70 | ALB (g/L) |
| Gefitinib | 27.50 | ALB (g/L) |
| Gefitinib | 27.70 | ALB (g/L) |
| Osimertinib | 24.80 | ALB (g/L) |
| Osimertinib | 24.40 | ALB (g/L) |
| Osimertinib | 27.20 | ALB (g/L) |
| Osimertinib | 29.10 | ALB (g/L) |
| Osimertinib | 28.50 | ALB (g/L) |
| Osimertinib | 26.70 | ALB (g/L) |
| Control | 2.12 | Ca (mmol/L) |
| Control | 2.06 | Ca (mmol/L) |
| Control | 2.11 | Ca (mmol/L) |
| Control | 2.07 | Ca (mmol/L) |
| Control | 2.12 | Ca (mmol/L) |
| Control | 2.08 | Ca (mmol/L) |
| Erlotinib | 2.00 | Ca (mmol/L) |
| Erlotinib | 2.05 | Ca (mmol/L) |
| Erlotinib | 1.92 | Ca (mmol/L) |
| Erlotinib | 1.95 | Ca (mmol/L) |
| Erlotinib | 2.00 | Ca (mmol/L) |
| Erlotinib | 1.97 | Ca (mmol/L) |
| Gefitinib | 2.04 | Ca (mmol/L) |
| Gefitinib | 1.96 | Ca (mmol/L) |
| Gefitinib | 2.08 | Ca (mmol/L) |
| Gefitinib | 2.00 | Ca (mmol/L) |
| Gefitinib | 2.13 | Ca (mmol/L) |
| Gefitinib | 2.11 | Ca (mmol/L) |
| Osimertinib | 2.07 | Ca (mmol/L) |
| Osimertinib | 1.96 | Ca (mmol/L) |
| Osimertinib | 2.07 | Ca (mmol/L) |
| Osimertinib | 2.09 | Ca (mmol/L) |
| Osimertinib | 2.07 | Ca (mmol/L) |
| Osimertinib | 2.01 | Ca (mmol/L) |
